# Disturbed engram network caused by NPTX downregulation underlies aging-related contextual fear memory deficits

**DOI:** 10.1038/s41422-025-01157-w

**Published:** 2025-08-01

**Authors:** Tao Jin, Yang Yang, Yu Guo, Yi Zhang, Qiumin Le, Nan Huang, Xing Liu, Jintai Yu, Lan Ma, Feifei Wang

**Affiliations:** 1 School of Basic Medical Sciences, State Key Laboratory of Brain Function and Disorders, MOE Frontiers Center for Brain Science, and Institutes of Brain Science, Department of Neurosurgery, Huashan Hospital, Fudan University, Shanghai, China; 2 Research Unit of Addiction Memory, Chinese Academy of Medical Sciences (2021RU009), Shanghai, China

**Keywords:** Cell biology, Mechanisms of disease

## Abstract

Engram cells storing episodic memories are allocated to separate neuronal ensembles. However, how these ensembles maintain their stability to drive precise memory expression, and whether their destabilization contributes to aging-related memory deficits, remain elusive. Here, we show that during contextual fear memory consolidation, neuronal pentraxin 1 (NPTX1) in *Fos* transcription-dependent ensemble (*F*-RAM) of the dentate gyrus (DG) promotes memory expression in the fear context. NPTX1 facilitates K_v_7.2 channel-mediated inhibition of engram cell hyperexcitability, thereby restricting the response of these cells to excitatory inputs from medial entorhinal cortex. Meanwhile, NPTX2 enhances the perisomatic inhibition of *Npas4* transcription-dependent ensemble (*N*-RAM) by parvalbumin^+^ (PV^+^) interneurons, thereby preventing fear memory overgeneralization. Pharmacological activation of K_v_7.2 channels or chemogenetic activation of PV^+^ interneurons repaired memory deficits caused by engram-specific NPTX depletion. Contextual fear memory precision and NPTX expression in DG engram cells were decreased in aged mice. Overexpressing NPTX1 in *F*-RAM ensemble or the AMPAR-binding domain of NPTX2 in *N*-RAM ensemble rescued contextual fear memory deficits. These findings elucidate that the coordination of NPTX1 and NPTX2 prevents engram ensembles from becoming hyperactive and provide a causal link between engram network destabilization and aging-related contextual fear memory deficits.

## Introduction

Specific episodic memories are believed to be encoded in sparse neuronal ensembles (engram cells) that are activated by learning experiences.^1,2^ Neurons recruited to engrams are more stably connected than those that are not.^3^ Memory accessibility and precision depend on the stabilization of engram network during memory consolidation.^4,5^ The specificity of these changes suggests a complex regulation of both excitatory and inhibitory inputs, as well as broader circuit changes. However, the molecular mechanisms underlying the maintenance of the dormant yet stable engram networks remain unknown. In addition, memory precision progressively declines with age, and is considered as one of the predominant hallmarks of aging-related cognitive dysfunctions.^6,7^ Aging-related maladaptive changes in the functional properties of neurons include both decreased and increased neuronal excitability, depending on the neuronal cell type and brain region,^8^ while the mechanistic link between the disturbed engram network and aging-related memory deficits remains to be elucidated.

Cell-adhesion molecules (CAMs) are a superfamily that play an irreplaceable role in synaptic plasticity through mediating the bidirectional organization of synaptic compartments.^9^ Neuronal pentraxins (NPTXs) are a subfamily of CAMs primarily expressed in excitatory neurons. NPTXs consist of NPTX1, NPTX2 and their receptor NPTXR.^10^ Pre-synaptically secreted NPTX1 and NPTX2 form disulfide-linked heteromultimers with post-synaptic NPTXR to promote the clustering of α-amino-3-hydroxy-5-methyl-4-isoxazolepropionic acid (AMPA) type glutamate receptors (AMPARs).^11,12^ Subsequent studies identified NPTX1 as both a pro-apoptotic protein^13^ and an inhibitor of excitatory synaptic transmission.^14^ In contrast, NPTX2 maintains excitatory homeostasis by adaptively enhancing circuit inhibition.^15,16^ These findings suggest that NPTX1 and NPTX2 may serve as potential substrates for stabilizing the excitatory and inhibitory inputs onto distinct neuronal ensembles. NPTXs are also considered as prognostic biomarkers for neurodegeneration, with a significant decrease in their expression levels in Alzheimer’s disease (AD), schizophrenia or even mild cognitive impairment patients.^17–19^ However, the causal link between NPTX reduction-induced engram network destabilization and aging-related memory deficits remain unclear.

The tracing of neuronal circuits involved in specific memory formation is enabled by utilizing the immediate early gene (IEG)-dependent robust activity marking (RAM) system.^20^ Dentate gyrus (DG) engram cells contain functionally divergent neuronal ensembles defined by *Fos* or *Npas4* activation-dependent transcriptional outputs (*F*-RAM or *N*-RAM). These two ensembles drive precise memory expression by recruiting excitatory and inhibitory (E/I) circuits.^21^ NPTX1 and NPTX2 are both expressed in DG.^22^ In this study, we found that the maintenance of engram network stability requires NPTX1 to restrict the excitatory inputs from medial entorhinal cortex (MEC), and NPTX2 to promote the inhibitory inputs from parvalbumin^+^ (PV^+^) interneurons during the consolidation of contextual fear memory. Engram network hyperactivity caused by NPTX downregulation in DG engram ensembles contributes to aging-related memory deficits. Our findings elucidate the critical role of NPTXs in stabilizing the DG engram network during memory consolidation by controlling excitatory and inhibitory inputs, and establish a causal link between engram network destabilization and aging-related memory deficits.

## Results

### NPTX1 restricts MEC excitatory inputs onto DG engram ensembles

*F*-RAM and *N*-RAM systems were employed to label engram ensembles with the mKate2 reporter after doxycycline (Dox) removal.^21^ *Npas4-CreER*^*T2*^ mice in which the endogenous *Npas4* promoter drives the expression of *CreER*^*T2*^ were generated to label *Npas4*^+^ ensemble with the EGFP reporter by 4-hydroxytamoxifen (4-OHT) intraperitoneal (i.p.) injection (Supplementary information, Fig. S1a–d). A combination of these two activity-dependent labeling strategies (Supplementary information, Fig. S1e) was used simultaneously to label engrams activated by contextual fear conditioning (CFC, 0.5 mA, 1 s, 3 trials). The EGFP^+^ neurons exhibited substantial overlap with mKate2^+^ *N*-RAM ensemble but minimal colocalization with mKate2^+^ *F*-RAM ensemble (Supplementary information, Fig. S1f, g). These results confirm that engram cells in DG labeled by *F*-RAM and *N*-RAM reporters are distinct populations.

**Fig. 1:**
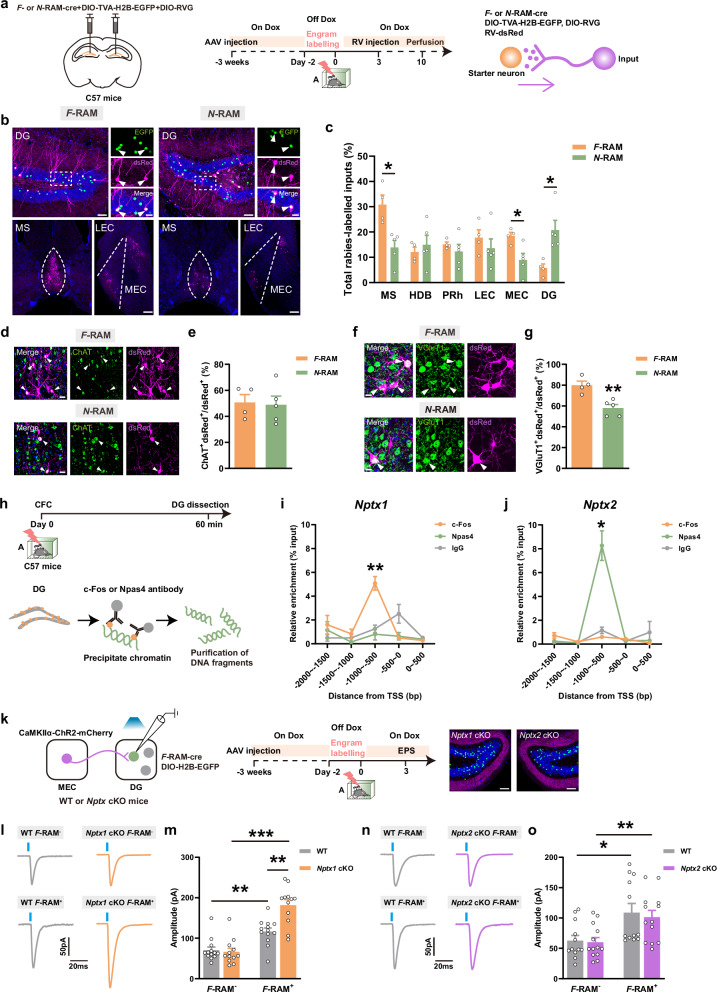
RV tracing of the upstream inputs to DG *F*- and *N*-RAM neuronal ensembles and the effects of *Nptx* depletion on the response to MEC excitatory inputs. **a** Diagram of virus injection and experimental scheme to trace upstream inputs of DG *F*-RAM and *N*-RAM ensembles. **b** Representative confocal images of DG starter neurons in *F*-RAM and *N*-RAM ensembles and their respective upstream inputs. Green: *F*-RAM or *N*-RAM engram cells, EGFP; purple: dsRed; blue: DAPI. White arrows indicate the starter neurons. Scale bars: top left, 50 μm; top right, 10 μm; bottom, 100 μm. **c** Percentages of total inputs from each upstream site relative to total quantified inputs. *F*-RAM, *n* = 4 mice; *N*-RAM, *n* = 5 mice. **d** Representative confocal images of upstream MS dsRed^+^ cells immunostained with ChAT. Green: ChAT; purple: dsRed; blue: DAPI. White arrows indicate the colocalized cells. Scale bars, 10 μm. **e** Percentages of colocalized cells in MS total dsRed^+^ cells. *F*-RAM, *n* = 4 mice; *N*-RAM, *n* = 5 mice. **f** Representative confocal images of upstream MEC dsRed^+^ cells immunostained with vGluT1. Green: vGluT1; purple: dsRed; blue: DAPI. White arrows indicate the colocalized cells. Scale bars, 5 μm. **g** Percentages of colocalized cells in MEC total dsRed^+^ cells. *F*-RAM, *n* = 4 mice; *N*-RAM, *n* = 5 mice. **h** Experimental scheme of ChIP assay in DG. **i** ChIP-qPCR analysis showing the enrichment of c-Fos and Npas4 at genomic sequences located from –2000 bp upstream to 500 bp downstream of *Nptx1* TSS. c-Fos, *n* = 4 from 12 mice; Npas4, *n* = 4 from 12 mice; IgG, *n* = 4 from 12 mice. **j** ChIP-qPCR analysis showing the enrichment of c-Fos and Npas4 at genomic sequences located from –2000 bp upstream to 500 bp downstream of *Nptx2* TSS. c-Fos, *n* = 4 from 12 mice; Npas4, *n* = 3 from 9 mice; IgG, *n* = 4 from 12 mice. **k** Diagram of AAV injection, experimental scheme and representative expression of ChR2 in MEC projection neurons and DG engram cells. Green: *F*-RAM engram cells of *Nptx1* and *Nptx2* cKO mice, EGFP; purple: MEC projections, mCherry; blue: DAPI. Scale bars, 50 μm. **l**, **n** Representative traces of oEPSC recorded from WT and *Nptx* cKO mice. **m** Quantification of oEPSC amplitudes recorded from WT and *Nptx1* cKO mice. WT-*F*-RAM^–^, *n* = 13 neurons from 4 mice; *Nptx1* cKO-*F*-RAM^–^, *n* = 12 neurons from 4 mice; WT-*F*-RAM^+^, *n* = 13 neurons from 4 mice; *Nptx1* cKO-*F*-RAM^+^, *n* = 12 neurons from 4 mice. **o** Quantification of oEPSC amplitudes recorded from WT and *Nptx2* cKO mice. WT-*F*-RAM^–^, *n* = 12 neurons from 4 mice; *Nptx2* cKO-*F*-RAM^–^, *n* = 13 neurons from 4 mice; WT-*F*-RAM^+^, *n* = 12 neurons from 4 mice; *Nptx2* cKO-*F*-RAM^+^, *n* = 13 neurons from 4 mice. Data are presented as mean ± SEM; *P < 0.05, **P < 0.01, ***P < 0.001.

*AAV-F-RAM* and *AAV-N-RAM* promoters driving expression of Cre recombinase *(AAV-F-RAM-Cre* and *AAV-N-RAM-Cre)* were generated. The number of tagged *F*- or *N*-RAM engram cells was significantly increased in the off Dox-CFC group (Supplementary information, Fig. S2). The upstream inputs of *F*- and *N*-RAM neuronal ensembles from medial septum (MS), horizontal limb of the diagonal band (HDB), perirhinal cortex (PRh), lateral entorhinal cortex (LEC), MEC and DG were detected by rabies virus (RV) tracing (Fig. 1a, b). *F*-RAM ensemble formed more connections with MS and MEC, while *N*-RAM ensemble received more inputs from DG local neurons (Fig. 1c). Nearly half of the dsRed^+^ inputs from MS to *F*-RAM and *N*-RAM neurons expressed the cholinergic neuron marker, choline acetyltransferase (ChAT) (Fig. 1d, e). However, *F*-RAM neurons received more excitatory inputs from MEC than *N*-RAM neurons (Fig. 1f, g).

NPTXs were found to be predominantly involved in E/I synaptic homeostasis rather than cholinergic signaling.^12,23^ Chromatin immunoprecipitation-quantitative PCR (ChIP-qPCR) on the DG of mice was performed 1 h after fear conditioning using anti-c-Fos and anti-Npas4 antibodies (Fig. 1h). C-Fos was preferentially enriched in the transcription starting site (TSS) of *Nptx1* ranging from –1000 bp to –500 bp, whereas Npas4 was preferentially enriched in the TSS of *Nptx2* ranging from –1000 bp to –500 bp (Fig. 1i, j), suggesting that NPTX1 and NPTX2 could be preferentially recruited by Fos and Npas4 to regulate the plasticity of the formed engram circuits, respectively.

*Nptx1*^*fl/fl*^, *Nptx2*^*fl/fl*^ conditional knockout (*Nptx1* cKO, *Nptx2* cKO) mice were generated (Supplementary information, Fig. S3a, b). Ribosome-associated transcripts of *F*-RAM and *N*-RAM ensembles were enriched from *Nptx* cKO mice and their wild-type (WT) littermates 3 days after CFC (Supplementary information, Fig. S3c, d), and the knockout efficiency of *Nptx1* and *Nptx2* in *F-* or *N-*RAM ensembles were verified by qPCR. *Nptx2* expression remained unaffected when *Nptx1* was depleted, and vice versa (Supplementary information, Fig. S3e–l). The expression of NPTX1 and NPTX2 proteins in *F*-RAM and *N*-RAM ensembles were examined 1.5 h, 6 h, 24 h and 72 h after CFC. Compared with the WT groups, NPTX proteins in engram cells of the cKO groups were decreased at 24 h and 72 h (Supplementary information, Figs. S4, S5). The delayed effect of Cre-dependent knockout does not affect NPTX expression during fear conditioning; rather, it exerts an effect during memory consolidation, once the engram circuits have formed.

To test the role of NPTXs in the excitatory connection between MEC and DG *F-*RAM ensemble, *AAV-CaMKIIα-ChR2-mCherry* was injected into MEC of *Nptx1* cKO, *Nptx2* cKO mice and their WT littermates, and the virus mixture of *AAV-F-RAM-Cre* with Cre-dependent *AAV-DIO-H2B-EGFP* was injected into DG to knock out *Nptxs* in *F-*RAM ensemble. Opto-evoked excitatory post-synaptic currents (oEPSCs) from MEC were recorded on EGFP^–^ (*F*-RAM^–^) and EGFP^+^ (*F*-RAM^+^) cells (Fig. 1k). The amplitude of oEPSCs recorded from EGFP^+^ cells was larger in the *Nptx1* cKO group (Fig. 1l, m), whereas this effect was not found in the *Nptx2* cKO mice (Fig. 1n, o). These data indicate that NPTX1 plays an important role in dampening the excitatory synaptic transmission between MEC and DG *F-*RAM ensemble.

### NPTX1 facilitates M-current and membrane expression of K_v_7.2 in DG *F*-RAM ensemble

**Fig. 2:**
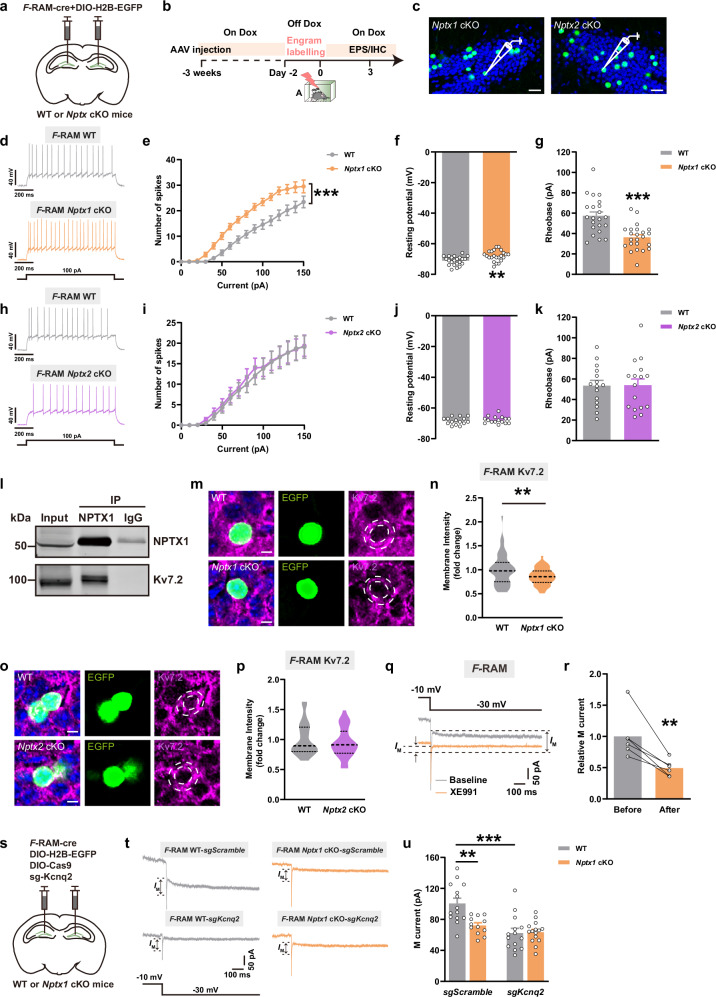
The effects of *Nptx1* or *Nptx2* depletion on engram excitability and membrane expression of K_v_7.2. **a** Diagram of AAV injection. **b** Experimental scheme to label *F*-RAM and *N*-RAM engram ensembles. EPS, electrophysiology; IHC, immunohistochemistry. **c** Representative images of *F*-RAM engram cells in DG of *Nptx1* and *Nptx2* cKO mice. Green: *F*-RAM engram cells of *Nptx1* and *Nptx2* cKO mice, EGFP; blue: DAPI. Scale bars, 20 μm. **d**, **h** Representative AP traces induced by depolarizing current injections (100 pA) recorded from WT and *Nptx* cKO mice. **e** Input-output curves of AP spikes vs injected currents of WT and *Nptx1* cKO mice. WT, *n* = 22 neurons from 4 mice; *Nptx1* cKO, *n* = 23 neurons from 4 mice. **f** RMP of WT and *Nptx1* cKO mice. WT, *n* = 22 neurons from 4 mice; *Nptx1* cKO, *n* = 23 neurons from 4 mice. **g** Rheobase of WT and *Nptx1* cKO mice. WT, *n* = 22 neurons from 4 mice; *Nptx1* cKO, *n* = 23 neurons from 4 mice. **i** Input-output curves of AP spikes vs injected currents of WT and *Nptx2* cKO mice. WT, *n* = 15 neurons from 4 mice; *Nptx2* cKO, *n* = 16 neurons from 3 mice. **j** RMP of WT and *Nptx2* cKO mice. WT, *n* = 15 neurons from 4 mice; *Nptx2* cKO, *n* = 16 neurons from 3 mice. **k** Rheobase of WT and *Nptx2* cKO mice. WT, *n* = 15 neurons from 4 mice; *Nptx2* cKO, *n* = 16 neurons from 3 mice. **l** Co-IP of NPTX1 with K_v_7.2 in DG. **m**, **o** Representative confocal images of engram cells colocalizing with K_v_7.2. Green: *F*-RAM engram cells, EGFP; purple: K_v_7.2; blue: DAPI. Dashed white lines outline cell membrane. Scale bars, 5 μm. **n** Quantification of membrane K_v_7.2 fluorescence intensity of WT and *Nptx1* cKO neurons. WT, *n* = 53 neurons from 5 mice; *Nptx1* cKO, *n* = 48 neurons from 6 mice. **p** Quantification of K_v_7.2 fluorescence intensity of WT and *Nptx2* cKO neurons. WT, *n* = 38 neurons from 5 mice; *Nptx1* cKO, *n* = 33 neurons from 6 mice. **q** Representative traces of *I*_M_ current recorded before and after application of XE991. **r** Normalized *I*_M_ current recorded at –30 mV before and after application of XE991. *n* = 6 neurons from 3 mice. **s** Diagram of AAV injection. **t** Representative traces of *I*_M_ current recorded from WT and *Nptx1* cKO mice with or without *Kcnq2* knockdown. **u** Average *I*_M_ current amplitudes recorded at –30 mV from WT and *Nptx1* cKO mice with or without *Kcnq2* knockdown. WT-*sgScramble*, *n* = 14 neurons from 3 mice; *Nptx1* cKO-*sgScramble*, *n* = 12 neurons from 3 mice; WT-*sgKcnq2*, *n* = 14 neurons from 3 mice; *Nptx1* cKO-*sgKcnq2*, *n* = 15 neurons from 3 mice. Data are presented as mean ± SEM; **P < 0.01, ***P < 0.001.

Action potential (AP) recordings were performed to examine whether neuronal excitability of the engram ensembles was changed after *Nptx* knockout (Fig. 2a–c). *F*-RAM engram cells lacking *Nptx1*, but not *Nptx2*, exhibited upward shift of the neuronal spiking curve after step-increment current injections, lower resting membrane potential (RMP) and rheobase (Fig. 2d–k), indicating increased excitability of *F*-RAM engram cells. KCNQ2 (K_v_7.2) pairs with the KCNQ3 (K_v_7.3) subunit to form KCNQ2/3 heterotetramers, which are the primary molecular subunits of M-channel involved in regulating neuronal excitability.^24,25^ Co-immunoprecipitation (co-IP) of the DG lysate confirmed the interaction between NPTX1 and K_v_7.2 (Fig. 2l). Immunostaining analysis further revealed that *Nptx1* ablation decreased K_v_7.2 membrane expression in *F*-RAM cells (Fig. 2m, n), which was not observed in *Nptx2* cKO group (Fig. 2o, p). Application of the KCNQ2/3-selective blocker XE991 resulted in a reduction of M-current (*I*_M_) amplitude on DG *F*-RAM engram cells (Fig. 2q, r). *Nptx1* cKO exerted the similar inhibitory effect to XE991 on *I*_M_ amplitude, while this effect was blunted when *Kcnq2* was simultaneously knocked down in *Nptx1* cKO *F*-RAM ensemble by using *CRISPR-Cas9-Kcnq2-sgRNA*^25^ (Fig. 2s–u). Taken together, these results indicate that NPTX1 restrains the excitatory synaptic transmission between MEC and DG *F-*RAM ensemble by facilitating K_v_7.2-mediated inhibition of neuronal hyperexcitability.

### NPTX2 in *N*-RAM ensemble facilitates the perisomatic inhibition by PV^+^ interneurons in DG

The results of RV tracing (Fig. 1c) and ChIP-qPCR (Fig. 1j) led us to wonder whether NPTX2 was specifically involved in *N*-RAM ensemble-dependent inhibitory synaptic connection in DG. PV^+^ and somatostatin^+^ (SST^+^) interneurons are two major subtypes of GABAergic interneurons in DG^26^ that separately target perisomatic compartments and distal apical dendrites of granule cells.^27^ *F*-RAM and *N*-RAM ensembles received equal amount of SST^+^ inputs (Supplementary information, Fig. S6a). To test the involvement of NPTXs in the synaptic transmission between *N*-RAM ensemble and SST^+^ interneurons, the viral mixture of *AAV-N-RAM-Cre*, *AAV-DIO-ChR2-EYFP*, *AAV-SST-Flp* and Flp recombinase-dependent *AAV-fDIO-mCherry* was injected into DG of *Nptx1* cKO, *Nptx2* cKO mice and their WT littermates. The specificity of *AAV-SST-Flp*^28,29^ was confirmed by immunostaining with anti-SST antibody (Supplementary information, Fig. S6c, d). Opto-evoked paired-pulse ratio (PPR) and AMPAR/*N*-methyl-D-aspartate receptor (NMDAR) (A/N) ratio were recorded in SST^+^ interneurons according to mCherry expression, morphology and location (Supplementary information, Fig. S6e, f). Depletion of *Nptxs* in *N*-RAM ensemble induced increased PPR of SST^+^ interneurons (Supplementary information, Fig. S6g, h, k, l), consistent with previous studies that NPTX family promotes pre-synaptic glutamate release.^23,30^ However, *Nptx* knockout in *N*-RAM ensemble exerted no effect on A/N ratio of SST^+^ interneurons (Supplementary information, Fig. S6i, j, m, n).

**Fig. 3:**
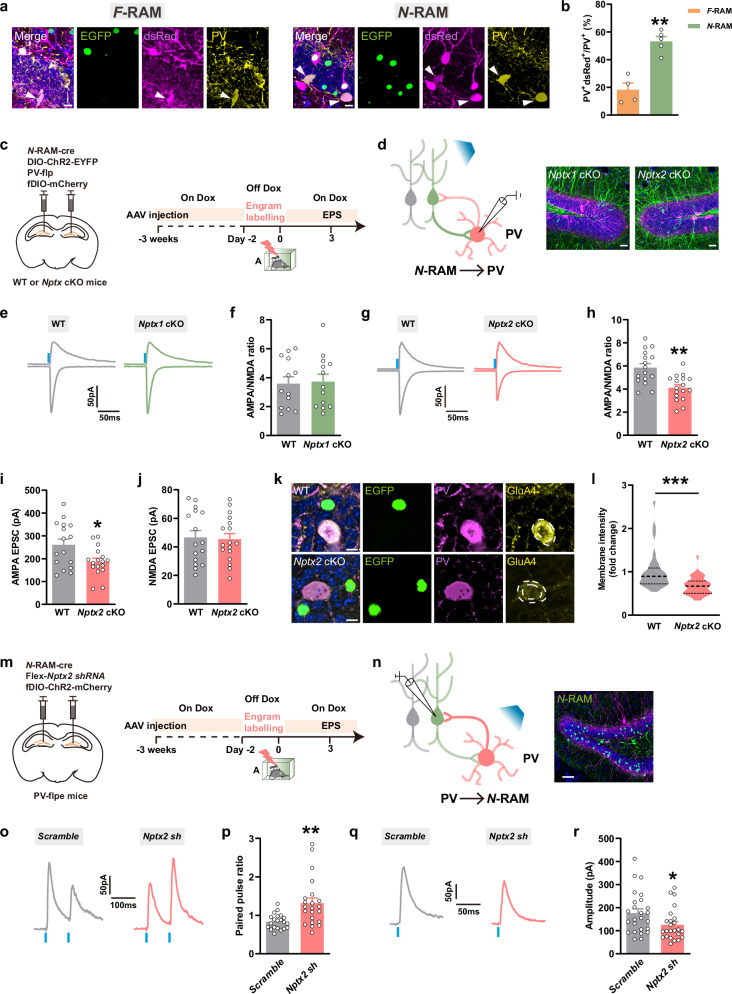
The effects of *Nptx2* depletion on the functional connection between DG local PV^+^ interneurons and *N*-RAM ensemble. **a** Representative confocal images of DG local dsRed^+^ cells immunostained with PV. Green: EGFP; purple: dsRed; yellow: PV; blue: DAPI. White arrows indicate the colocalized cells. Scale bars, 5 μm. **b** Percentages of colocalized cells in DG total PV^+^ cells. *F*-RAM, *n* = 4 mice; *N*-RAM, *n* = 5 mice. **c** Diagram of AAV injection and experimental scheme to label *N*-RAM engram ensembles. **d** Diagram of photostimulation and whole-cell patch clamp recordings (left) and representative images of engram cells and PV^+^ interneurons (right). Green: *N*-RAM engram cells of *Nptx1* and *Nptx2* cKO mice, EYFP; purple: PV^+^ interneurons, mCherry; blue: DAPI. Scale bars, 10 μm. **e**, **g** Representative traces of opto-evoked AMPA-EPSC and NMDA-EPSC recorded from WT and *Nptx* cKO mice. **f** Average A/N ratio recorded from WT and *Nptx1* cKO mice. WT, *n* = 13 neurons from 4 mice; *Nptx1* cKO, *n* = 13 neurons from 4 mice. **h** Average A/N ratio recorded from WT and *Nptx2* cKO mice. WT, *n* = 16 neurons from 3 mice; *Nptx2* cKO, *n* = 17 neurons from 5 mice. **i**, **j** Average AMPA-EPSC (**i**) and NMDA-EPSC (**j**) amplitudes recorded from WT and *Nptx2* cKO mice. WT, *n* = 16 neurons from 3 mice; *Nptx2* cKO, *n* = 17 neurons from 5 mice. **k** Representative confocal images of GluA4 colocalizing with PV^+^ interneurons. Green: *N*-RAM engram cells; purple: PV^+^ interneurons; yellow: GluA4. Scale bars, 10 μm. **l** Quantification of GluA4 membrane expression on PV^+^ interneurons in WT and *Nptx2* cKO mice. WT, *n* = 32 neurons from 5 mice; *Nptx2* cKO, *n* = 37 neurons from 5 mice. **m** Diagram of AAV injection and experimental scheme to label *N*-RAM engram ensembles. **n** Diagram of photostimulation and whole-cell patch clamp recordings (left) and representative images of *N*-RAM engram cells and PV^+^ interneurons (right). Green: *N*-RAM engram cells, EGFP; purple: PV^+^ interneurons, mCherry; blue: DAPI. Scale bar, 20 μm. **o**, **q** Representative traces of opto-evoked PPR (**o**) and oIPSC (**q**) recorded from neurons transfected with Scramble or *Nptx2* shRNA. **p**, **r** Quantification of PPR (**p**) and oIPSC (**r**) amplitudes recorded from neurons transfected with Scramble or *Nptx2* shRNA. For PPR recording, Scramble, *n* = 21 from 4 mice; *Nptx2* shRNA, *n* = 22 from 4 mice. For oIPSC recording, Scramble, *n* = 25 from 4 mice; *Nptx2* shRNA, *n* = 24 from 4 mice. Data are presented as mean ± SEM; *P < 0.05, **P < 0.01, ***P < 0.001.

*N-*RAM neurons received more PV^+^ inputs than *F-*RAM neurons in DG (Fig. 3a, b). To investigate the involvement of NPTX2 in the synaptic transmission between *N*-RAM ensemble and PV^+^ interneurons, the viral mixture of *AAV-N-RAM-Cre*, *AAV-DIO-ChR2-EYFP*, *AAV-PV-Flp* and *AAV-fDIO-mCherry* was injected into DG of *Nptx1* cKO, *Nptx2* cKO mice and their WT littermates. The specificity of *AAV-PV-Flp*^28,31^ was confirmed by immunostaining with anti-PV antibody (Supplementary information, Fig. S7a). PPR and A/N ratio were recorded in PV^+^ interneurons (Fig. 3c, d). The weakened pre-synaptic glutamate release was observed in both *Nptx1* cKO and *Nptx2* cKO mice (Supplementary information, Fig. S7b–e), while decreased AMPAR-mediated EPSC and A/N ratio in PV^+^ interneurons were only found in *Nptx2* cKO mice (Fig. 3e–j). Immunostaining showed that *Nptx2* depletion in *N*-RAM ensemble reduced the membrane expression of AMPAR subunit GluA4 in PV^+^ interneurons (Fig. 3k, l), which is consistent with previous findings that NPTX2 binds GluA4 in PV^+^ interneurons to regulate the network excitatory/inhibitory balance.^15,32^ Moreover, depletion of *Nptxs* in *F*-RAM ensemble exerted no effect on the A/N ratios of postsynaptic PV^+^ or SST^+^ interneurons (Supplementary information, Fig. S8). These data demonstrate that NPTX2 is specifically involved in mediating the plasticity of the circuits between PV^+^ interneurons and the *N*-RAM ensemble in DG. To examine whether NPTX2-dependent plasticity also applies to GABAergic CCK^+^ cells, the viral mixture of *AAV-N-RAM-Cre*, *AAV-DIO-ChR2-EYFP* with *AAV-vGAT2-Flp*^33–35^-dependent *AAV-CCK-fDIO-mCherry* was injected into DG of WT and *Nptx2* cKO mice to label the GABAergic CCK^+^ cells (Supplementary information, Fig. S9a–c). Opto-evoked PPR and A/N ratio of mCherry^+^ CCK^+^ interneurons were unaffected by *Nptx2* depletion in *N*-RAM ensemble (Supplementary information, Fig. S9d–g). These data further suggest that NPTX2-dependent interneuron plasticity is specific to PV^+^ interneurons.

The disturbed PV^+^ interneuron plasticity caused by *Nptx2* depletion in *N*-RAM ensemble led us to speculate that the inhibitory inputs received by the *N*-RAM ensemble was affected. A viral mixture of *AAV-N-RAM-Cre*, *AAV-Flex-EGFP-Nptx2 shRNA* (*Nptx2 sh*)^22^ or *Scramble shRNA* (*Scramble*), and *AAV-fDIO-ChR2-mCherry* was injected into DG of *PV-Flpe* mice. Opto-evoked inhibitory post-synaptic currents (oIPSCs) and PPR were then recorded in *N*-RAM cells (Fig. 3m, n). *Nptx2* knockdown in *N*-RAM ensemble increased PPR (Fig. 3o, p) and decreased IPSC amplitude evoked by optical activation of PV^+^ interneurons (Fig. 3q, r). These data indicate a reduction in pre-synaptic γ-aminobutyric acid (GABA) release and the weakened inhibitory inputs to *N-*RAM engram cells. Taken together, these data indicate that NPTX2 in *N*-RAM ensemble facilitates the inhibitory synaptic transmission between DG *N*-RAM engram cells and PV^+^ interneurons by the AMPAR-dependent mechanism.

### NPTX1 in DG *F*-RAM ensemble and NPTX2 in DG *N*-RAM ensemble facilitate the precise expression of contextual fear memory

**Fig. 4:**
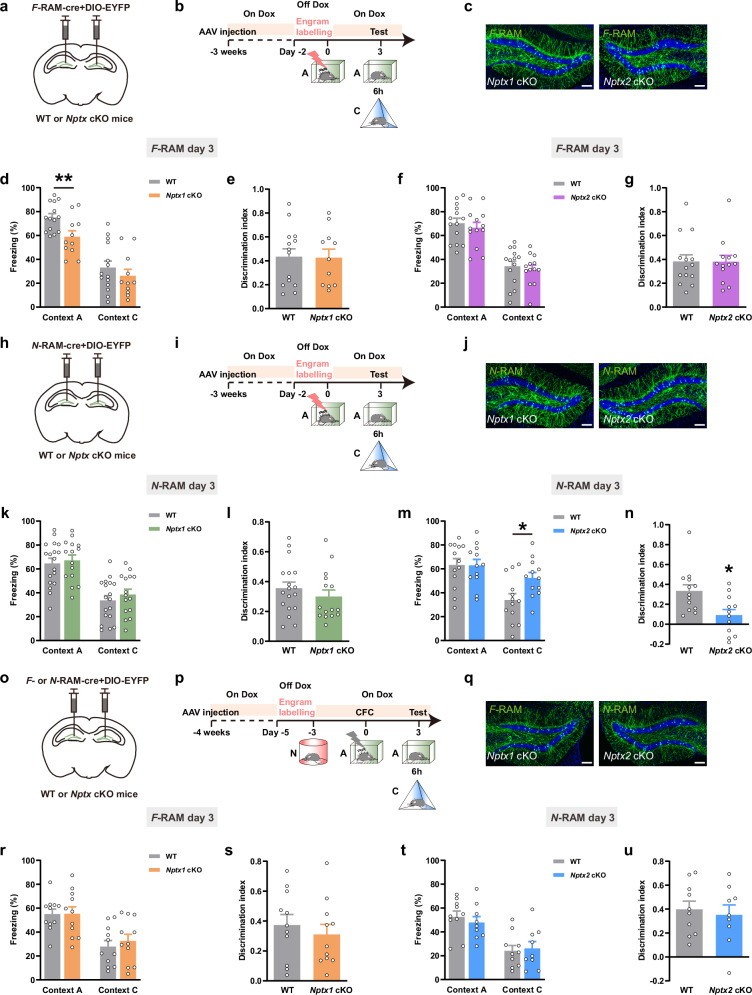
The effects of *Nptx* depletion in *F*-RAM and *N*-RAM ensembles on the expression of contextual fear memory. **a**, **h**, **o** Diagram of AAV injection. **b**, **i**, **p** Experimental scheme of CFC and memory retrieval. **c**, **j**, **q** Representative images of *F*- and *N*-RAM engram cells in DG. Green: *F*-RAM or *N*-RAM ensemble, EYFP; blue: DAPI. Scale bar, 100 μm. **d**, **e** Freezing percentage (**d**) and discrimination index (**e**) of WT and *Nptx1* cKO mice tested in context A and context C at day 3 (*F*-RAM). WT, *n* = 14 mice; *Nptx1* cKO, *n* = 11 mice. **f**, **g** Freezing percentage (**f**) and discrimination index (**g**) of WT and *Nptx2* cKO mice tested in context A and context C at day 3 (*F*-RAM). WT, *n* = 14 mice; *Nptx2* cKO, *n* = 13 mice. **k**, **l** Freezing percentage (**k**) and discrimination index (**l**) of WT and *Nptx1* cKO mice tested in context A and context C at day 3 (*N*-RAM). WT, *n* = 18 mice; *Nptx1* cKO, *n* = 15 mice. **m**, **n** Freezing percentage (**m**) and discrimination index (**n**) of WT and *Nptx2* cKO mice tested in context A and context C at day 3 (*N*-RAM). WT, *n* = 13 mice; *Nptx2* cKO, *n* = 12 mice. **r**, **s** Freezing percentage (**r**) and discrimination index (**s**) of WT and *Nptx1* cKO mice tested in context A and context C at day 3 (*F*-RAM). WT, *n* = 11 mice; *Nptx1* cKO, *n* = 11 mice. **t**, **u** Freezing percentage (**t**) and discrimination index (**u**) of WT and *Nptx2* cKO mice tested in context A and context C at day 3 (*N*-RAM). WT, *n* = 10 mice; *Nptx2* cKO, *n* = 9 mice. Data are presented as mean ± SEM; *P < 0.05.

To investigate the role of NPTX-dependent maintenance of engram network stability in regulating specific behavioral outputs, the viral mixture of *AAV-F-RAM-Cre* or *AAV-N-RAM-Cre* combined with *AAV-DIO-EYFP* was injected into DG of young *Nptx1* cKO, *Nptx2* cKO mice and their respective WT littermates. Mice were tested on day 3 in fear context A and non-fear context C after CFC (Fig. 4a–c, h–j). The freezing levels during conditioning were not different between *Nptx1* cKO, *Nptx2* cKO mice and their WT littermates (Supplementary information, Fig. S10). Interestingly, *Nptx1* depletion in *F*-RAM ensemble decreased freezing in context A (Fig. 4d, e; Supplementary information, Fig. S11a–f), whereas *Nptx2* depletion in *F*-RAM ensemble caused no difference in freezing levels in either context (Fig. 4f, g). *Nptx1* knockout in DG *N*-RAM ensemble did not affect the freezing levels in either context (Fig. 4k, l), whereas *Nptx2* knockout in DG *N*-RAM ensemble increased the freezing levels in context C on day 3 (Fig. 4m, n; Supplementary information, Fig S11k–p), indicating overgeneralization of contextual fear memory. Other types of memories, such as novel object recognition (NOR) and novel place recognition (NPR) were preserved when *Nptxs* were knocked out in *F*-RAM and *N*-RAM neurons (Supplementary information, Fig. S11g–j, q–t).

Furthermore, knocking down *Nptx1* or *Nptx2* in *F*- or *N*-RAM neurons (activated during exposure to a novel context N, distinct from context A or C) did not significantly alter the freezing levels in *Nptx* cKO mice compared to WT controls (Fig. 4o–u), indicating that NPTXs exert a fear context-specific regulatory effect on memory precision. In addition, *Nptx* depletion in both ensembles had no effect on mouse’s locomotion, anxiety or depression levels (Supplementary information, Figs. S12–S14).

Taken together, these data suggest that the precise expression of contextual fear memory, characterized by high freezing levels in the fear context and low levels in the non-fear context, requires the coordinated function of NPTX1 and NPTX2 in distinct ensembles.

### Pharmacological activation of K_v_7.2 channels or chemogenetic activation of PV^+^ interneurons ameliorates contextual fear memory deficits induced by NPTX depletion in DG engrams

**Fig. 5:**
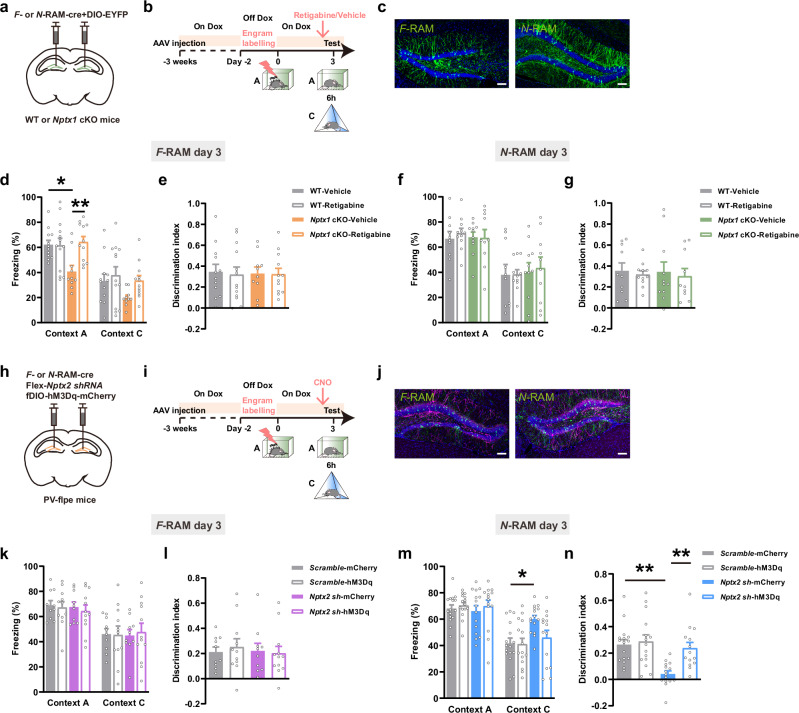
The effects of activating K_v_7.2 or DG PV^+^ interneurons on memory deficits induced by *Nptx* depletion in DG engram ensembles. **a**, **h** Diagram of AAV injection. **b**, **i** Experimental scheme of memory retrieval test. **c** Representative images of *F*- and *N*-RAM engram cells in DG. Green: *F*-RAM and *N*-RAM ensembles, EYFP; blue: DAPI. Scale bars, 100 μm. **d**, **e** Freezing percentage (**d**) and discrimination index (**e**) of WT-Vehicle, WT-Retigabine, *Nptx1* cKO-Vehicle and *Nptx1* cKO-Retigabine mice (*F*-RAM). WT-Vehicle, *n* = 12 mice; WT-Retigabine, *n* = 13 mice; *Nptx1* cKO-Vehicle, *n* = 10 mice; *Nptx1* cKO-Retigabine, *n* = 12 mice. **f**, **g** Freezing percentage (**f**) and discrimination index (**g**) of WT-Vehicle, WT-Retigabine, *Nptx1* cKO-Vehicle and *Nptx1* cKO-Retigabine mice (*N*-RAM). WT-Vehicle, *n* = 10 mice; WT-Retigabine, *n* = 11 mice; *Nptx1* cKO-Vehicle, *n* = 11 mice; *Nptx1* cKO-Retigabine, *n* = 10 mice. **j** Representative images of *F*- and *N*-RAM engram cells in DG. Green: *F*-RAM and *N*-RAM ensembles, EGFP; purple: PV^+^ interneurons, mCherry; blue: DAPI. Scale bars, 100 μm. **k**, **l** Freezing percentage (**k**) and discrimination index (**l**) of *Scramble*-mCherry, *Scramble*-hM3Dq, *Nptx2 sh*-mCherry and *Nptx2 sh*-hM3Dq groups (*F*-RAM). *Scramble*-mcherry, *n* = 10 mice; *Scramble*-hM3Dq, *n* = 11 mice; *Nptx2* sh-mcherry, *n* = 10 mice; *Nptx2* sh-hM3Dq, *n* = 12 mice. **m**, **n** Freezing percentage (**m**) and discrimination index (**n**) of *Scramble*-mCherry, *Scramble*-hM3Dq, *Nptx2 sh*-mCherry and *Nptx2 sh*-hM3Dq groups (*N*-RAM). *Scramble*-mcherry, *n* = 16 mice; *Scramble*-hM3Dq, *n* = 15 mice; *Nptx2* sh-mcherry, *n* = 15 mice; *Nptx2* sh-hM3Dq, *n* = 14 mice. Data are presented as mean ± SEM; *P < 0.05, **P < 0.01.

The K_v_7 activator retigabine (a clinically used anticonvulsant^36^), was injected intraperitoneally 30 min before mice were tested in the fear context A and non-fear context C (Fig. 5a–c). Retigabine had no effect on the freezing levels of WT mice in either context, whereas it restored the reduced freezing levels in context A when NPTX1 was depleted in *F*-RAM ensemble (Fig. 5d–g; Supplementary information, Fig. S15a, b).

To restore the activity of PV^+^ interneurons after *Nptx2* depletion in *N*-RAM ensemble, the designer receptors exclusively activated by designer drugs (DREADDs) were used to activate PV^+^ interneurons. The viral mixture of *AAV-N-RAM-Cre*, *AAV-Flex-EGFP-Nptx2 shRNA* and *AAV-fDIO-hM3Dq-mCherry* was injected into the DG of *PV-Flpe* mice and clozapine N-oxide (CNO) was administered via i.p. injection 30 min before memory test (Fig. 5h–j). Chemogenetic activation of PV^+^ interneurons in DG did not affect either freezing levels or discrimination index in control groups expressing Scramble shRNA, but increased the discrimination index when *Nptx2* was knocked down in *N*-RAM ensemble (Fig. 5k–n; Supplementary information, Fig. S15c, d).

These data suggest that the deficits in contextual fear memory induced by NPTX depletion in distinct DG engram ensembles can be ameliorated by activation of K_v_7.2 channels or DG PV^+^ interneurons.

### Downregulation of NPTX1 and NPTX2 in DG engram cells in aged mice

**Fig. 6:**
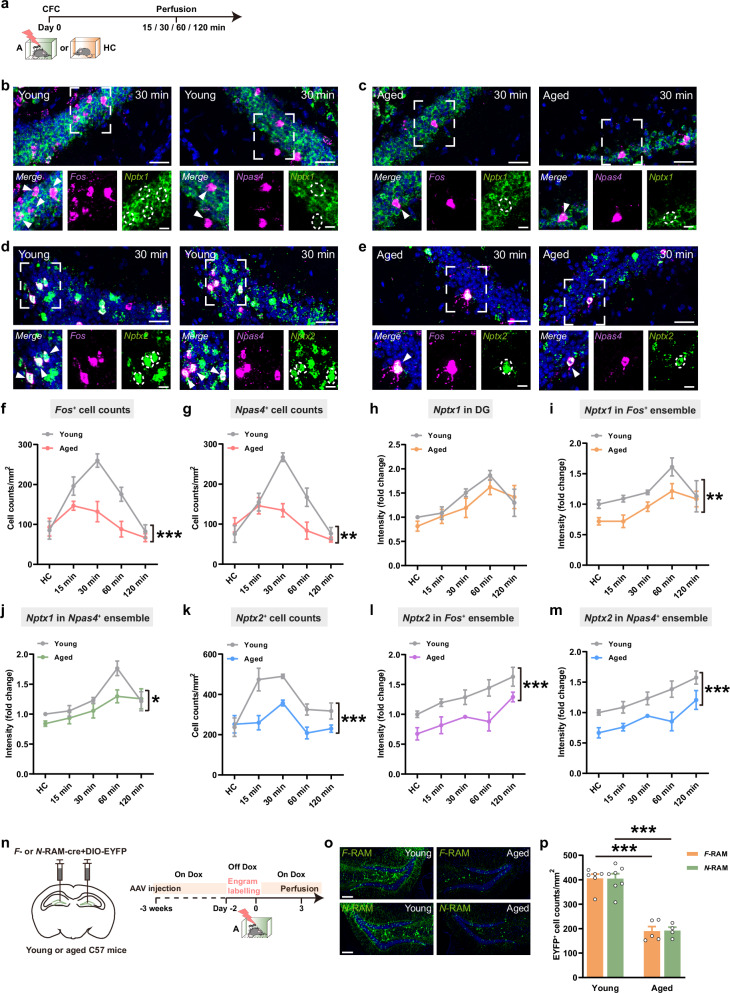
*Nptx* expression in DG engram cells in young and aged mice. **a** Experimental scheme for smFISH. **b**, **c** Representative confocal images of *Nptx1* colocalizing with *Fos* and *Npas4* 30 min after CFC in DG of young (**b**) and aged (**c**) mice. Green: *Nptx1*; purple: *Fos*; blue: DAPI. White arrows indicate the colocalized cells. Scale bars: top, 30 μm; bottom, 10 μm. **d**, **e** Representative confocal images of *Nptx2* colocalizing with *Fos* and *Npas4* 30 min after CFC in DG of young (**d**) and aged (**e**) mice. Green: *Nptx2*; purple: *Npas4*; blue: DAPI. White arrows indicate the colocalized cells. Scale bars: top, 30 μm; bottom, 10 μm. **f**, **g** *Fos*^*+*^ (**f**) and *Npas4*^*+*^ (**g**) cell counts in DG. Young HC, *n* = 4 mice, Aged HC, *n* = 5 mice; Young 15 min, *n* = 5 mice, Aged 15 min, *n* = 5 mice; Young 30 min, *n* = 4 mice, Aged 30 min, *n* = 4 mice; Young 60 min, *n* = 4 mice, Aged 60 min, *n* = 5 mice; Young 120 min, *n* = 4 mice, Aged 120 min, *n* = 5 mice. **h** Fluorescence intensity of overall *Nptx1* mRNA in DG. Young HC, *n* = 4 mice, Aged HC, *n* = 4 mice; Young 15 min, *n* = 5 mice, Aged 15 min, *n* = 5 mice; Young 30 min, *n* = 5 mice, Aged 30 min, *n* = 4 mice; Young 60 min, *n* = 5 mice, Aged 60 min, *n* = 5 mice; Young 120 min, *n* = 4 mice, Aged 120 min, *n* = 5 mice. **i** Fluorescence intensity of *Nptx1* mRNA in *Fos*^*+*^ ensemble in DG. Young HC, *n* = 4 mice, Aged HC, *n* = 4 mice; Young 15 min, *n* = 5 mice, Aged 15 min, *n* = 5 mice; Young 30 min, *n* = 5 mice, Aged 30 min, *n* = 4 mice; Young 60 min, *n* = 5 mice, Aged 60 min, *n* = 5 mice; Young 120 min, *n* = 4 mice, Aged 120 min, *n* = 5 mice. **j** Fluorescence intensity of *Nptx1* mRNA in *Npas4*^*+*^ ensemble in DG. Young HC, *n* = 5 mice, Aged HC, *n* = 4 mice; Young 15 min, *n* = 5 mice, Aged 15 min, *n* = 4 mice; Young 30 min, *n* = 5 mice, Aged 30 min, *n* = 4 mice; Young 60 min, *n* = 5 mice, Aged 60 min, *n* = 4 mice; Young 120 min, *n* = 3 mice, Aged 120 min, *n* = 5 mice. **k** *Nptx2*^*+*^ cell counts in DG. Young HC, *n* = 5 mice, Aged HC, *n* = 4 mice; Young 15 min, *n* = 4 mice, Aged 15 min, *n* = 5 mice; Young 30 min, *n* = 5 mice, Aged 30 min, *n* = 4 mice; Young 60 min, *n* = 5 mice, Aged 60 min, *n* = 5 mice; Young 120 min, *n* = 5 mice, Aged 120 min, *n* = 4 mice. **l** Fluorescence intensity of *Nptx2* mRNA in *Fos*^*+*^ ensemble in DG. Young HC, *n* = 4 mice, Aged HC, *n* = 4 mice; Young 15 min, *n* = 5 mice, Aged 15 min, *n* = 4 mice; Young 30 min, *n* = 6 mice, Aged 30 min, *n* = 4 mice; Young 60 min, *n* = 5 mice, Aged 60 min, *n* = 4 mice; Young 120 min, *n* = 5 mice, Aged 120 min, *n* = 4 mice. **m** Fluorescence intensity of *Nptx2* mRNA in *Npas4*^*+*^ ensemble in DG. Young HC, *n* = 4 mice, Aged HC, *n* = 4 mice; Young 15 min, *n* = 5 mice, Aged 15 min, *n* = 4 mice; Young 30 min, *n* = 5 mice, Aged 30 min, *n* = 4 mice; Young 60 min, *n* = 5 mice, Aged 60 min, *n* = 4 mice; Young 120 min, *n* = 4 mice, Aged 120 min, *n* = 4 mice. **n** Diagram of AAV injection and experimental scheme to label *F*-RAM and *N*-RAM cells. **o** Representative images of *F*- and *N*-RAM engram cells in DG of young and aged mice. **p** *F*-RAM and *N*-RAM cell counts in DG of young and aged mice. Young *F*-RAM, *n* = 6; Young *N*-RAM, *n* = 7; Aged *F*-RAM, *n* = 5; Aged *N*-RAM, *n* = 4. Data are presented as mean ± SEM; *P < 0.05, **P < 0.01, ***P < 0.001.

Maladaptive changes in expression of functional synaptic proteins, which destabilize neuronal network and impair cognition, represent central hallmarks of physiological brain aging.^8^ NPTXs are considered as biomarkers of synaptic dysfunction and cognitive impairment.^10,19^ To investigate whether *Nptx* expression in DG changes during physiological aging, young (3 months) and aged (18 months) mice were sacrificed 15 min, 30 min, 60 min and 120 min after CFC or under home cage (HC) conditions, single molecule fluorescence in situ hybridization (smFISH) was used to assess their mRNA expression (Fig. 6a–e).

The numbers of *Fos*^*+*^ and *Npas4*^*+*^ cells after CFC were decreased in DG of aged mice, especially at 30 min and 60 min, indicating decreased activation of neurons during learning in aged mice (Fig. 6f, g). *Nptx1* was expressed in all DG granule cells. Although the overall *Nptx1* mRNA fluorescence intensity was not significantly decreased in aged mice (Fig. 6h), the specific expression of *Nptx1* in *Fos*^*+*^ and *Npas4*^*+*^ engram cells was reduced (Fig. 6i, j). NPTX2 was also seen as a neuronal IEG protein,^17^ both the number of *Nptx2*^*+*^ cells and the average fluorescence intensity of *Nptx2* mRNA in *Fos*^*+*^ or *Npas4*^*+*^ engram cells were decreased in aged mice (Fig. 6k–m). Consistent with the decreased number of *Fos*^*+*^ and *Npas4*^*+*^ cells in DG after CFC, the number of cells labeled by the *F*- and *N*-RAM systems were both reduced in DG of aged mice (Fig. 6n–p). These findings indicate the downregulation of *Nptx1* and *Nptx2* in *Fos*^*+*^ and *Npas4*^*+*^ engram cells in aged mice.

### NPTX overexpression in DG engram cells rescued contextual fear memory deficits in aged mice

Ribosome-associated transcripts of *F*-RAM and *N*-RAM ensembles that were activated in HC or by CFC were analyzed (Supplementary information, Fig. S16a). Gene ontology (GO) analysis revealed clusters of differentially expressed genes (DEGs) related to synaptic plasticity and cell communication between *F*-RAM and *N*-RAM ensembles. The difference in these clusters became more significant after CFC (Supplementary information, Fig. S16b). In addition, a small number of DEGs were overlapped between *F*- and *N*-RAM ensembles during aging (Supplementary information, Fig. S16c, d). In *F*-RAM ensemble, DEGs from young and aged mice showed enrichment in pathways regulating neuronal excitability (e.g., membrane potential and potassium ion import across plasma membrane). In contrast, DEGs in *N*-RAM were enriched in the AMPAR complex and others (Supplementary information, Fig. S16e). This differential enrichment suggests that distinct pathways are involved in engram network plasticity related to aging in *F*- and *N*-RAM ensembles.

**Fig. 7:**
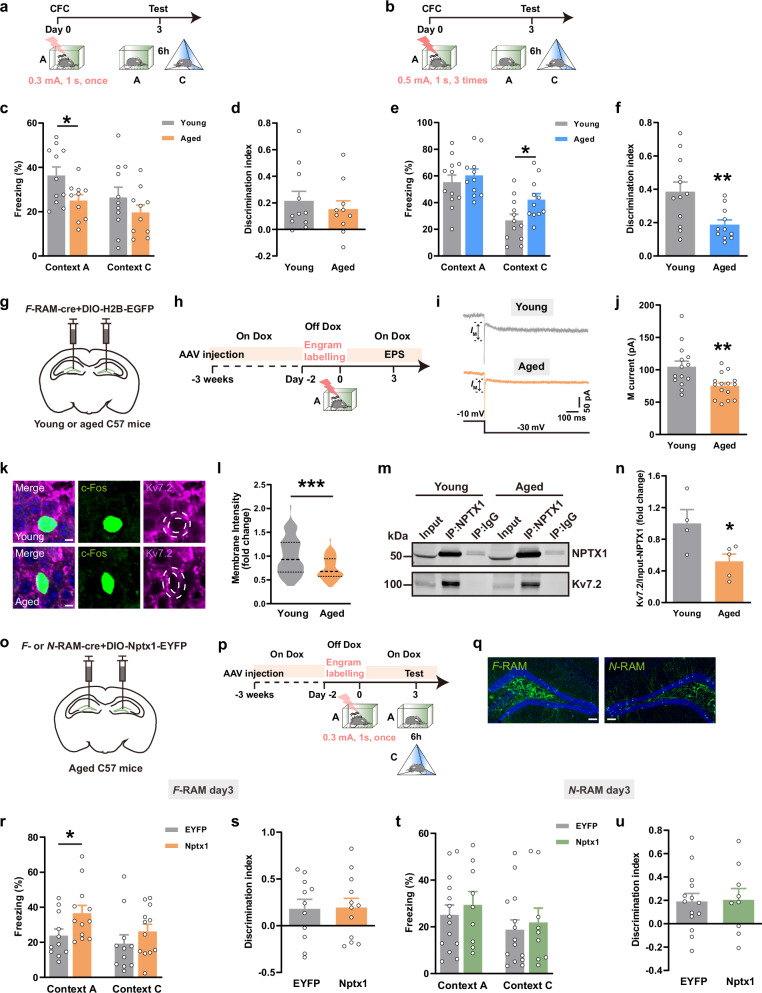
Overexpression of NPTX1 in *F*-RAM ensemble rescued contextual memory imprecision in aged mice. **a**, **b** Experimental scheme of CFC to test memory expression. **c**, **d** Freezing percentage (**c**) and discrimination index (**d**) of young and aged mice. Young, *n* = 11 mice; Aged, *n* = 10 mice. **e**, **f** Freezing percentage (**e**) and discrimination index (**f**) of young and aged mice. Young, *n* = 12 mice; Aged, *n* = 11 mice. **g** Diagram of AAV injection. **h** Experimental scheme for *I*_M_ recording. **i** Representative traces of *I*_M_ currents in *F*-RAM cells of young and aged mice. **j** Average *I*_M_ current amplitudes recorded at –30 mV from young and aged mice. Young, *n* = 14 neurons from 3 mice; Aged, *n* = 15 neurons from 3 mice. **k** Representative confocal images of c-Fos^+^ cells colocalizing with K_v_7.2. Green: c-Fos^+^ engram cells; purple: K_v_7.2; blue: DAPI. Dashed white lines outline cell membrane. Scale bars, 5 μm. **l** Average membrane K_v_7.2 fluorescence intensity of c-Fos^+^ neurons in young and aged mice. Young, *n* = 88 from 6 mice; Aged, *n* = 64 from 6 mice. **m** Co-IP of NPTX1 with K_v_7.2 in DG of young and aged mice. **n** Quantification of immunoprecipitated K_v_7.2 in young and aged mice. Young, *n* = 4 mice; Aged, *n* = 5 mice. **o** Diagram of AAV injection. **p** Experimental scheme of memory retrieval test. **q** Representative expression of NPTX1 in *F*- or *N*-RAM engram cells in DG. Green: *F*-RAM or *N*-RAM ensemble, EYFP or EGFP; blue: DAPI. Scale bars, 100 μm. **r**, **s** Freezing percentage (**r**) and discrimination index (**s**) of EYFP- and Nptx1-overexpressing aged groups (*F*-RAM). EYFP, *n* = 11 mice; Nptx1, *n* = 12 mice. **t**, **u** Freezing percentage (**t**) and discrimination index (**u**) of EYFP- and Nptx1-overexpressing aged groups (*N*-RAM). EYFP, *n* = 14 mice; Nptx1, *n* = 9 mice. Data are presented as mean ± SEM; *P < 0.05, **P < 0.01, ***P < 0.001.

As aging is always accompanied with a decline in memory precision,^37–39^ the expression of contextual fear memory in young and aged mice was further assessed. When exposed to the mild fear conditioning (0.3 mA, 1 s, 1 trial), aged mice froze less in the fear context A (Fig. 7a, c, d), which suggested memory retrieval impairment. However, when exposed to the strong fear conditioning (0.5 mA, 1 s, 3 trials), aged mice froze more in the non-fear context C, indicating memory overgeneralization (Fig. 7b, e, f). These results demonstrate that the precise expression of contextual fear memory is impaired in aged mice.

Decreased *I*_M_ current (Fig. 7g–j), K_v_7.2 membrane expression (Fig. 7k, l) as well as NPTX1–K_v_7.2 interaction in the DG (Fig. 7m, n; Supplementary information, S17a) were observed in aged mice. *AAV-DIO-Nptx1-EYFP* was constructed and delivered with *AAV-F-RAM-Cre* or *AAV-N-RAM-Cre* into DG to overexpress NPTX1 in *F*- or *N*-RAM ensemble (Fig. 7o; Supplementary information, Fig. S17b). Aged mice were subjected to the mild fear conditioning protocol (0.3 mA, 1 s, one shock). Overexpression of NPTX1 in *F*-RAM ensemble, but not in *N*-RAM ensemble, increased the freezing levels of aged mice in the fear context A (Fig. 7o–u; Supplementary information, Fig. S17c, d).

**Fig. 8:**
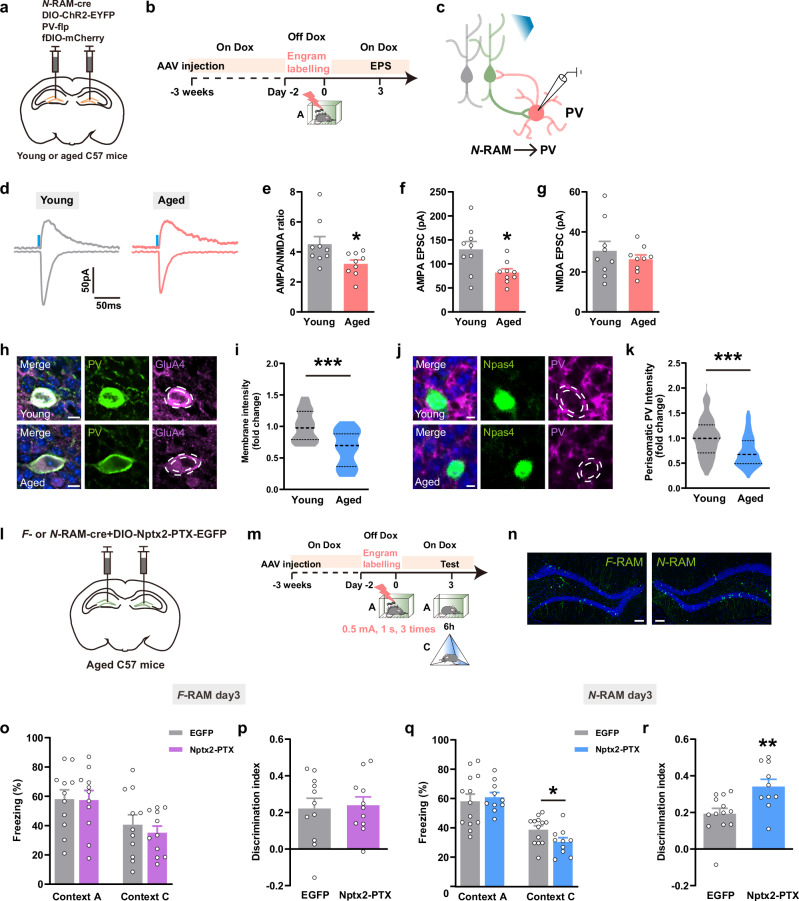
Overexpression of NPTX2 in *N*-RAM ensemble rescued contextual memory overgeneralization. **a** Diagram of AAV injection. **b** Experimental scheme to label *N*-RAM engram ensembles. **c** Diagram of photostimulation and whole-cell patch clamp recordings. **d** Representative traces of opto-evoked AMPA-EPSC and NMDA-EPSC recorded from young and aged mice. **e** Average A/N ratio recorded from young and aged mice. **f**, **g** Average AMPA-EPSC (**f**) and NMDA-EPSC (**g**) amplitudes recorded from young and aged mice. Young, *n* = 9 neurons from 3 mice; Aged, *n* = 9 neurons from 3 mice. **h** Representative confocal images of GluA4 colocalizing with PV^+^ interneurons. Green: PV^+^ interneurons; purple: GluA4. Scale bars, 10 μm. **i** Quantification of GluA4 membrane expression on PV^+^ interneurons in young and aged mice. Young, *n* = 28 neurons from 4 mice; Aged, *n* = 23 neurons from 5 mice. **j** Representative confocal images of PV neurites around Npas4^+^ engram cells. Green: Npas4^+^ engram cells; purple: PV; blue: DAPI. Dashed white lines outline PV neurites. Scale bars, 5 μm. **k** Average fluorescence intensity of PV neurites around Npas4^+^ neurons in young and aged mice. Young, *n* = 160 neurons from 6 mice; Aged, *n* = 94 neurons from 6 mice. **l** Diagram of AAV injection. **m** Experimental scheme of memory retrieval test. **n** Representative expression of NPTX2-PTX in *F*- or *N*-RAM engram cells in DG. Green: *F*-RAM or *N*-RAM ensemble, EYFP or EGFP; blue: DAPI. Scale bars, 100 μm. **o**, **p** Freezing percentage (**o**) and discrimination index (**p**) of EGFP- and Nptx2-PTX-overexpressing aged groups (*F*-RAM). EGFP, *n* = 11 mice; Nptx2-PTX, *n* = 11 mice. **q**, **r** Freezing percentage (**q**) and discrimination index (**r**) of EGFP- and Nptx2-PTX-overexpressing aged groups (*N*-RAM). EGFP, *n* = 13 mice; Nptx2-PTX, *n* = 10 mice. Data are presented as mean ± SEM; *P < 0.05, **P < 0.01, ***P < 0.001.

AAVs encoding *N-RAM-Cre*, *DIO-ChR2-EYFP*, *PV-flp* and *fDIO-mCherry* were delivered into DG of young and aged mice (Fig. 8a–c). Impaired AMPAR-mediated EPSC (Fig. 8d–g), reduced GluA4 membrane expression in PV^+^ interneurons (Fig. 8h, i) and decreased PV^+^ fluorescence intensity surrounding Npas4^+^ engram cells (Fig. 8j, k) were observed in aged mice. These results are consistent with the phenotype of *Nptx2* depletion in *N*-RAM ensemble of young mice (Fig. 3). Overexpression of the AMPAR binding domain of NPTX2 (NPTX2-PTX)^22^ in *N*-RAM ensemble, but not in *F*-RAM ensemble, rescued the overgeneralization of fear memory in aged mice (Fig. 8l–r; Supplementary information, Fig. S17e, f). However, in young mice, overexpression of NPTX1 or NPTX2-PTX in *F*- and *N*-RAM ensembles failed to alter freezing levels in context A or C (Supplementary information, Fig. S18). These data confirm that the hyperactivity of DG engram network caused by NPTX downregulation underlies aging-associated deficits in contextual fear memory, and re-stabilizing the NPTX1-dependent MEC-DG excitatory circuit and NPTX2-dependent DG PV^+^ interneuron inhibitory circuit is able to repair contextual fear memory deficits in aged mice.

## Discussion

**Fig. 9:**
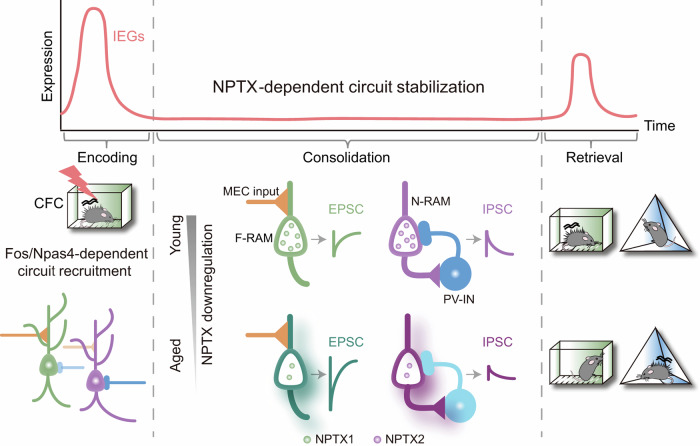
Working model illustrating main findings. During contextual fear memory learning (encoding), *Fos* transcription- and *Npas4* transcription-dependent engram ensembles (*F*-RAM and *N*-RAM) are activated to preferentially recruit excitatory and inhibitory inputs. During memory consolidation, NPTX1 functions in *F*-RAM ensemble to restrict the response of this ensemble to MEC excitatory inputs, and NPTX2 functions in *N*-RAM ensemble to facilitate the recruitment of inhibitory inputs from PV^+^ interneurons. NPTX1 and NPTX2 cooperate to suppress engram network hyperactivity, thereby ensuring precise memory expression during retrieval. Downregulation of NPTX1 and NPTX2 in engram cells of aged mice contributes to destabilization of the engram network, resulting in contextual fear memory deficits.

During learning, neuronal ensembles that recruit excitatory and inhibitory circuits are activated by changes in synaptic connectivity. However, it is unclear how the recruited engram network maintains its stability. The consolidation of newly acquired memories requires gene transcription and translation. The transcription of IEGs (beyond their mRNA expression) activates downstream targets that maintain memory stability. Our study identified NPTXs as the molecular substrates that prevent DG engram network hyperactivity during memory consolidation and maintain the precision of contextual fear memory (Fig. 9). Although NPTX upregulation following CFC occurred in cells expressing endogenous *Fos* and *Npas4* (Fig. 6), specific behavioral outputs were observed when *Nptxs* were knocked out in *F*- or *N*-RAM ensemble with a delay (24–72 h after CFC). These ensembles are tagged by activated Fos and Npas4 proteins, which drive meaningful transcriptional outputs. ChIP-qPCR results (Fig. 1h–j) showed that c-Fos was preferentially enriched in the TSS region of *Nptx1*, whereas Npas4 was significantly enriched in the TSS region of *Nptx2* following CFC. Previous study^21^ and our data (Supplementary information, Fig. S1) demonstrated anatomical segregation between *F*- and *N*-RAM ensembles. The functional specificity of NPTXs should be placed into specific engram circuits to be manifested.

NPTX1 plays a critical role in stabilizing the excitatory synaptic connection between MEC and DG *F-*RAM cells via facilitating K_v_7.2 membrane expression-dependent inhibition of neuronal hyperexcitability to promote contextual fear memory retrieval. Our data demonstrated that NPTX1 interacts with the potassium channel K_v_7.2, although this interaction may not be direct and requires other auxiliary proteins such as syntaxin.^40^ This finding provides a potential mechanism by which NPTX1 regulates neuronal excitability independently of its classical role in glutamate signaling. As a secretory protein, NPTX1 could form complexes with K_v_7.2 and facilitate K_v_7.2 membrane trafficking. The hyperexcitability observed in *Nptx1* cKO mice may result from the impaired trafficking and membrane enrichment of K_v_7.2. The contextual fear memory impairment caused by *Nptx1* depletion in *F*-RAM ensemble contrasted with Sun et al.’s^21^ finding that optogenetic inhibition of MEC-DG circuit suppressed fear memory generalization. This is probably because optogenetic manipulation was transient, while *Nptx1* depletion mimics the sustained neuronal activation during memory consolidation. This persistent hyperexcitability in engram cells decreased the signal-to-noise ratio of excitatory transmission from MEC to *F-*RAM ensemble during memory retrieval. Furthermore, accumulating evidence indicates that MEC inputs to DG regulate memory retrieval.^41,42^

NPTX2 stabilizes the inhibitory synaptic connection between DG local PV^+^ interneurons and *N*-RAM ensemble to suppress overgeneralization of contextual fear memory. This result was consistent with Sun et al.’s report that *N*-RAM ensemble contributed to memory discrimination.^21^ Their finding confirmed the critical role of CCK^+^ interneurons, while our finding illustrated the importance of NPTX2-dependent PV^+^ interneuron plasticity. We hypothesize that *N*-RAM ensemble-dependent fear memory discrimination requires the inhibitory inputs from both CCK^+^ and PV^+^ interneurons, as they were found to exert complementary roles in recruiting perisomatic inhibition.^43,44^ Notably, NPTX2 is specifically involved in regulating the functional connection between PV^+^ interneurons and *N-*RAM engram cells. NPTX2 was identified as a downstream effector of Npas4.^45,46^ This accounted for the coincidence that both *Nptx2* knockout and *Npas4* depletion^47^ impaired perisomatic inhibition. As a neuronal IEG, Npas4 upregulates perisomatic inhibitory synapses when activated^46,48^ to scale down the level of network activity, but the specific mechanism remains to be elucidated. Our study provides the downstream molecular mechanism for Npas4 transcription-dependent mediation of contextual fear memory precision. The functional specificity of NPTX2 in *N-*RAM ensemble (but not in *F-*RAM ensemble) for contextual fear memory discrimination was further supported by Ee-Lynn et al.’s study that knockdown of *Nptx2* in *Fos*^*+*^ ensemble had no effect on PV^+^ interneuron-mediated perisomatic inhibition.^49^

Memory precision changes dynamically across the lifespan. Young individuals initially form only gist-like memories, which gradually develop into precise episodic memories as the brain matures. Subsequently, the ability to retrieve specific information declines with age. Understanding the maladaptive changes in engram network stability during aging may provide insights into combating aging-induced cognitive deficits. NPTXs are classic synaptic proteins that are also prognostic biomarkers in cognitive and mental disorders, such as AD and schizophrenia.^17,18^ *Nptx2* was also seen as a neuronal IEG. Our smFISH results showed that the total number of *Nptx2*^*+*^ cells and the expression of *Nptx2* in engram cells were both downregulated in DG during aging, whereas the overall downregulation of *Nptx1* was not observed in aged mice, indicating that NPTX2 is more sensitive to aging. That is to say, during aging, *Nptx2* downregulation-induced disruption of local perisomatic inhibition in DG engram precedes *Nptx1* downregulation-induced disruption of long-range MEC-DG excitatory projection; this probably explains why generalization always emerges before amnesia in aged individuals. The finding of Ramsaran et al. showed that extracellular perineuronal net-dependent functional maturation of PV^+^ interneurons promotes sparse engram formation and memory precision during brain development.^50^ Our study elucidated that engram network hyperactivity, caused by NPTX downregulation, contributed to aging-related contextual fear memory deficits and provided potential synaptic and molecular strategies to mitigate different phenotypes of aging-related contextual fear memory imprecision, such as amnesia and overgeneralization.

Another interesting finding in our study is that the number of *Fos*^*+*^ and *Npas4*^*+*^ cells, as well as the number of *F*- and *N*-RAM tagged cells, was significantly reduced in aged brain (Fig. 6), which was in accordance with previous studies.^51–53^ Decreased activation and expression of IEGs in aged animals may accelerate cell senescence and increase the susceptibility to neurodegeneration, thereby impairing cognition and decreasing lifespan.^47,54^ In addition, memory impairments were found in *c-Fos* or *Npas4* KO mice,^55–57^ indicating that Fos- and Npas4-dependent transcriptional activation is essential for neuronal network plasticity and cognitive maintenance. The disruption of this process may also contribute to aging-related memory deficits.

However, there are some limitations in our study. (1) Npas4 can regulate the transcription of Fos or itself,^48^ indicating the complex regulatory network between these IEGs. Whether Fos and Npas4 competitively bind to downstream genes, or whether *Npas4*^*+*^ and *Fos*^*+*^ neurons can mutually transform, remains to be investigated. (2) Unlike *Nptx2*, *Nptx1* is consistently expressed in the brain, it is unknown whether there exists a causal relationship between NPTX1-dependent inhibition of neuronal hyperexcitability and engram formation. (3) It cannot be denied that memory encoding and consolidation involve multiple interacting molecular and circuit-level processes beyond NPTX regulation. The downregulation of NPTX1 and NPTX2 in specific DG circuits that encode the contextual fear memory could represent a key mechanism underlying contextual fear memory deficits during aging. According to our RNA-seq data (Supplementary information, Fig. S16), other molecules that contribute to Fos- and Npas4-dependent circuit plasticity could be further investigated. (4) The present study exclusively relying on the CFC paradigm as a measure of memory precision and generalization is limiting, as it does not fully capture the complexity of memory processes.

## Materials and Methods

### Animals

All animals used in this study were listed as follows: adult C57BL/6J male mice (3 months) from the SLAC Laboratory Animal Company (Shanghai, China), aged C57BL/6J male mice (16–18 months) from the Aniphe Biolaboratory Inc. (Nanjing, China) and *PV-Flpe* (021191) mice from the Jackson Laboratory (CA, USA). CRISPR-Cas9-mediated construction of *Nptx1*^*fl/fl*^, *Nptx2*^*fl/fl*^ conditional knockout (*Nptx1* cKO, *Nptx2* cKO) mice targeting exon 3 and exon 2, respectively, were generated by the Biocytogen Co. (Beijing, China) and *Npas4-CreER*^*T2*^ mice were generated by Shanghai Model Organisms Center (Shanghai, China). *Npas4-CreER*^*T2*^ and *PV-Flpe* mice were bred to C57BL/6J mice for more than six generations, *Nptx1*^*fl/fl*^, *Nptx2*^*fl/fl*^ mice and their respective WT littermates were obtained from self-crossing of *Nptx1*^*fl/+*^, *Nptx2*^*fl/+*^ (heterozygous) mice. Genotypes were determined by PCR of mouse toe DNA samples. The primers for genotyping PCR were provided in Supplementary information, Table S1. Male offsprings at 8–12 weeks of age were used in the following experiments, which were randomly assigned to groups. All mice were housed on a 12 h light/dark cycle (light on from 8 a.m. to 8 p.m.) with access to food and water ad libitum. All experiment procedures were strictly in accordance with the National Institutes of Health Guide for the Care and Use of Laboratory Animals, and were approved by Animal Care and Use Committee of the animal facility at Fudan University.

### Viral vectors

AAV-Fos-RAM-d2tTA-TRE-mKate2 and AAV-Npas4-RAM-d2tTA-TRE-mKate2 plasmids were kind gifts from Prof. Yingxi Lin (The University of Texas Southwestern Medical Center, TX, USA). To generate AAV-Fos-RAM-d2tTA-TRE-Cre and AAV-Npas4-RAM-d2tTA-TRE-Cre plasmids, *mKate2* in AAV-Fos-RAM-d2tTA-TRE-mKate2 and AAV-Npas4-RAM-d2tTA-TRE-mKate2 plasmids was replaced with the *Cre* sequence obtained by PCR from pAAV-Cre-GFP (Addgene: 68544). pAAV-CMV-bGlobin-Flex-EGFP-MIR30-Scramble-shRNA, pAAV-CMV-bGlobin-Flex-EGFP-MIR30-Nptx2-shRNA and pAAV-CMV-DIO-Nptx2-PTX-P2A-EGFP were used in our previous paper.^22^ To generate pAAV-Ef1α-DIO-Nptx1-T2A-EYFP, the coding sequence for mouse *Nptx1* with *T2A* was generated by Azenta US, Inc. (Suzhou, China) and subcloned into pAAV-Ef1α-DIO-EYFP (Addgene: 27056). Adeno-associated viruses (AAVs) described above were packaged by OBiO Technology Co., Ltd. (Shanghai, China) into serotype 9. *AAV*_*9*_*-Ef1α-Flex-NBL10* (69971) and *AAV*_*9*_*-Ef1α-DIO-ChR2-EYFP* (S0199-9-H50) were purchased from Taitool Bioscience Co., Ltd. (Shanghai, China). *AAV*_*9*_*-Ef1α-DIO-H2B-EGFP* (PT-0258), *AAV*_*9*_*-Ef1α-DIO-EYFP* (PT-0012), *AAV*_*9*_*-CaMKIIα-ChR2-mCherry* (PT-0297), *AAV*_*9*_*-VGAT2-Flp* (PT-2501, vector backbone from BrainVTA), *AAV*_*9*_*-CCK-fDIO-mCherry* (PT-8508, vector backbone from Addgene: 114471), *AAV*_*8*_*-Ef1α-DIO-TVA-H2B-EGFP* (PT-0021), *AAV*_*8*_*-Ef1α-DIO-RVG* (PT-0023) and *RV-ENVA-ΔG-dsRed* (R01002) were purchased from BrainVTA Co., Ltd. (Wuhan, China). *AAV*_*9*_*-PV-Flp* (BC-0430, vector backbone from Addgene: 22914), *AAV*_*9*_*-SST-Flp* (BC-0429, vector backbone from Addgene: 22913), *AAV*_*9*_*-Ef1α-fDIO-ChR2-mCherry* (BC-0113), *AAV*_*9*_*-Ef1α-fDIO-mCherry* (BC-0193) and *AAV*_*9*_*-Ef1α-fDIO-hM3Dq-mCherry* (BC-0495) were purchased from Brain Case Co., Ltd. (Shenzhen, China).

### Stereotaxic surgery

Mice were anesthetized with 2% isoflurane and placed in a stereotaxic instrument (RWD Life Science, Shenzhen, China). Microinjections were performed using 33-gauge needles connected to a 10 μL microsyringe (Hamilton, Bonaduz, Switzerland), which were under the control of a UMP3 ultra micropump (World Precision Instruments, FL, USA). The coordinates relative to bregma were listed as follows: anterior-posterior (AP) – 1.9 mm; medial-lateral (ML) ± 1.1 mm; dorsal-ventral (DV) – 2.1 mm for DG and AP – 4.7 mm; ML ± 3.2 mm; DV – 4.5 mm for MEC. The needle was slowly lowered to the target site and remained for at least 3 min after injection. The injection volume per site was 0.3 μL in DG and 0.4 μL in MEC. The final titer of all the AAVs used for infections were at least 1 × 10^12 ^V.G./mL except for *AAV-SST-Flp*, *AAV-PV-Flp*, *AAV-VGAT2-Flp*, *AAV-Fos-RAM-d2tTA-TRE-Cre* and *AAV-Npas4-RAM-d2tTA-TRE-Cre*, which were 1:1000 diluted. For RV infections, the final titer used was 1 × 10^8^ IFU/mL. After surgery, all mice were given at least 3 weeks to recover before behavioral experiments or electrophysiological recordings, and the efficiency of virus infection was verified by immunostaining. Only the mice with virus infection in correct places were chosen for further analysis.

### Engram labeling

To label engram cells, *Fos-RAM-d2tTA-TRE* (*F*-RAM) and *Npas4-RAM-d2tTA-TRE* (*N-*RAM) systems, whose gene expression was under the control of the tetracycline responsive element (TRE), were used through AAV infusion. All mice were kept on Dox (Huamaike Bio, 360304) containing food (40 mg/kg) one day before virus injection. 48 h (day –2) before engram labeling, mice were fed with regular food instead of Dox diet. Then CFC or novel context exposure (context N) was performed on day 0 to label *F-*RAM or *N-*RAM engram cells in DG, after labeling, mice were put on Dox-containing food again immediately. Mice were fed at least 3 days to allow sufficient protein expression before subsequent experiments.

### RV tracing of inputs

To study the respective monosynaptic inputs of *F-*RAM or *N-*RAM ensembles in DG, RV trans-synaptic tracing experiment was performed. Mice were infected with *AAV*_*9*_*-Fos-RAM-d2tTA-TRE-Cre* or *AAV*_*9*_*-Npas4-RAM-d2tTA-TRE-Cre*, AAV helper (*AAV*_*8*_*-Ef1α-DIO-TVA-H2B-EGFP* and *AAV*_*8*_*-Ef1α-DIO-RVG*). On day 3 after engram labeling, RV (*RV-ENVA-ΔG-dsRed*) was injected into DG at the same coordinates, and then mice were housed in the BSL2 facility for 1 week to allow rabies spread and dsRed expression before perfusion (day 10). For RV tracing analysis, consecutive brain slices from AP + 2.8 mm to AP – 5.0 mm (50 μm thickness) selected from every fifth slice were collected. DsRed^+^ cells were manually counted by an experimenter who was blind to the experimental condition. The percentage of rabies-labeled inputs was calculated as follows: dsRed^+^ cells in each brain region/total dsRed^+^ cells per mouse.

### CFC

Before CFC was performed, mice were handled daily in a holding room for 3 days. For experiments with Dox-dependent ensemble labeling, on the third handling day, the Dox diet was replaced with regular food (off Dox) and CFC assay was typically carried out 48 h after the last handling session. CFC was performed in the conditioning chamber (Med-Associates, St. Albans, VT, USA) and the procedure was composed of conditioning and test sessions.

On the conditioning day (day 0), mice were firstly transported into the holding room and allowed to habituate for at least 30 min, then transported into the behavioral room and placed into context A, a square plexiglass observational chamber with stainless steel bars connected to a shock generator on the floor for conditioning. Two individual conditioning protocols were used in our study, for mice that conditioned to the 360 s protocol, three foot shocks (0.5 mA, 1 s each) at 180 s, 240 s and 300 s were given and mice were taken out 60 s after termination of the third foot shock, for mice that conditioned to the 180 s protocol, one single foot shock (0.3 mA, 1 s) at 120 s was given and mice were taken out 60 s after termination of the foot shock.

The test session was carried out on day 3. Mice were put back into fear context A for 180 s, and were placed into a non-fear context (context C, a triangular chamber with white, smooth plastic floor and black cover) for 180 s 6 h later. For immunohistochemistry, mice were tested for memory expression in only one context, either context A or C, and sacrificed 1 h later.

The freezing percentage was automatically analyzed by software (Med-Associates) with freezing defined as absence of movement for at least 1 s. The discrimination index was calculated as follows: (the freezing percentage in context A – the freezing percentage in context C)/(the freezing percentage in context A + the freezing percentage in context C).

### NOR

Mice were allowed to explore two identical objects (object A) presented in two different places for 10 min, and after 1 h delay, mice were put back to the chamber for 5 min with one object replaced by a novel one (object B).

### NPR

Mice were allowed to explore two identical objects (object C) presented in two different places for 10 min, and after 1 h delay, mice were put back to the chamber for 5 min with one object moved to another place. The time spent exploring each object in NOR and NPR memory tasks were analyzed by an experimenter who was blind to the project and the discrimination ratio was calculated as follows: (time exploring the novel/displaced objects – time exploring the familiar/stationary objects)/time exploring both objects.

### Open field test

Spontaneous locomotor activity was carried out as we previously reported.^58^ In short, mice were placed in the center of an open arena (40 cm × 40 cm) at the beginning of the test, and were allowed to freely explore the arena for 20 min. Distance traveled in the arena and time in center zone were quantified using a TopScan automated detection system (CleverSys, Reston, VA, USA).

### O-maze test

The O-maze was 70 cm in diameter and 70 cm high off the ground, and consisted of two open arms (7 cm wide) without walls, two closed arms that were enclosed by vertical walls. Mice were gently placed into the open arms, and their behaviors were recorded for 6 min via a TopScan automated detection system (CleverSys, Reston, VA, USA) located above the maze.

### Tail suspension test (TST)

Mice were suspended 20 cm above a solid surface by the use of adhesive tape applied to the tail, and their behaviors were recorded for 6 min. Immobility time was defined as absence of struggling for at least 1 s and latency to immobility was defined as the time from the beginning of TST to mouse’s first absence of struggling for at least 1 s, which were manually analyzed by an experimenter who was blind to the experimental condition.

### Drug injections

CNO (Sigma, C0832) was dissolved in saline, and i.p. injected at a concentration of 1 mg/kg 30 min before memory retrieval. The KCNQ2/3 activator, retigabine (Supelco, 90221) was diluted in 5% DMSO (Sigma, 276855) and administered i.p. at 1 mg/kg 30 min before memory retrieval with 5% DMSO as control. 4-OHT (Sigma, H6278) was dissolved in ethanol at 20 mg/mL and stored at –20 °C. The ethanol was evaporated at 95 °C and corn oil (Thermo Fisher Scientific, C40543) was added to give a final concentration of 10 mg/mL 4-OHT, which was i.p. injected 2 h before memory conditioning with corn oil as control.

### Whole-cell patch-clamp recording in brain slices

Mice were subjected to electrophysiological recordings on day 3 following CFC. Preparation of living acute brain slices (300 μm) for analyzing engram synaptic connections and engram intrinsic excitability was performed as previously described^59^ only with minor modifications. Briefly, mice’s brains were cut on a vibratome (Thermo Fisher Scientific, MA, USA) in carbogenated (95% O_2_, 5% CO_2_) ice-cold cutting solution containing: 93 mM NMDG, 2.5 mM KCl, 1.25 mM NaH_2_PO_4_, 30 mM NaHCO_3_, 20 mM HEPES, 25 mM glucose, 2 mM thiourea, 5 mM Na-ascorbate, 3 mM Na-pyruvate, 0.5 mM CaCl_2_ and 10 mM MgCl_2_, 300–310 mOsm, pH adjusted to 7.3 with HCl. After initial recovery at 32 °C for 10 min, slices were transferred to carbogenated HEPES holding ACSF (92 mM NaCl, 2.5 mM KCl, 1.25 mM NaH_2_PO_4_, 30 mM NaHCO_3_, 20 mM HEPES, 25 mM glucose, 2 mM thiourea, 5 mM Na-ascorbate, 3 mM Na-pyruvate, 2 mM CaCl_2_ and 2 mM MgCl_2_, 300–310 mOsm, pH 7.4) and incubated for over 45 min before recording. Whole-cell patch-clamp recordings were performed in carbogenated recording ACSF (119 mM NaCl, 2.5 mM KCl, 1.25 mM NaH_2_PO_4_, 24 mM NaHCO_3_, 12.5 mM glucose, 2 mM CaCl_2_ and 2 mM MgCl_2_ (300–310 mOsm, pH 7.3–7.4) at a rate of 1.5 mL/min (30–32 °C) with an EPC-10 amplifier and Pulse v8.78 software (HEKA Elektronik, Lambrecht/Pfalz, Germany). Recorded neurons were identified visually by location, morphology, size and fluorescence, and recordings were performed using borosilicate glass pipettes (5–7 mΩ tip resistance).

To record A/N ratios, neurons were voltage-clamped at –70 mV to record AMPAR-mediated EPSCs and at +40 mV to record dual-component EPSCs containing NMDA receptor (NMDAR) EPSCs. To calculate the A/N ratios, the peak current of the AMPA EPSC at –70 mV was compared with the value of the NMDA EPSC after stimulation start time 50 ms at +40 mV. Intracellular solution used was: 127.5 mM cesium methanesulfonate, 7.5 mM CsCl, 10 mM HEPES, 2.5 mM MgCl_2_, 4 mM Mg-ATP, 0.4 mM Na_3_-GTP, 10 mM sodium phosphocreatine, 0.6 mM EGTA (290–300 mOsm, pH 7.2).

To record light-evoked EPSCs (oEPSC), a TTL-driven light-emitting diode (Lumen Dynamics) was used to generate photostimulation consisting of a single wide-field blue flash (470 nm, 1 ms duration) for photostimulation of ChR2-expressing terminals. The laser intensity was measured at the focal plane of the slice when delivered through the 40× water-immersion objective lens (Nikon, Japan). Slices containing MEC ChR2-expressing terminals were chosen for oEPSC recording in the presence of 1 μM Tetrodotoxin (TTX, Tocris, 1078), 100 μM 4-aminopyridine (4-AP, Sigma, 275875) and 100 μM picrotoxin (PTX, Tocris, 1128), neurons were voltage-clamped at –70 mV and the stimulus intensity was 0.1 mW at 470 nm. To record PPR and A/N ratio, DG slices containing ChR2-expressing neurons were chosen to perform patch-clamp recordings in the presence of 100 μM PTX and the stimulus intensity was 0.1 mW at 470 nm. The AMPAR-mediated EPSCs were evoked by paired photostimulation of 50-ms interval for 10 consecutive traces, and PPR was determined as the peak amplitude ratio of the second to the first EPSC. To record light-evoked IPSCs (oIPSC) and PPR, neurons were clamped at +10 mV in the presence of 1 μM TTX, 100 μM 4-AP, 20 μM CNQX (Sigma, C127) and 50 μM D-AP5 (Tocris, 0106) and the stimulus duration and intensity were 1 ms, 0.2 mW at 470 nm. The recording protocols and intracellular solution used were the same as above.

To record AP, current-clamp was used and membrane potentials were measured in response to intracellular injection of step currents (1000 ms duration, magnitudes ranging from –150 pA to 250 pA in steps of 10 pA) with the addition of 20 μM CNQX, 50 μM D-AP5 and 100 μM PTX into ACSF. Intracellular solution used was: 135 mM potassium-gluconate, 4 mM KCl, 2 mM NaCl, 10 mM HEPES, 4 mM EGTA, 4 mM Mg-ATP, 0.3 mM Na3-GTP, 10 mM sodium phosphocreatine (280–290 mOsm, pH 7.3).

To record and isolate *I*_M_, intracellular solution used was the same as above. 0.2 mM CdCl_2_ (Sigma, 202908), 1 μM TTX, 10 μM ZD7288 (Tocris, 1000), and 4 mM 4-AP to block voltage-dependent Ca_v_, Na_v_, HCN and K_v_1 channels, and 20 μM CNQX, 50 μM D-AP5 and 100 μM PTX to block synaptic activity were added into ACSF. To isolate *I*_M_, the following protocol as previously reported^60^ with minor modifications was applied to the recording cells: (1) a 1 s step from the holding potential (–70 mV) to –10 mV was applied to activate *I*_M_ while inactivating most other voltage-gated currents, (2) a 1 s step from –60 mV to –10 mV with 10 mV increments to elicit *I*_M_ tail current, and (3) a 1 s step to –10 mV, before returning to –70 mV.

The signals were acquired at 10 kHz and filtered at 2 kHz. The series resistance was < 30 mΩ. Data were analyzed with Mini Analysis Program (Synaptosoft, Fort Lee, NJ, USA) or pCLAMP10.7 (Molecular Devices, San Jose, CA, USA) by an experimenter who was blind to the experimental condition.

### Ribosome-associated mRNA purification/RiboTag

DG tissues from the mice injected with *AAV*_*9*_*-F/N-RAM-Cre* and *AAV*_*9*_*-Flex-NBL10* (Cre-dependent expression of N-terminus of ribosomal subunit protein Rpl10a (NBL10)^61,62^) following fear conditioning (day 3) were quickly isolated and used for enrichment of ribosome-associated transcripts as described previously.^22^ The brain tissue (DG) was homogenized in supplemented hybridization buffer (HB-S) containing dithiothreitol (Sigma, D9760), cycloheximide (Cayman, 14126), heparin (Tocris, 2812), protease inhibitors (Roche, 04693116001), and RNase inhibitor (Vazyme, R301). The supernatant was incubated with anti-hemagglutinin (HA) antibody (Sigma, H6908) and Dynabeads Protein G (Invitrogen, 10003D) for 12 h. Purified mRNA was eluted from the Dynabeads using TRIzol^TM^ LS (Invitrogen, 10296010). An Agilent RNA 6000 Pico Kit (Agilent, 5067-1513) and an Agilent 2100 bioanalyzer were used to evaluate the quality of purified mRNA. Purified mRNA samples with RNA integrity number < 7 were discarded.

### Sequence processing and data analysis

The library was prepared with VAHTSTM mRNA-seq V3 Library Prep Kit for Illumina (Vazyme, NR611) and sequenced on a HiSeq 4000 (Illumina) by Novogene Technology Co., Ltd. (Beijing, China). Raw reads were cleaned with FASTX-toolkit to remove adapter contamination and low-quality reads (quality score < 28). The clipped reads were aligned to mouse reference sequence (GRCm38/mm10) using HISAT2.^63^ Cuffdiff-generated FPKM count matrix was used for subsequent analysis. Significance was drawn with analysis of variance (ANOVA) and clustered using Shannon entropy-based method.^64^ Genes with > 1.5-fold expression changes and significant difference (*P* < 0.05) were selected for further analysis.

### smFISH

Fixed-frozen brain tissues were sliced into 10-μm coronal sections and baked at 60 °C for 30 min. Then slices were incubated with H_2_O_2_ for 10 min at room temperature (RT), and target retrieval and protease III incubation were performed using RNAscope® 2.5 Universal Pretreatment Reagents (Advanced Cell Diagnostics, 322000, 322381). smFISH probes for all genes examined: *Nptx1* (505421), *Nptx2* (316901), *Fos* (316921) and *Npas4* (423431) were hybridized for 2 h. After hybridization, RNAscope® Multiplex Fluorescent Detection Kit v2 (Advanced Cell Diagnostics, 323110) was used to amplify signals. Images were acquired by using the Nikon A1 confocal microscope (Tokyo, Japan). Regions of interest were circled and the intensity within the regions was analyzed by Image-Pro Plus 6.0 (Media Cybernetics, Rockville, MD, USA). For cell counting analysis, consecutive brain slices from AP – 1.2 mm to AP – 2.0 mm (10 μm thickness) were selected from every tenth slice, 8 slices per mouse were collected, and the numbers of mice were included in Supplementary information, Table S2.

### Immunohistochemistry

Mice were perfused transcardially with ice-cold saline followed by 4% paraformaldehyde (PFA, dissolved in 0.1 M PBS). The brains were removed and fixed in 4% PFA overnight. Then the brains were dehydrated in 30% sucrose solutions at 4 °C for 72 h before being sliced into 30-μm or 40-μm coronal sections (selected from every fifth slice). Slices were incubated with primary antibodies in blocking solution containing 0.3% Triton X-100 overnight at 4 °C. Slices were washed with 0.1 M PBS, and then incubated with secondary antibody at RT for 1.5 h. After being washed in PBS, slices were mounted in anti-quenching mounting medium (Southern Biotech, 0100). Primary antibodies used were: anti-NPTX1 (1:400, Alomone Labs, ANR-191), anti-NPTX2 (1:500, Proteintech, 10889-1-AP), anti-Fos (1:500, Synaptic Systems, 226017), anti-parvalbumin (1:100, Thermo Fisher Scientific, PA1-933), anti-PV (1:500, Oasis, OB-PGP005-01), anti-GluA4 (1:500, Merck, AB1508), anti-Somatostatin (SST, 1:200, Oasis, OB-PRB111-01), anti-ChAT (1:100, Merck, AB143), anti-VGluT1 (1:500, Synaptic Systems, 135302), anti-GAD67 (1:500, Merck, MAB5406), anti-cholecystokinin (CCK, 1:1000, Merck, C2581) and anti-KCNQ2 (1:100, Alomone Labs, APC-050). Secondary antibodies used were: goat anti-rat 488 (1:1000, Jackson Immuno Research, 112-545-167), goat anti-rabbit 488 (1:1000, Jackson Immuno Research, 111-545-144), donkey anti-guinea pig 488 (1:1000, Jackson Immuno Research, 706-545-148), goat anti-rabbit Cy3 (1:1000, Jackson Immuno Research, 111-165-144), donkey anti-rat Cy3 (1:1000, Jackson Immuno Research, 712-165-153) and donkey anti-rat 647 (1:500, Jackson Immuno Research, 712-605-150). K_v_7.2 and GluA4 membrane expression analysis were adapted from one of the latest researches from our lab.^65^ Images were acquired using a Nikon-A1 confocal microscope (Tokyo, Japan) with a 20× objective lens or a 60× objective oil lens. Data were analyzed blindly to the group using Image-Pro Plus 6.0 and ImageJ (Fiji). For cell counting analysis, consecutive brain slices from AP – 1.2 mm to AP – 2.0 mm (40 μm thickness) selected from every fifth slice, 4 slices per mouse were collected, and the numbers of mice were included in Supplementary information, Table S2.

### ChIP

Adult C57BL/6J male mice were subjected to CFC (0.5 mA, 1 s, 3 times), and 1 h later, DG tissues were quickly isolated. ChIP was performed using the ChIP Assay Kit (Beyotime, P2078) according to the manufacturer’s instructions with modifications. Briefly, the samples were minced and crosslinked with 1.5% formaldehyde at RT for 12 min, followed by quenching with glycine for 5 min. The samples were then homogenized and chromatin was sheared by incubating with nuclei lysis buffer, followed by sonication (100 W, 30 s on/off cycles; 10 cycles) to disrupt the nuclear membrane. For immunoprecipitation, the diluted chromatin was incubated overnight at 4 °C with 4 μg anti-IgG (Cell Signaling Technology, 2729S), anti-Fos (Cell Signaling Technology, 2250) or anti-Npas4 antibody (Activity Signaling, AS-AB18A-100) with continuous rotation, followed by an additional 1 h incubation with 30 μL Protein A + G Agarose. DNA was then eluted and purified and subjected to qPCR. ChIP-qPCR results were calculated as a percentage of input DNA.

### Reverse transcription-quantitative PCR

Reverse transcription was completed using HiScript II 1st Strand cDNA Synthesis Kit (Vazyme, R212). The cDNA was subjected to qPCR using ChamQ Universal SYBR qPCR Master Mix (Vazyme, Q711) and Eppendorf Mastercycler PCR System. The mRNA expression of *Nptx1* and *Nptx2* was normalized to that of the internal control *Gapdh*. All primers used for PCR amplification were listed in Supplementary information, Table S1.

### Co-IP and western blotting

The DG tissues of adult C57BL/6J male mice were rapidly extracted and homogenized in radio-immunoprecipitation assay (RIPA) buffer containing protease and phosphatase inhibitors for 30 min. The protein lysate concentration was determined using bicinchoninic acid kit. Protein samples were incubated with 3 µg anti-NPTX1 antibody (Santa Cruz Biotechnology, sc-374199) or anti-IgG antibody (Cell Signaling Technology, 5415S) for 4 h at 4 °C. Pierce Protein A/G magnetic beads (Thermo Fisher Scientific, 88803) were added to each sample and the mixture was rotated at 4 °C overnight. The attached protein was then eluted and used for further assay. Before western blotting assay, 50 µg protein samples were boiled for 6 min at 85 °C, and were separated by SDS-PAGE under 120 V for 1.5 h. Proteins were then transferred to the polyvinylidene fluoride (PVDF) membrane at 100 mV for 100 min. The membrane was firstly washed with Tris-buffered saline with Tween 20 (TBST), followed by blocking in 5% skimmed milk for 2 h. The membrane was then incubated with the following primary antibodies anti-NPTX1 (1:100, Santa Cruz Biotechnology, sc-374199) and anti-KCNQ2 (1:500, Abcam, ab22897) at 4 °C overnight. On the next day, the membrane was washed with TBST and incubated with IRDye 700DX- or 800DX-conjugated anti-rabbit or anti-mouse IgG (1:50,000, Rockland Immunochemicals Inc.) for 2 h at RT. Protein bands were visualized using Odyssey (LI-COR Biosciences). The immunoblots were analyzed with Image J.

### Statistics

All data were presented as mean ± SEM. Sample sizes were based on our previous research.^22,58,66^ Statistical analyses were performed by SPSS 20.0 software (IBM, Armonk, NY, USA). The normality test of the data sets was performed by the Shapiro-Wilk test or the Kolmogorov-Smirnov test if *n* > 50, and homogeneity of variance test of the data sets was performed by the Levene’s test. Two-tailed unpaired *t*-test was used for comparing two independent groups. Multiple group comparisons were assessed using one-way ANOVA, two-way ANOVA or two-way repeated measures ANOVA, followed by the Bonferroni’s post-hoc test when significant main effects or interactions were detected. Mann–Whitney *U* test or Kruskal-Wallis H test was used when normality was violated. Full statistical analyses corresponding to each figure are provided in Supplementary information, Table S2. *P < 0.05, **P < 0.01, and ***P < 0.001.

## Supplementary information

Supplementary information, Fig. S1

Supplementary information, Fig. S2

Supplementary information, Fig. S3

Supplementary information, Fig. S4

Supplementary information, Fig. S5

Supplementary information, Fig. S6

Supplementary information, Fig. S7

Supplementary information, Fig. S8

Supplementary information, Fig. S9

Supplementary information, Fig. S10

Supplementary information, Fig. S11

Supplementary information, Fig. S12

Supplementary information, Fig. S13

Supplementary information, Fig. S14

Supplementary information, Fig. S15

Supplementary information, Fig. S16

Supplementary information, Fig. S17

Supplementary information, Fig. S18

Supplementary information, Table S1

Supplementary information, Table S2

## Data Availability

The RNA-seq data of *F*- and *N*-RAM engram cells have been submitted to NCBI Gene Expression Omnibus (GEO) under accession number PRJNA1067898.

## Code availability

Custom codes will be provided upon request.
