# Supplementary material for: Disturbed engram network caused by NPTX downregulation underlies aging-related contextual fear memory deficits
Source: Cell Res. 2025 Aug 1;35(9):656–74. doi: 10.1038/s41422-025-01157-w (PMC12408839; doi:10.1038/s41422-025-01157-w)
Supplement: Supplementary file 1 — Supplementary information, Fig. S1 [file 41422_2025_1157_MOESM1_ESM.pdf]

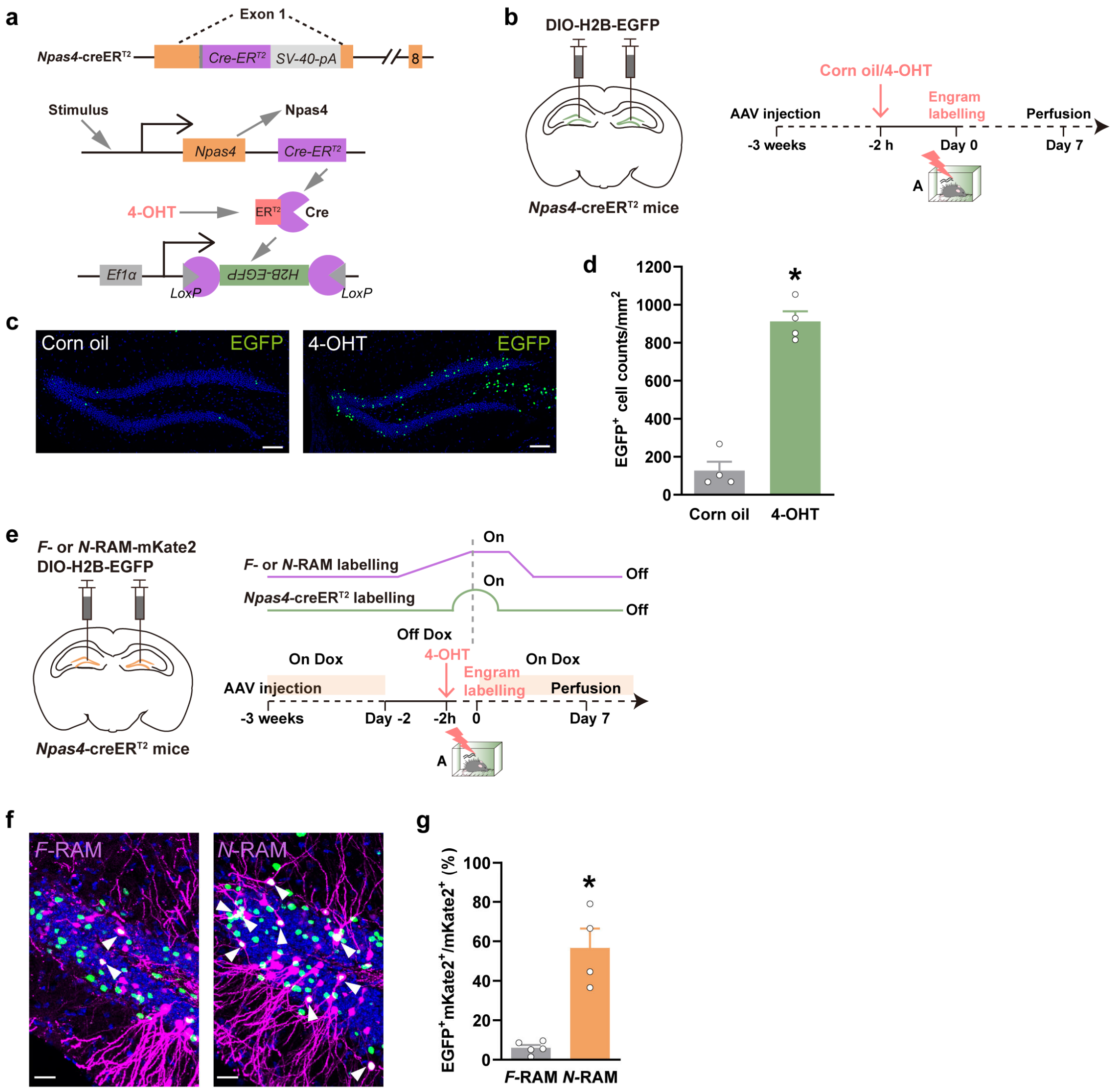

a
Exon 1
Npas4-creER T2
Cre-ER T2
SV-40-pA
8
Stimulus
Npas4
Npas4
Cre-ER T2
4-OHT
ER T2
Cre
Ef1α
H2B-EGFP
LoxP
LoxP
b
DIO-H2B-EGFP
Npas4-creER T2 mice
Corn oil/4-OHT
AAV injection
Engram labelling
Perfusion
-3 weeks
-2 h
Day 0
Day 7
A
c
Corn oil
EGFP
4-OHT
EGFP
d
EGFP+ cell counts/mm2
Corn oil
4-OHT
e
F- or N-RAM-mKate2
DIO-H2B-EGFP
Npas4-creER T2 mice
F- or N-RAM labelling
Npas4-creER T2 labelling
On
On
Off
Off
Off Dox
On Dox
4-OHT
On Dox
AAV injection
Engram labelling
Perfusion
-3 weeks
Day -2
-2h
0
Day 7
A
f
F-RAM
N-RAM
g
EGFP+mKate2+/mKate2+ (%)
F-RAM
N-RAM

Fig. S1 Generation of *Npas4-CreER^{T2}* mice and the overlapping analysis of *F*-RAM and *N*-RAM engram cells in DG. a Diagram of the targeting strategy for generating *Npas4-CreER^{T2}* mice. **b** Diagram of AAV injection and experimental scheme to label *Npas4*$^{+}$ neurons. **c, d** Representative confocal images and quantification of *Npas4*$^{+}$ cells with or without 4-OHT injection. Green: *Npas4*$^{+}$ engram cells, EGFP. Scale bar: 100 μm (corn oil, n = 4 mice; 4-OHT, n = 4 mice). **e** Diagram of AAV injection and experimental scheme to label *Npas4*$^{+}$ and *F*- or *N*-RAM neurons. **f, g** Representative confocal images and overlapping analysis of *Npas4*$^{+}$ and *F*-or *N*-RAM cells. Green: *Npas4*$^{+}$ engram cells, EGFP, purple, *F*- or *N*-RAM cells, mKate2, blue, DAPI. White arrows indicate the colocalized neurons. Scale bar: 30 μm (*F*-RAM, n = 5 mice; *N*-RAM, n = 4 mice). Data are presented as mean ± S.E.M; *$P < 0.05$.
