# Supplementary material for: Disturbed engram network caused by NPTX downregulation underlies aging-related contextual fear memory deficits
Source: Cell Res. 2025 Aug 1;35(9):656–74. doi: 10.1038/s41422-025-01157-w (PMC12408839; doi:10.1038/s41422-025-01157-w)
Supplement: Supplementary file 2 — Supplementary information, Fig. S2 [file 41422_2025_1157_MOESM2_ESM.pdf]

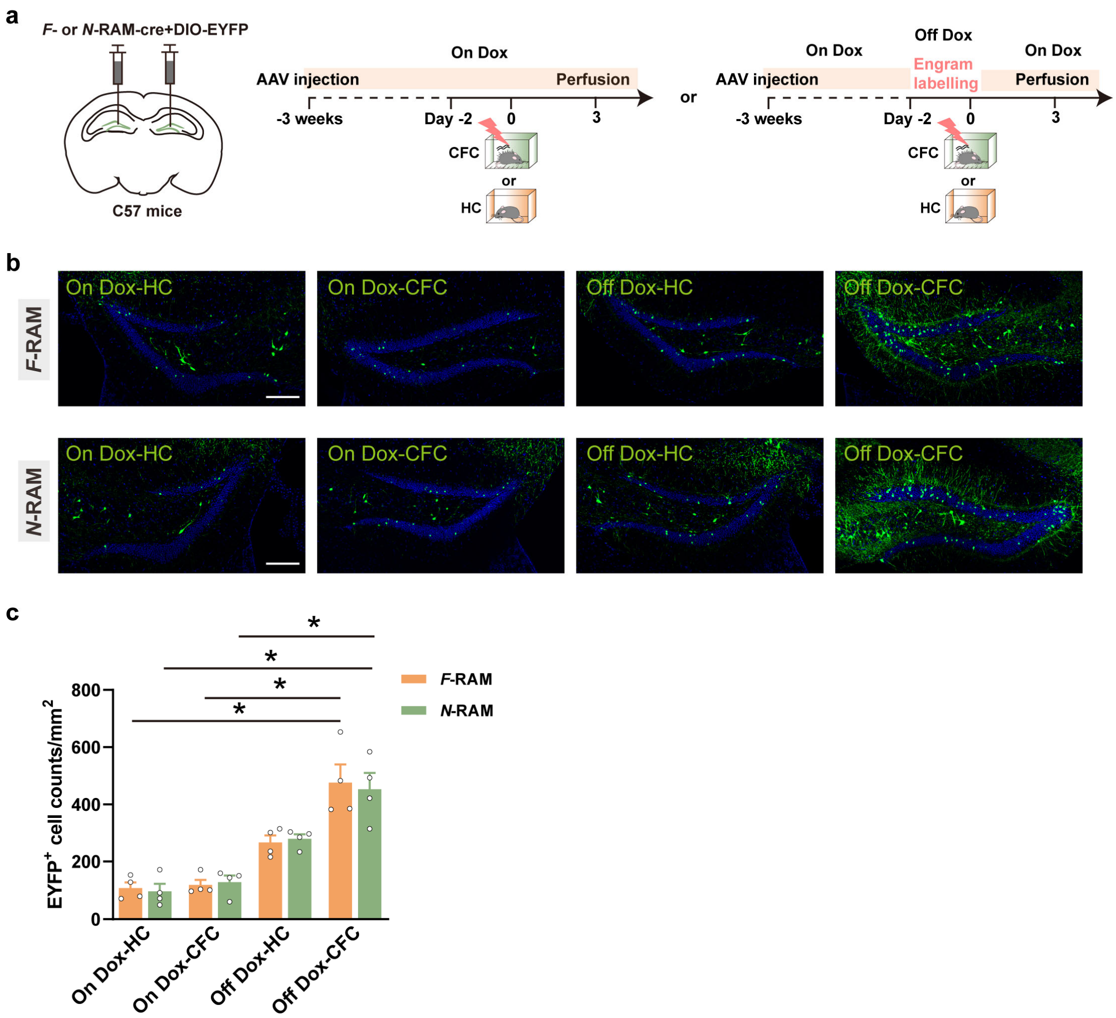

a
F- or N-RAM-cre+DIO-EYFP
C57 mice
On Dox
AAV injection
Perfusion
-3 weeks
Day -2
0
3
CFC
or
HC
or
On Dox
Off Dox
Engram labelling
On Dox
AAV injection
Perfusion
-3 weeks
Day -2
0
3
CFC
or
HC
b
F-RAM
N-RAM
On Dox-HC
On Dox-CFC
Off Dox-HC
Off Dox-CFC
c
EYFP+ cell counts/mm2
800
600
400
200
0
F-RAM
N-RAM
On Dox-HC
On Dox-CFC
Off Dox-HC
Off Dox-CFC

Fig. S2 Labeling specificity validation of *F*-RAM-Cre and *N*-RAM-Cre systems a Diagram of AAV injection and experimental scheme. **b** Representative images of $EYFP^+$ *F*-RAM and *N*-RAM cells in DG under on/off Dox-HC/CFC conditions. Green: EYFP, Blue: DAPI. Scale bar: 100 μm. **c** Quantification of $EYFP^+$ cells in DG under different conditions. (*F*-RAM On dox-HC, n = 4 mice; *N*-RAM On dox-HC, n = 4 mice; *F*-RAM On dox-CFC, n = 4 mice; *N*-RAM On dox-CFC, n = 4 mice; *F*-RAM Off dox-HC, n = 4 mice; *N*-RAM Off dox-HC, n = 4 mice; *F*-RAM Off dox-CFC, n = 4 mice; *N*-RAM Off dox-CFC, n = 4 mice). Data are presented as mean ± S.E.M; $*P < 0.05$.
