# Supplementary material for: Disturbed engram network caused by NPTX downregulation underlies aging-related contextual fear memory deficits
Source: Cell Res. 2025 Aug 1;35(9):656–74. doi: 10.1038/s41422-025-01157-w (PMC12408839; doi:10.1038/s41422-025-01157-w)
Supplement: Supplementary file 3 — Supplementary information, Fig. S3 [file 41422_2025_1157_MOESM3_ESM.pdf]

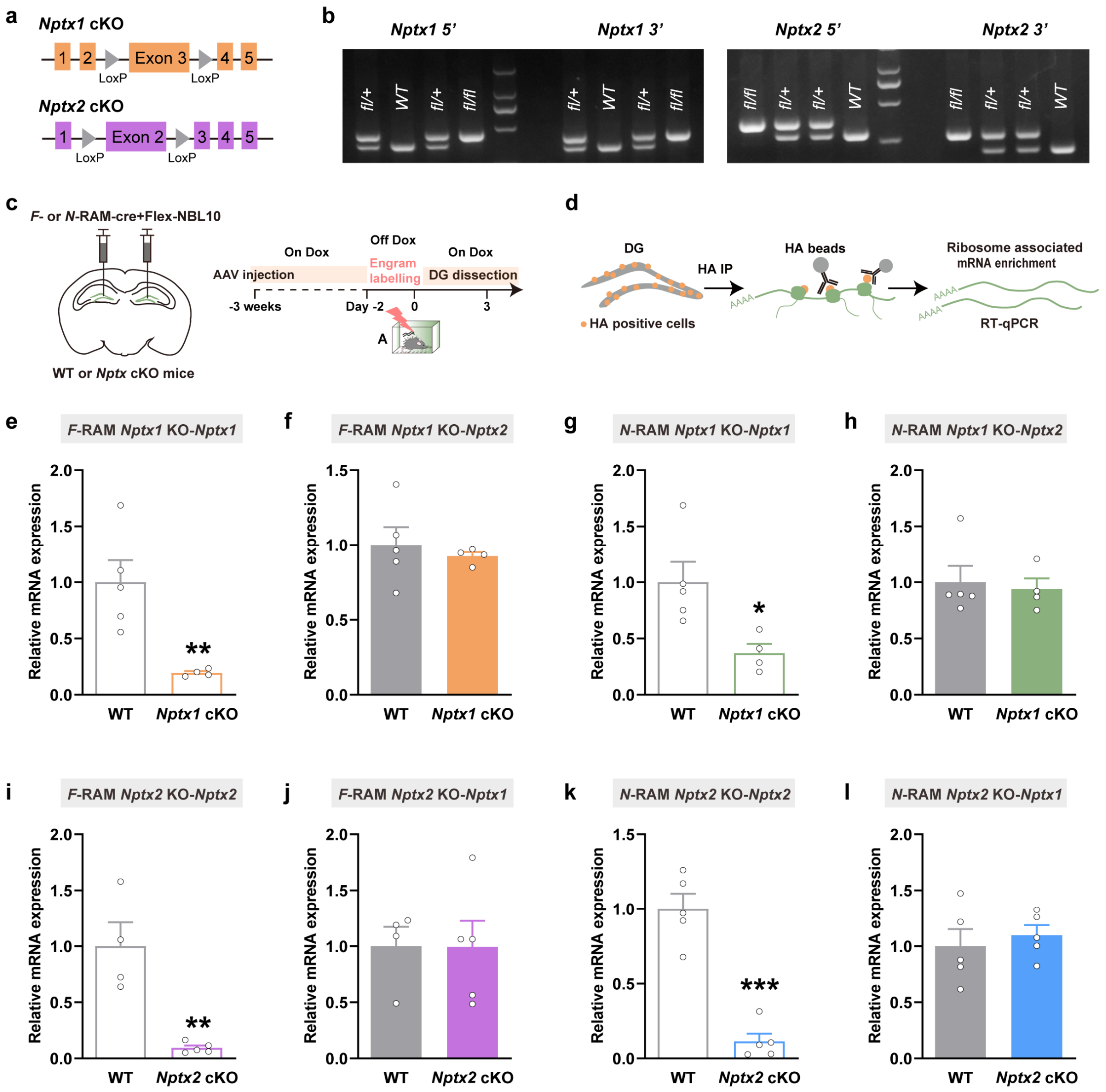
a
Nptx1 cKO
1 2 Exon 3 4 5
LoxP
LoxP
Nptx2 cKO
1 Exon 2 3 4 5
LoxP
LoxP
b
Nptx1 5'
Nptx1 3'
Nptx2 5'
Nptx2 3'
fl/+ WT fl/+ fl/fl
fl/+ WT fl/+ fl/fl
fl/fl fl/+ fl/+ WT
fl/fl fl/+ fl/+ WT
c
F- or N-RAM-cre+Flex-NBL10
WT or Nptx cKO mice
On Dox
Off Dox
On Dox
AAV injection
Engram labelling
DG dissection
-3 weeks
Day -2
0
3
A
d
DG
HA IP
HA beads
Ribosome associated mRNA enrichment
HA positive cells
RT-qPCR
e
F-RAM Nptx1 KO-Nptx1
f
F-RAM Nptx1 KO-Nptx2
g
N-RAM Nptx1 KO-Nptx1
h
N-RAM Nptx1 KO-Nptx2
i
F-RAM Nptx2 KO-Nptx2
j
F-RAM Nptx2 KO-Nptx1
k
N-RAM Nptx2 KO-Nptx2
l
N-RAM Nptx2 KO-Nptx1
Relative mRNA expression
WT
Nptx1 cKO
Nptx2 cKO
**
*
***

Fig. S3 Generation of *Nptx1* cKO and *Nptx2* cKO mice and RT-qPCR validation in DG engrams. **a** Diagram of the targeting strategy for generating *Nptx1* cKO and *Nptx2* cKO mice. **b** Genotyping PCR results for $Nptx1^{+/+}$ (*Nptx1* WT), $Nptx1^{fl/+}$ (*Nptx1* heterozygote) $Nptx1^{fl/fl}$ (*Nptx1* cKO), $Nptx2^{+/+}$ (*Nptx2* WT), $Nptx2^{fl/+}$ (*Nptx2* heterozygote) $Nptx2^{fl/fl}$ and (*Nptx2* cKO) mice. **c, d** Diagram of AAV injection, experimental scheme to label *F*-RAM and *N*-RAM ensembles in WT and *Nptxs* cKO mice and RiboTag enrichment of *F*- and *N*-RAM transcriptomes by utilizing the AAV virus containing Cre-dependent expression of N terminus of ribosomal subunit protein Rpl10a (NBL10). **e, f** RT-qPCR analysis of *Nptx1* exon 3 and *Nptx2* exon 2 mRNA expression in *F*-RAM ensemble of WT and *Nptx1* cKO mice (WT, n = 5 mice; *Nptx1* cKO, n = 4 mice). **g, h** RT-qPCR analysis of *Nptx1* exon 3 and *Nptx2* exon 2 mRNA expression in *N*-RAM ensemble of WT and *Nptx1* cKO mice (WT, n = 5 mice; *Nptx1* cKO, n = 4 mice). **i, j** RT-qPCR analysis of *Nptx2* exon 2 and *Nptx1* exon 3 mRNA expression in *F*-RAM ensemble of WT and *Nptx2* cKO mice (WT, n = 4 mice; *Nptx2* cKO, n = 5 mice). **k, l** RT-qPCR analysis of *Nptx2* exon 2 and *Nptx1* exon 3 mRNA expression in *N*-RAM ensemble of WT and *Nptx2* cKO mice (WT, n = 5 mice; *Nptx2* cKO, n = 5 mice). Data are presented as mean ± S.E.M; **P* < 0.05, ***P* < 0.01, ****P* < 0.001.
