# Supplementary material for: Disturbed engram network caused by NPTX downregulation underlies aging-related contextual fear memory deficits
Source: Cell Res. 2025 Aug 1;35(9):656–74. doi: 10.1038/s41422-025-01157-w (PMC12408839; doi:10.1038/s41422-025-01157-w)
Supplement: Supplementary file 5 — Supplementary information, Fig. S5 [file 41422_2025_1157_MOESM5_ESM.pdf]

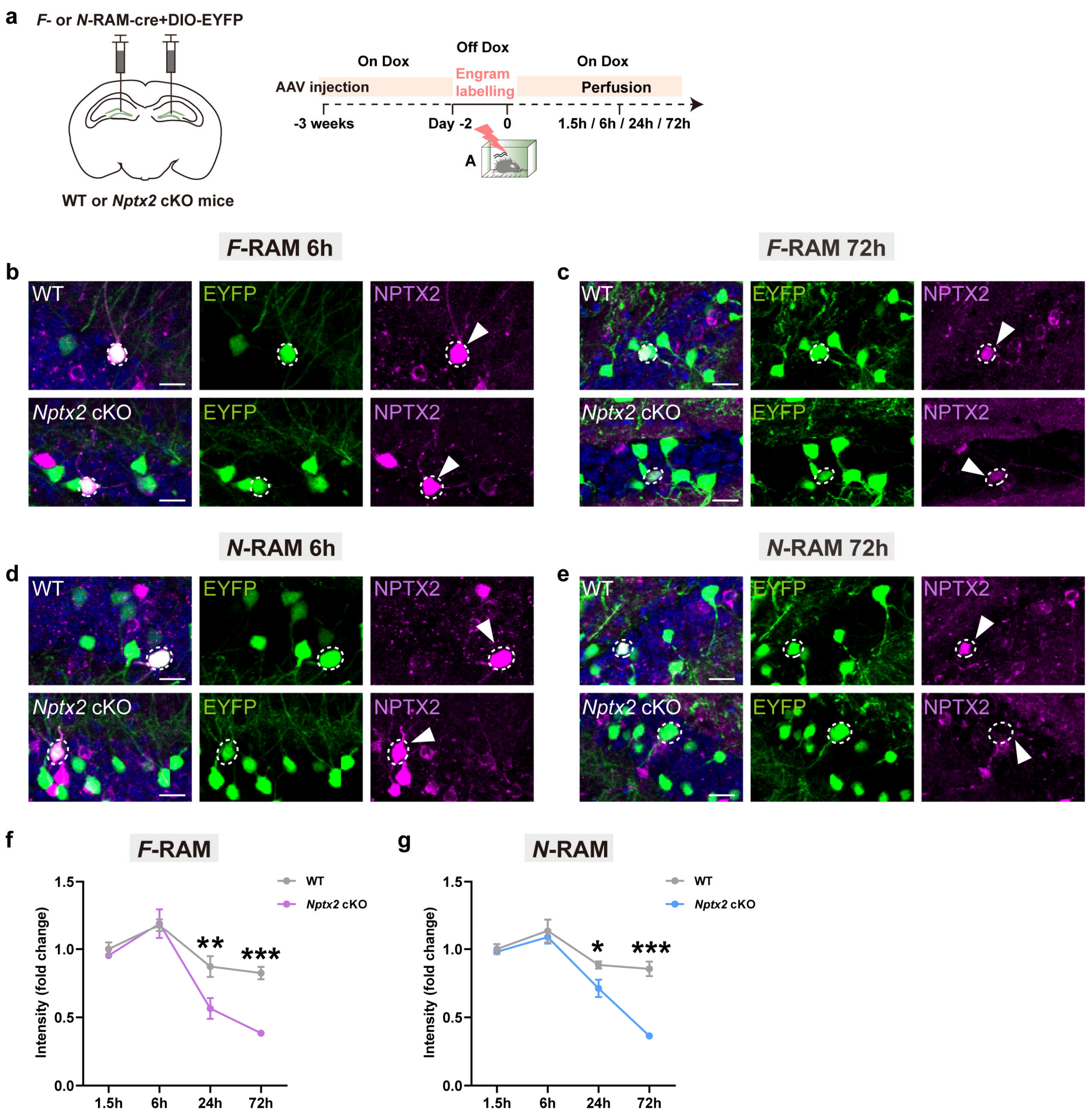
a
F- or N-RAM-cre+DIO-EYFP
WT or Nptx2 cKO mice
On Dox
Off Dox
On Dox
AAV injection
Engram labelling
Perfusion
-3 weeks
Day -2
0
1.5h / 6h / 24h / 72h
A
F-RAM 6h
F-RAM 72h
N-RAM 6h
N-RAM 72h
b
c
d
e
WT
EYFP
NPTX2
Nptx2 cKO
f
F-RAM
g
N-RAM
WT
Nptx2 cKO
Intensity (fold change)
1.5h
6h
24h
72h
**
***
*
***

Fig. S5 IHC validation of NPTX2 knockout in DG engram ensembles. a Diagram of AAV injection and experimental scheme to label *F*-RAM and *N*-RAM ensembles in WT and *Nptx2* cKO mice. **b-e** Representative confocal images of *F*- or *N*-RAM cells colocalizing with NPTX2. Green: EYFP, Purple: NPTX2, Blue: DAPI. Dashed white lines and white arrows outline cells. Scale bar: 10 μm. **f, g** The average NPTX2 fluorescence intensity of *F*- or *N*-RAM cells in WT and *Nptx2* cKO mice (*F*-RAM: WT 1.5h, n = 4 mice; *Nptx2* cKO 1.5h, n = 4 mice; WT 6h, n = 4 mice; *Nptx2* cKO 6h, n = 3 mice; WT 24h, n = 4 mice; *Nptx2* cKO 24h, n = 3 mice; WT 72h, n = 4 mice; *Nptx2* cKO 72h, n = 3 mice; *N*-RAM: WT 1.5h, n = 3 mice; *Nptx2* cKO 1.5h, n = 3 mice; WT 6h, n = 4 mice; *Nptx2* cKO 6h, n = 3 mice; WT 24h, n = 4 mice; *Nptx2* cKO 24h, n = 3 mice; WT 72h, n = 4 mice; *Nptx2* cKO 72h, n = 3 mice). Data are presented as mean ± S.E.M; $^{**}P < 0.01$, $^{***}P < 0.001$.
