# Supplementary material for: Disturbed engram network caused by NPTX downregulation underlies aging-related contextual fear memory deficits
Source: Cell Res. 2025 Aug 1;35(9):656–74. doi: 10.1038/s41422-025-01157-w (PMC12408839; doi:10.1038/s41422-025-01157-w)
Supplement: Supplementary file 6 — Supplementary information, Fig. S6 [file 41422_2025_1157_MOESM6_ESM.pdf]

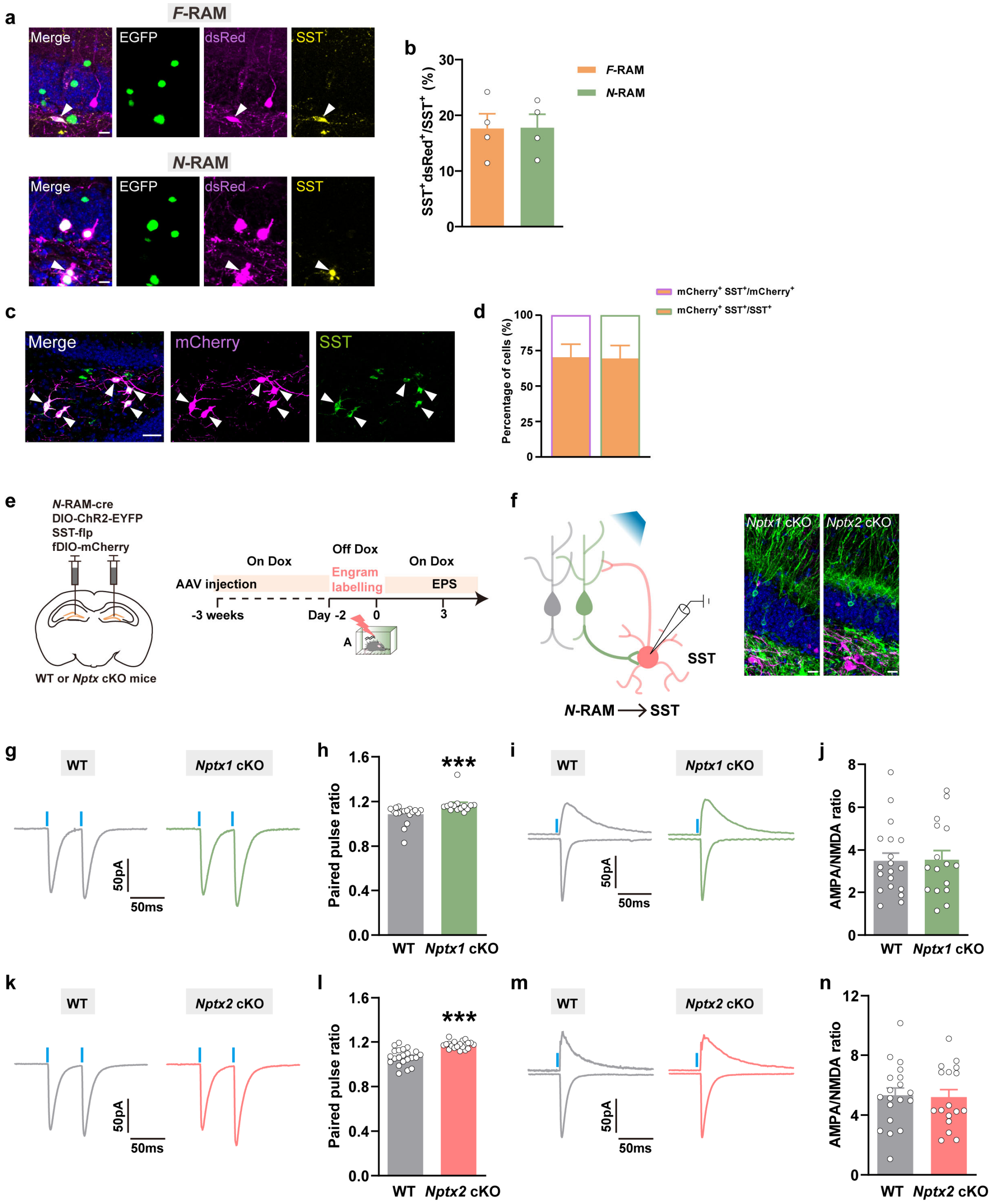
a
F-RAM
Merge
EGFP
dsRed
SST
N-RAM
b
SST+dsRed+/SST+ (%)
F-RAM
N-RAM
c
Merge
mCherry
SST
d
mCherry+ SST+/mCherry+
mCherry+ SST+/SST+
Percentage of cells (%)
e
N-RAM-cre
DIO-ChR2-EYFP
SST-flp
fDIO-mCherry
WT or Nptx cKO mice
On Dox
Off Dox
On Dox
AAV injection
Engram labelling
EPS
-3 weeks
Day -2
0
3
A
f
Nptx1 cKO
Nptx2 cKO
SST
N-RAM → SST
g
WT
Nptx1 cKO
50pA
50ms
h
Paired pulse ratio
***
WT
Nptx1 cKO
i
WT
Nptx1 cKO
50pA
50ms
j
AMPA/NMDA ratio
WT
Nptx1 cKO
k
WT
Nptx2 cKO
50pA
50ms
l
Paired pulse ratio
***
WT
Nptx2 cKO
m
WT
Nptx2 cKO
50pA
50ms
n
AMPA/NMDA ratio
WT
Nptx2 cKO

Fig. S6 The effects of *Nptxs* depletion in *N*-RAM ensemble on the plasticity of DG SST^+ interneurons. **a** Representative confocal images of DG local $dsRed^+$ cells immunostaining with SST. Green: EGFP, Purple: dsRed, Yellow: SST, Blue: DAPI. White arrows indicate the $dsRed^+$ SST^+ colocalized cells. Scale bar: 5 μm. **b** The percentages of colocalized cells in DG total SST^+ cells (*F*-RAM, n = 4 mice; *N*-RAM, n = 4 mice). **c, d** Representative confocal images and overlap analysis of SST-mCherry colocalizing with SST antibody. Green: SST antibody, Purple: mCherry, Blue: DAPI. Scale bar: 10 μm. n = 6. **e** Diagram of AAV injection and experimental scheme to label *N*-RAM engram ensemble. **f** Diagram of photostimulation and whole-cell patch clamp recordings (left) and representative expression of *N*-RAM engram cells and SST^+ interneurons (Right). Green: *N*-RAM engram cells of *Nptx1* and *Nptx2* cKO mice, EYFP, Purple: SST^+ interneurons, mCherry, Blue: DAPI. Scale bar: 10 μm. **g, h** Representative traces and quantification of opto-evoked PPR recorded from WT and *Nptx1* cKO mice (WT, n = 18 neurons from 3 mice; *Nptx1* cKO, n = 14 neurons from 3 mice). **i, j** Representative traces of opto-evoked AMPA-EPSC, NMDA-EPSC and the average A/N ratio recorded from WT and *Nptx1* cKO mice (WT, n = 19 neurons from 3 mice; *Nptx1* cKO, n = 16 neurons from 3 mice). **k, l** Representative traces and quantification of opto-evoked PPR recorded from WT and *Nptx2* cKO mice (WT, n = 22 neurons from 4 mice; *Nptx2* cKO, n = 20 neurons from 4 mice). **m, n** Representative traces of opto-evoked AMPA-EPSC, NMDA-EPSC and the average A/N ratio recorded from WT and *Nptx2* cKO mice (WT, n = 19 neurons from 4 mice; *Nptx2* cKO, n = 17 neurons from 4 mice). Data are presented as mean ± S.E.M; ****P*

< 0.001.
