# Supplementary material for: Disturbed engram network caused by NPTX downregulation underlies aging-related contextual fear memory deficits
Source: Cell Res. 2025 Aug 1;35(9):656–74. doi: 10.1038/s41422-025-01157-w (PMC12408839; doi:10.1038/s41422-025-01157-w)
Supplement: Supplementary file 7 — Supplementary information, Fig. S7 [file 41422_2025_1157_MOESM7_ESM.pdf]

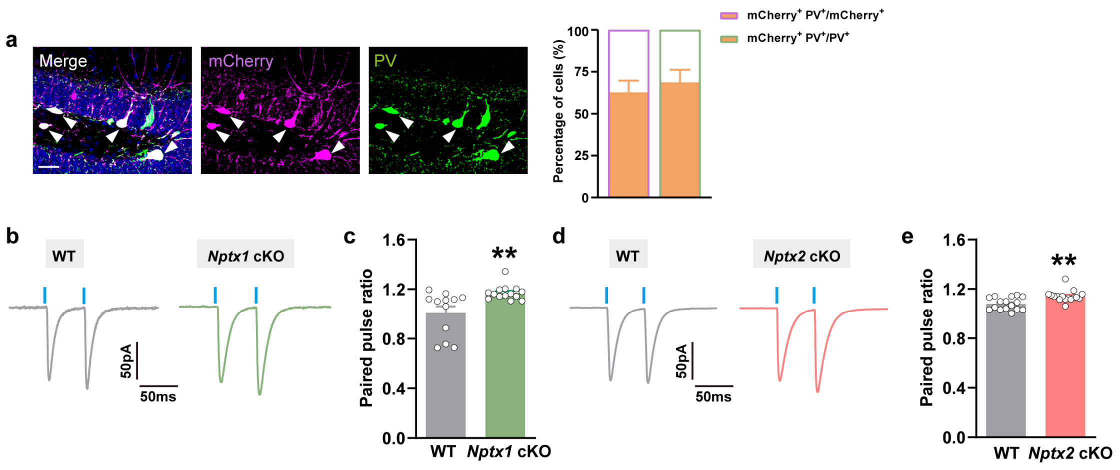

a
Merge
mCherry
PV
Percentage of cells (%)
100
75
50
25
0
mCherry+ PV+/mCherry+
mCherry+ PV+/PV+
b
WT
Nptx1 cKO
50pA
50ms
c
Paired pulse ratio
1.6
1.2
0.8
0.4
0.0
**
WT
Nptx1 cKO
d
WT
Nptx2 cKO
50pA
50ms
e
Paired pulse ratio
1.6
1.2
0.8
0.4
0.0
**
WT
Nptx2 cKO

Fig. S7 The effects of *Nptxs* depletion in *N*-RAM ensemble on pre-synaptic glutamate release to DG PV$^+$ interneurons. a Representative confocal images and overlapping analysis of PV-mCherry colocalizing with PV antibody. Green: PV antibody, Purple: mCherry, Blue: DAPI. Scale bar: 10 μm. n = 5. **b, c** Representative traces and quantification of opto-evoked PPR recorded from WT and *Nptx1* cKO mice (WT, n = 12 neurons from 4 mice; *Nptx1* cKO, n = 14 neurons from 4 mice). **d, e** Representative traces and quantification of opto-evoked PPR recorded from WT and *Nptx2* cKO mice (WT, n = 16 neurons from 3 mice; *Nptx2* cKO, n = 16 neurons from 5 mice). Data are presented as mean ± S.E.M; $^{**}P < 0.01$.
