# Supplementary material for: Disturbed engram network caused by NPTX downregulation underlies aging-related contextual fear memory deficits
Source: Cell Res. 2025 Aug 1;35(9):656–74. doi: 10.1038/s41422-025-01157-w (PMC12408839; doi:10.1038/s41422-025-01157-w)
Supplement: Supplementary file 8 — Supplementary information, Fig. S8 [file 41422_2025_1157_MOESM8_ESM.pdf]

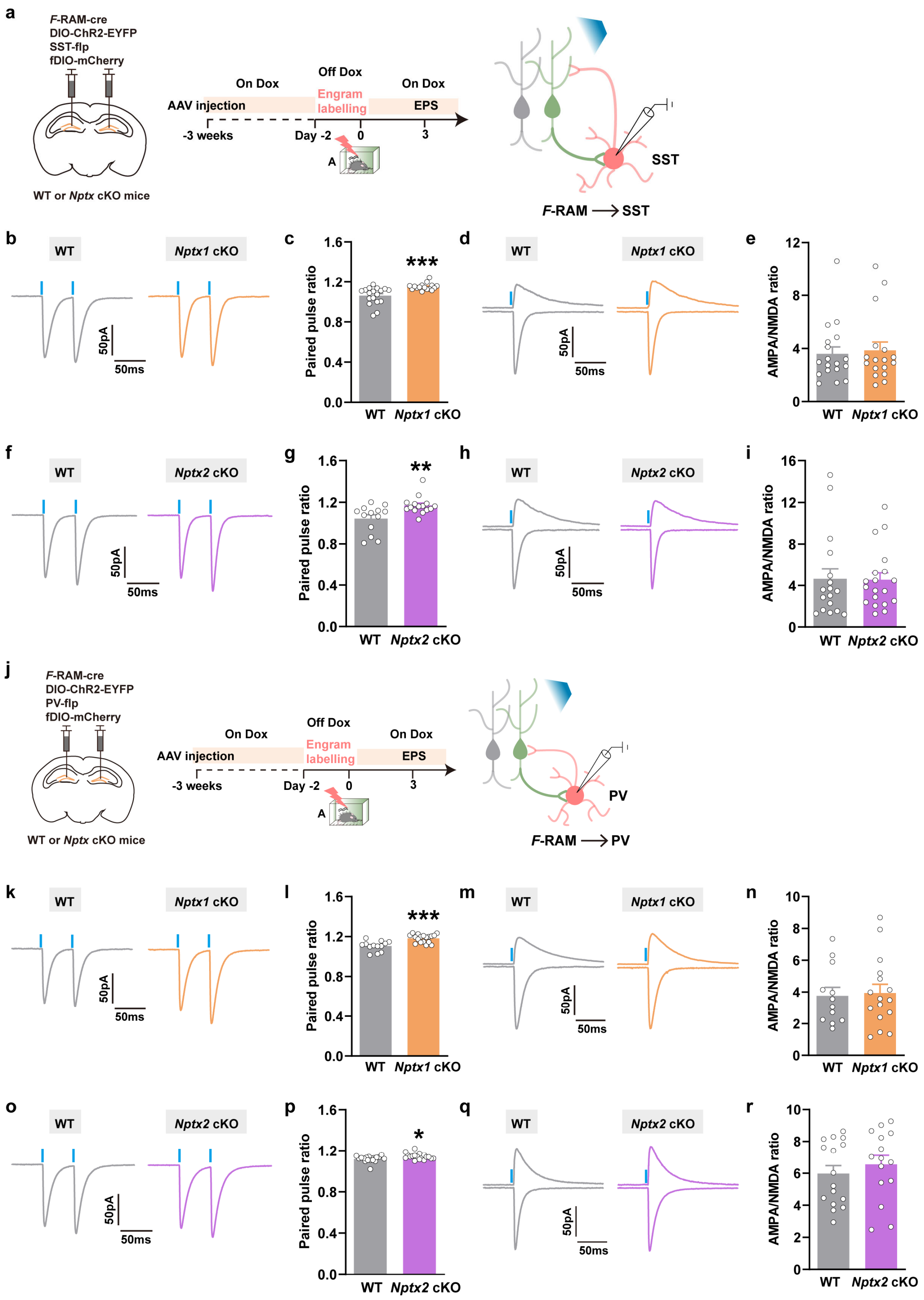
a
F-RAM-cre
DIO-ChR2-EYFP
SST-flp
fDIO-mCherry
WT or Nptx cKO mice
On Dox
Off Dox
On Dox
AAV injection
Engram labelling
EPS
-3 weeks
Day -2
0
3
A
SST
F-RAM ⟶ SST
b
WT
Nptx1 cKO
50pA
50ms
c
Paired pulse ratio
***
WT
Nptx1 cKO
d
WT
Nptx1 cKO
50pA
50ms
e
AMPA/NMDA ratio
WT
Nptx1 cKO
f
WT
Nptx2 cKO
50pA
50ms
g
Paired pulse ratio
**
WT
Nptx2 cKO
h
WT
Nptx2 cKO
50pA
50ms
i
AMPA/NMDA ratio
WT
Nptx2 cKO
j
F-RAM-cre
DIO-ChR2-EYFP
PV-flp
fDIO-mCherry
WT or Nptx cKO mice
On Dox
Off Dox
On Dox
AAV injection
Engram labelling
EPS
-3 weeks
Day -2
0
3
A
PV
F-RAM ⟶ PV
k
WT
Nptx1 cKO
50pA
50ms
l
Paired pulse ratio
***
WT
Nptx1 cKO
m
WT
Nptx1 cKO
50pA
50ms
n
AMPA/NMDA ratio
WT
Nptx1 cKO
o
WT
Nptx2 cKO
50pA
50ms
p
Paired pulse ratio
*
WT
Nptx2 cKO
q
WT
Nptx2 cKO
50pA
50ms
r
AMPA/NMDA ratio
WT
Nptx2 cKO

**Fig. S8 The effects of *Nptxs* depletion in *F*-RAM ensemble on the plasticity of DG SST$^+$ and PV$^+$ interneurons. a** Diagram of AAV injection and experimental scheme to label *F*-RAM engram ensembles. **b, c** Representative traces and quantification of opto-evoked PPR recorded from WT and *Nptx1* cKO mice (WT, n = 18 neurons from 3 mice; *Nptx1* cKO, n = 16 neurons from 4 mice). **d, e** Representative traces of opto-evoked AMPA-EPSC, NMDA-EPSC and the average A/N ratio recorded from WT and *Nptx1* cKO mice (WT, n = 18 neurons from 3 mice; *Nptx1* cKO, n = 17 neurons from 4 mice). **f, g** Representative traces and quantification of opto-evoked PPR recorded from WT and *Nptx2* cKO mice (WT, n = 13 neurons from 4 mice; *Nptx2* cKO, n = 15 neurons from 4 mice). **h, i** Representative traces of opto-evoked AMPA-EPSC, NMDA-EPSC and the average A/N ratio recorded from WT and *Nptx2* cKO mice (WT, n = 16 neurons from 4 mice; *Nptx2* cKO, n = 19 neurons from 4 mice). **j** Diagram of AAV injection and experimental scheme to label *F*-RAM engram ensembles. **k, l** Representative traces and quantification of opto-evoked PPR recorded from WT and *Nptx1* cKO mice (WT, n = 13 neurons from 3 mice; *Nptx1* cKO, n = 18 neurons from 4 mice). **m, n** Representative traces of opto-evoked AMPA-EPSC, NMDA-EPSC and the average A/N ratio recorded from WT and *Nptx1* cKO mice (WT, n = 12 neurons from 3 mice; *Nptx1* cKO, n = 16 neurons from 4 mice). **o, p** Representative traces and quantification of opto-evoked PPR recorded from WT and *Nptx2* cKO mice (WT, n = 18 neurons from 4 mice; *Nptx2* cKO, n = 18 neurons from 4 mice). **q, r** Representative traces of opto-evoked AMPA-EPSC, NMDA-EPSC and the average A/N ratio recorded from WT and *Nptx2* cKO mice (WT, n = 16 neurons from 4 mice; *Nptx2* cKO, n = 15 neurons from 4 mice). Data are presented as mean ± S.E.M; **P* < 0.05, ***P* < 0.01, ****P* < 0.001.
