# Supplementary material for: Disturbed engram network caused by NPTX downregulation underlies aging-related contextual fear memory deficits
Source: Cell Res. 2025 Aug 1;35(9):656–74. doi: 10.1038/s41422-025-01157-w (PMC12408839; doi:10.1038/s41422-025-01157-w)
Supplement: Supplementary file 9 — Supplementary information, Fig. S9 [file 41422_2025_1157_MOESM9_ESM.pdf]

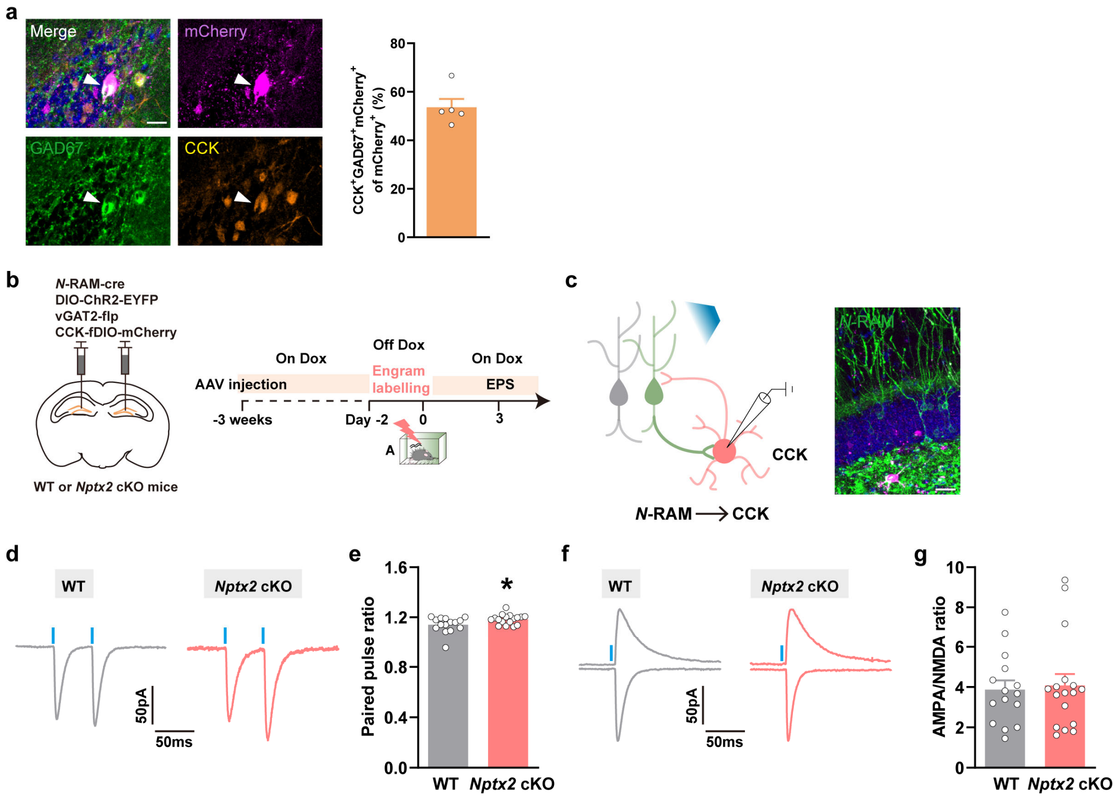

a
Merge
mCherry
GAD67
CCK
CCK+GAD67+mCherry+ of mCherry+ (%)
b
N-RAM-cre
DIO-ChR2-EYFP
vGAT2-flp
CCK-fDIO-mCherry
WT or Nptx2 cKO mice
On Dox
Off Dox
On Dox
AAV injection
Engram labelling
EPS
-3 weeks
Day -2
0
3
A
c
N-RAM
CCK
N-RAM ⟶ CCK
d
WT
Nptx2 cKO
50pA
50ms
e
Paired pulse ratio
*
WT
Nptx2 cKO
f
WT
Nptx2 cKO
50pA
50ms
g
AMPA/NMDA ratio
WT
Nptx2 cKO

Fig. S9 The effects of *Nptxs* depletion in *N*-RAM ensemble on the plasticity of DG CCK$^+$ interneurons. a Representative confocal images and overlap analysis of CCK-mCherry colocalizing with GAD67 and CCK antibody. Green: GAD67 antibody, Yellow, CCK antibody, Purple: mCherry, Blue: DAPI. Scale bar: 10 µm. n = 5. **b** Diagram of AAV injection and experimental scheme to label *N*-RAM engram ensembles. **c** Diagram of photostimulation and whole-cell patch clamp recordings (left) and representative expression of engram cells and CCK$^+$ interneurons (right). Green: *N*-RAM engram cells, EYFP, Purple: CCK$^+$ interneurons, mCherry, Blue: DAPI. Scale bar: 10 µm. **d, e** Representative traces and quantification of opto-evoked PPR recorded from WT and *Nptx2* cKO mice (WT, n = 15 neurons from 4 mice; *Nptx2* cKO, n = 17 neurons from 4 mice). **f, g** Representative traces of opto-evoked AMPA-EPSC, NMDA-EPSC and the average A/N ratio recorded from WT and *Nptx2* cKO mice (WT, n = 15 neurons from 4 mice; *Nptx2* cKO, n = 17 neurons from 4 mice). Data are presented as mean ± S.E.M; *$P < 0.05$.
