# Supplementary material for: Disturbed engram network caused by NPTX downregulation underlies aging-related contextual fear memory deficits
Source: Cell Res. 2025 Aug 1;35(9):656–74. doi: 10.1038/s41422-025-01157-w (PMC12408839; doi:10.1038/s41422-025-01157-w)
Supplement: Supplementary file 10 — Supplementary information, Fig. S10 [file 41422_2025_1157_MOESM10_ESM.pdf]

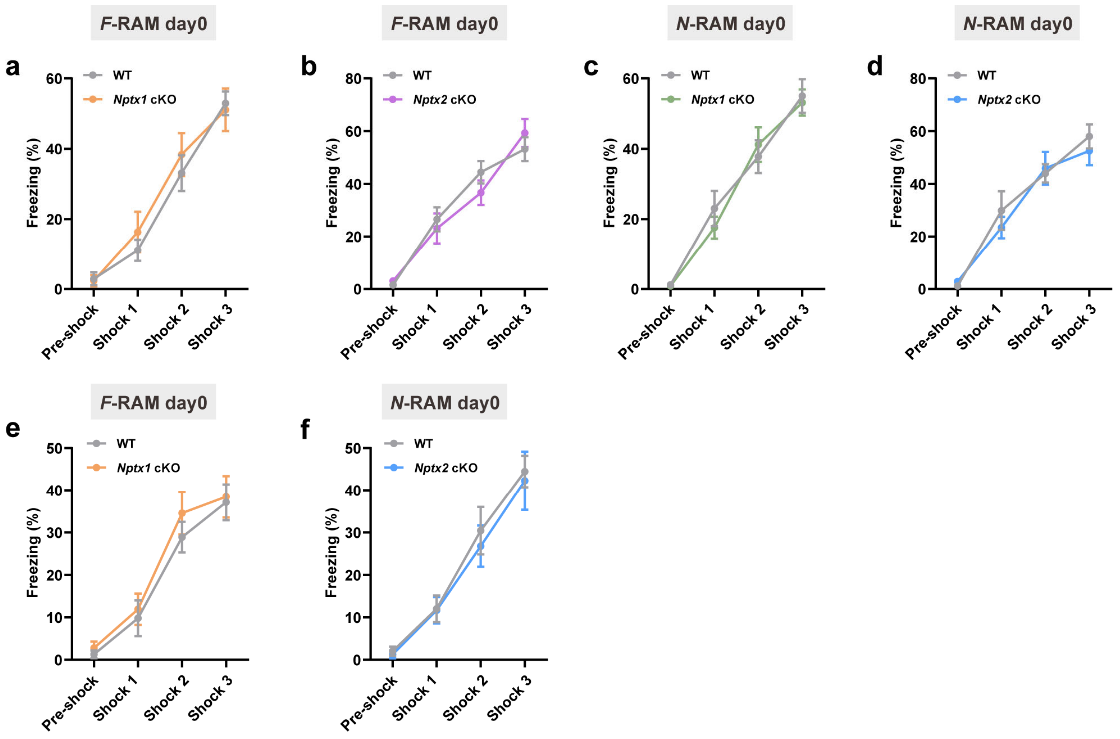
F-RAM day0
F-RAM day0
N-RAM day0
N-RAM day0
F-RAM day0
N-RAM day0
a
b
c
d
e
f
WT
Nptx1 cKO
Nptx2 cKO
Freezing (%)
Pre-shock
Shock 1
Shock 2
Shock 3

Fig. S10 Freezing levels of WT and *Nptxs* cKO mice during fear conditioning. a The quantification for freezing levels of WT and *Nptx1* cKO mice during CFC (*F*-RAM) (WT, n = 14 mice; *Nptx1* cKO, n = 11 mice). **b** The quantification for freezing levels of WT and *Nptx2* cKO mice during CFC (*F*-RAM) (WT, n = 14 mice; *Nptx2* cKO, n = 13 mice). **c** The quantification for freezing levels of WT and *Nptx1* cKO mice during CFC (*N*-RAM) (WT, n = 18 mice; *Nptx1* cKO, n = 15 mice). **d** The quantification for freezing levels of WT and *Nptx2* cKO mice during CFC (*N*-RAM) (WT, n = 13 mice; *Nptx2* cKO, n = 12 mice). **e** The quantification for freezing levels of WT and *Nptx1* cKO mice during CFC (*F*-RAM) (WT, n = 11 mice; *Nptx1* cKO, n = 11 mice). **f** The quantification for freezing levels of WT and *Nptx2* cKO mice during CFC (*N*-RAM) (WT, n = 10 mice; *Nptx2* cKO, n = 9 mice). Data are presented as mean ± S.E.M.
