# Supplementary material for: Disturbed engram network caused by NPTX downregulation underlies aging-related contextual fear memory deficits
Source: Cell Res. 2025 Aug 1;35(9):656–74. doi: 10.1038/s41422-025-01157-w (PMC12408839; doi:10.1038/s41422-025-01157-w)
Supplement: Supplementary file 11 — Supplementary information, Fig. S11 [file 41422_2025_1157_MOESM11_ESM.pdf]

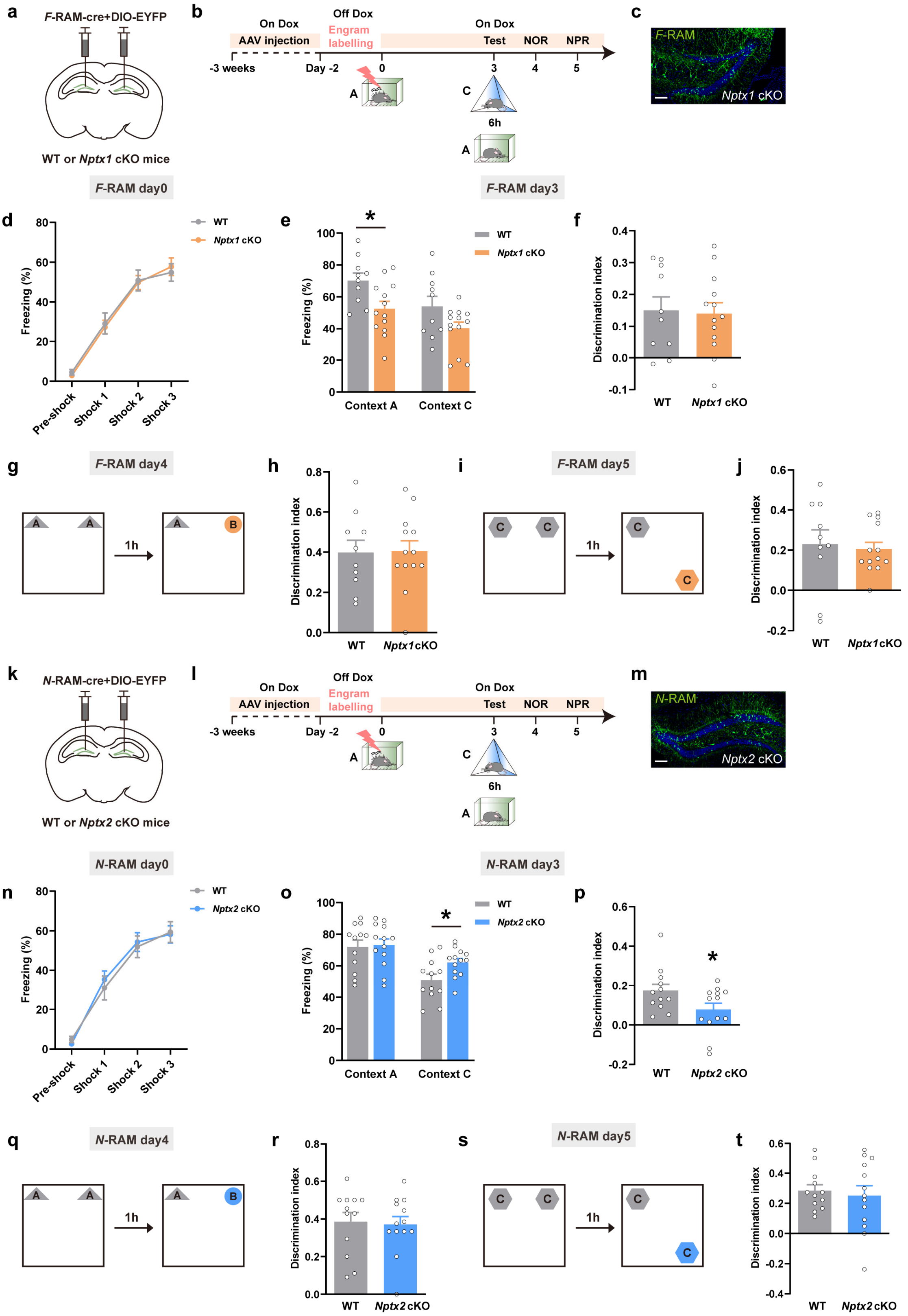
a
F-RAM-cre+DIO-EYFP
WT or Nptx1 cKO mice
b
On Dox
AAV injection
Off Dox
Engram labelling
On Dox
Test
NOR
NPR
-3 weeks
Day -2
0
3
4
5
A
C
6h
A
c
F-RAM
Nptx1 cKO
F-RAM day0
F-RAM day3
d
Freezing (%)
WT
Nptx1 cKO
Pre-shock
Shock 1
Shock 2
Shock 3
e
Freezing (%)
WT
Nptx1 cKO
Context A
Context C
f
Discrimination index
WT
Nptx1 cKO
g
F-RAM day4
1h
A
B
h
Discrimination index
WT
Nptx1cKO
i
F-RAM day5
1h
C
j
Discrimination index
WT
Nptx1cKO
k
N-RAM-cre+DIO-EYFP
WT or Nptx2 cKO mice
l
On Dox
AAV injection
Off Dox
Engram labelling
On Dox
Test
NOR
NPR
-3 weeks
Day -2
m
N-RAM
Nptx2 cKO
N-RAM day0
N-RAM day3
n
Freezing (%)
WT
Nptx2 cKO
Pre-shock
Shock 1
Shock 2
Shock 3
o
Freezing (%)
WT
Nptx2 cKO
Context A
Context C
p
Discrimination index
WT
Nptx2 cKO
q
N-RAM day4
1h
r
Discrimination index
WT
Nptx2 cKO
s
N-RAM day5
1h
t
Discrimination index
WT
Nptx2 cKO

Fig. S11 The effects of *Nptxs* depletion in *F*-RAM and *N*-RAM ensembles on the expression of contextual fear, NOR and NPR memories. a, k Diagram of AAV injection. **b, l** Experimental scheme of CFC and memory retrieval. **c, m** Representative expression of *F*- and *N*-RAM engram cells in DG. Green: *F*-RAM or *N*-RAM ensemble, EYFP, Blue: DAPI. Scale bar: 100 μm. **d** The quantification for freezing levels of WT and *Nptx1* cKO mice during CFC (*F*-RAM) (WT, n = 10 mice; *Nptx1* cKO, n = 13 mice). **e, f** The freezing percentage and discrimination index of WT and *Nptx1* cKO mice tested in context C and context A at day 3 (*F*-RAM) (WT, n = 10 mice; *Nptx1* cKO, n = 13 mice). **g, q** Experimental scheme of NOR. **i, s** Experimental scheme of NPR. **h, j** Discrimination index of WT and *Nptx1* cKO mice (WT, n = 10 mice; *Nptx1* cKO, n = 13 mice). **n** The quantification for freezing levels of WT and *Nptx2* cKO mice during CFC (*N*-RAM) (WT, n = 12 mice; *Nptx2* cKO, n = 13 mice). **o, p** The freezing percentage and discrimination index of WT and *Nptx2* cKO mice tested in context C and context A at day 3 (*N*-RAM) (WT, n = 12 mice; *Nptx2* cKO, n = 13 mice). **r, t** Discrimination index of WT and *Nptx2* cKO mice (WT, n = 12 mice; *Nptx2* cKO, n = 13 mice). Data are presented as mean ± S.E.M.
