# Supplementary material for: Disturbed engram network caused by NPTX downregulation underlies aging-related contextual fear memory deficits
Source: Cell Res. 2025 Aug 1;35(9):656–74. doi: 10.1038/s41422-025-01157-w (PMC12408839; doi:10.1038/s41422-025-01157-w)
Supplement: Supplementary file 12 — Supplementary information, Fig. S12 [file 41422_2025_1157_MOESM12_ESM.pdf]

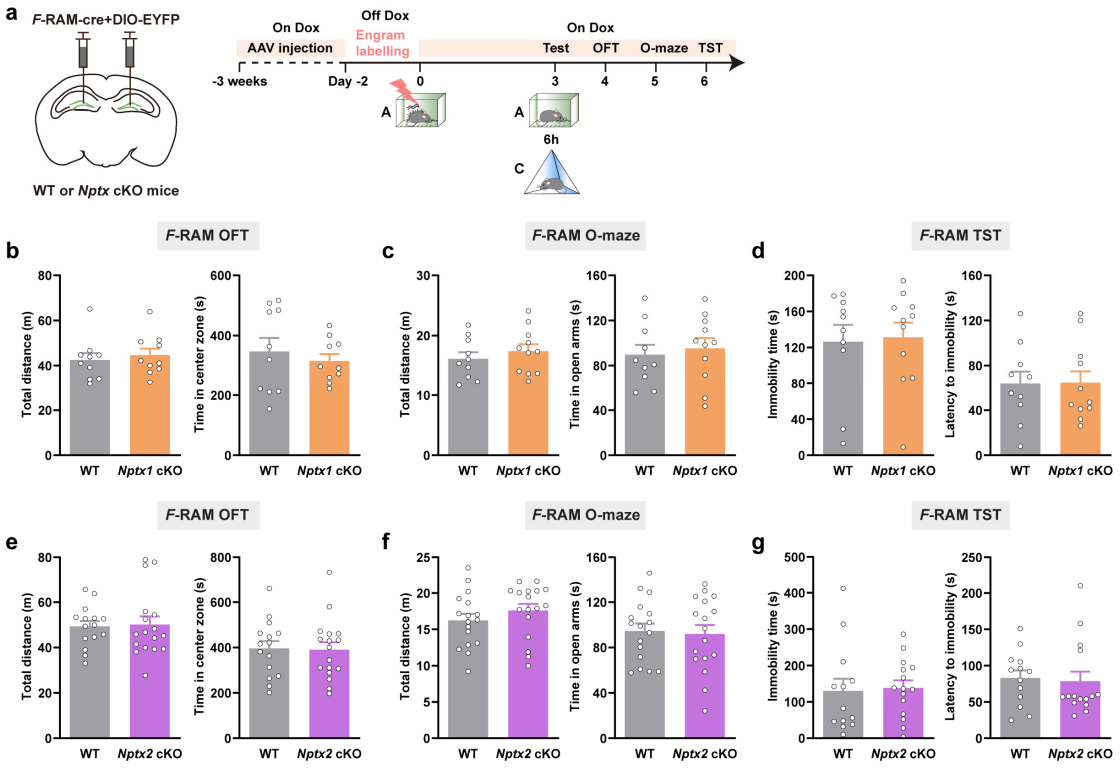

a
F-RAM-cre+DIO-EYFP
WT or Nptx cKO mice
On Dox
AAV injection
Off Dox
Engram labelling
On Dox
Test
OFT
O-maze
TST
-3 weeks
Day -2
0
3
4
5
6
A
A
6h
C
b
F-RAM OFT
Total distance (m)
Time in center zone (s)
WT
Nptx1 cKO
c
F-RAM O-maze
Total distance (m)
Time in open arms (s)
d
F-RAM TST
Immobility time (s)
Latency to immobility (s)
e
F-RAM OFT
Nptx2 cKO
f
F-RAM O-maze
g
F-RAM TST

Fig. S12 The effects of *Nptxs* depletion in *F*-RAM ensemble activated by CFC on the locomotion, anxiety or depression level of mice. a Diagram of AAV injection and experimental scheme of open field test (OFT), O-maze test and tail suspension test (TST). **b** The average distance travelled (left) and time spent in center zone (right) of OFT for WT and *Nptx1* cKO mice (WT, n = 10 mice; *Nptx1* cKO, n = 10 mice). **c** The average distance travelled (left) and time spent in open arms (right) of O-maze test for WT and *Nptx1* cKO mice (WT, n = 10 mice; *Nptx1* cKO, n = 11 mice). **d** The average immobility time (left) and latency to immobility (right) of TST for WT and *Nptx1* cKO mice (WT, n = 10 mice; *Nptx1* cKO, n = 11 mice). **e** The average distance travelled (left) and time spent in center zone (right) of OFT for WT and *Nptx2* cKO mice (WT, n = 15 mice; *Nptx2* cKO, n = 17 mice). **f** The average distance travelled (left) and time spent in open arms (right) of O-maze test for WT and *Nptx2* cKO mice (WT, n = 17 mice; *Nptx2* cKO, n = 17 mice). **g** The average immobility time (left) and latency to immobility (right) of TST for WT and *Nptx2* cKO mice (WT, n = 13 mice; *Nptx2* cKO, n = 15 mice). Data are presented as mean ± S.E.M.
