# Supplementary material for: Disturbed engram network caused by NPTX downregulation underlies aging-related contextual fear memory deficits
Source: Cell Res. 2025 Aug 1;35(9):656–74. doi: 10.1038/s41422-025-01157-w (PMC12408839; doi:10.1038/s41422-025-01157-w)
Supplement: Supplementary file 14 — Supplementary information, Fig. S14 [file 41422_2025_1157_MOESM14_ESM.pdf]

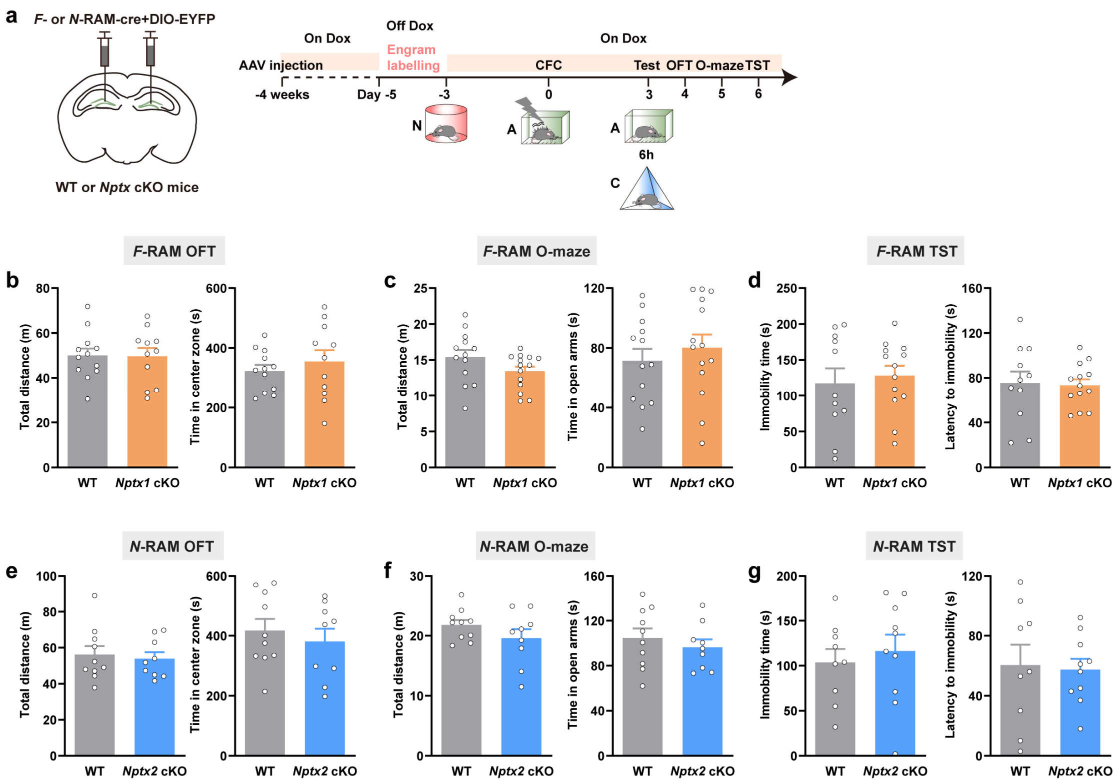

a
F- or N-RAM-cre+DIO-EYFP
WT or Nptx cKO mice
On Dox
Off Dox
Engram labelling
On Dox
AAV injection
CFC
Test OFT O-maze TST
-4 weeks
Day -5
-3
0
3
4
5
6
N
A
A
6h
C
F-RAM OFT
F-RAM O-maze
F-RAM TST
N-RAM OFT
N-RAM O-maze
N-RAM TST
Total distance (m)
Time in center zone (s)
Time in open arms (s)
Immobility time (s)
Latency to immobility (s)
WT
Nptx1 cKO
Nptx2 cKO

Fig. S14 The effects of *Nptxs* depletion in *F*-and *N*-RAM ensembles activated by novel context exposure on the locomotion, anxiety or depression level of mice. a Diagram of AAV injection and experimental scheme of OFT, O-maze test and TST. **b** The average distance travelled (left) and time spent in center zone (right) of OFT for WT and *Nptx1* cKO mice (*F*-RAM) (WT, n = 12 mice; *Nptx1* cKO, n = 11 mice). **c** The average distance travelled (left) and time spent in open arms (right) of O-maze test for WT and *Nptx1* cKO mice (*F*-RAM) (WT, n = 13 mice; *Nptx1* cKO, n = 14 mice). **d** The average immobility time (left) and latency to immobility (right) of TST for WT and *Nptx1* cKO mice (*F*-RAM) (WT, n = 11 mice; *Nptx1* cKO, n = 13 mice). **e** The average distance travelled (left) and time spent in center zone (right) of OFT for WT and *Nptx2* cKO mice (*N*-RAM) (WT, n = 10 mice; *Nptx2* cKO, n = 9 mice). **f** The average distance travelled (left) and time spent in open arms (right) of O-maze test for WT and *Nptx2* cKO mice (*N*-RAM) (WT, n = 10 mice; *Nptx2* cKO, n = 9 mice). **g** The average immobility time (left) and latency to immobility (right) of TST for WT and *Nptx2* cKO mice (*N*-RAM) (WT, n = 9 mice; *Nptx2* cKO, n = 10 mice). Data are presented as mean ± S.E.M.
