# Supplementary material for: Disturbed engram network caused by NPTX downregulation underlies aging-related contextual fear memory deficits
Source: Cell Res. 2025 Aug 1;35(9):656–74. doi: 10.1038/s41422-025-01157-w (PMC12408839; doi:10.1038/s41422-025-01157-w)
Supplement: Supplementary file 15 — Supplementary information, Fig. S15 [file 41422_2025_1157_MOESM15_ESM.pdf]

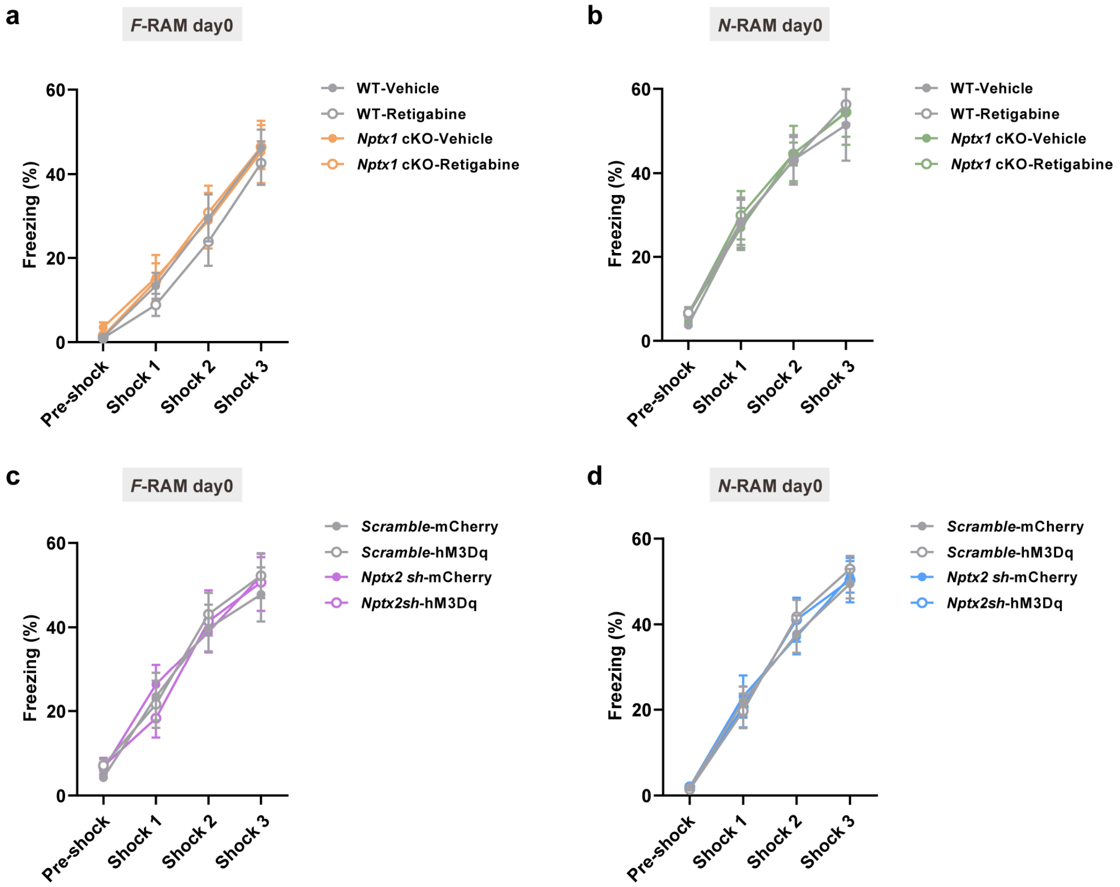
a
F-RAM day0
b
N-RAM day0
c
F-RAM day0
d
N-RAM day0
Freezing (%)
Pre-shock
Shock 1
Shock 2
Shock 3
WT-Vehicle
WT-Retigabine
Nptx1 cKO-Vehicle
Nptx1 cKO-Retigabine
Scramble-mCherry
Scramble-hM3Dq
Nptx2 sh-mCherry
Nptx2sh-hM3Dq

**Fig. S15 The effects of activating Kv7.2 or PV$^+$ interneurons in DG on the freezing levels of mice during CFC. a, b** The quantitative analysis for freezing levels of WT-Vehicle, WT-Retigabine, *Nptx1* cKO-Vehicle and *Nptx1* cKO-Retigabine mice during CFC (*F*-RAM: WT vehicle, n = 12 mice; WT retigabine, n = 13 mice; *Nptx1* cKO vehicle, n = 10 mice; *Nptx1* cKO retigabine, n = 12 mice; *N*-RAM: WT vehicle, n=10 mice; WT retigabine, n=11 mice; *Nptx1* cKO vehicle, n=11 mice; *Nptx1* cKO retigabine, n=10 mice). **c, d** The quantitative analysis for freezing levels in *Scramble*-mCherry, *Scramble*-hM3Dq, *Nptx2 sh*-mCherry and *Nptx2 sh*-hM3Dq groups during CFC (*F*-RAM: *Scramble*-mcherry, n = 10 mice; *Scramble*-hM3Dq, n = 11 mice; *Nptx2* sh-mcherry, n = 10 mice; *Nptx2* sh-hM3Dq, n = 12 mice; *N*-RAM: *Scramble*-mcherry, n = 16 mice; *Scramble*-hM3Dq, n = 15 mice; *Nptx2* sh-mcherry, n = 15 mice; *Nptx2* sh-hM3Dq, n = 14 mice). Data are presented as mean ± S.E.M.
