# Supplementary material for: Disturbed engram network caused by NPTX downregulation underlies aging-related contextual fear memory deficits
Source: Cell Res. 2025 Aug 1;35(9):656–74. doi: 10.1038/s41422-025-01157-w (PMC12408839; doi:10.1038/s41422-025-01157-w)
Supplement: Supplementary file 16 — Supplementary information, Fig. S16 [file 41422_2025_1157_MOESM16_ESM.pdf]

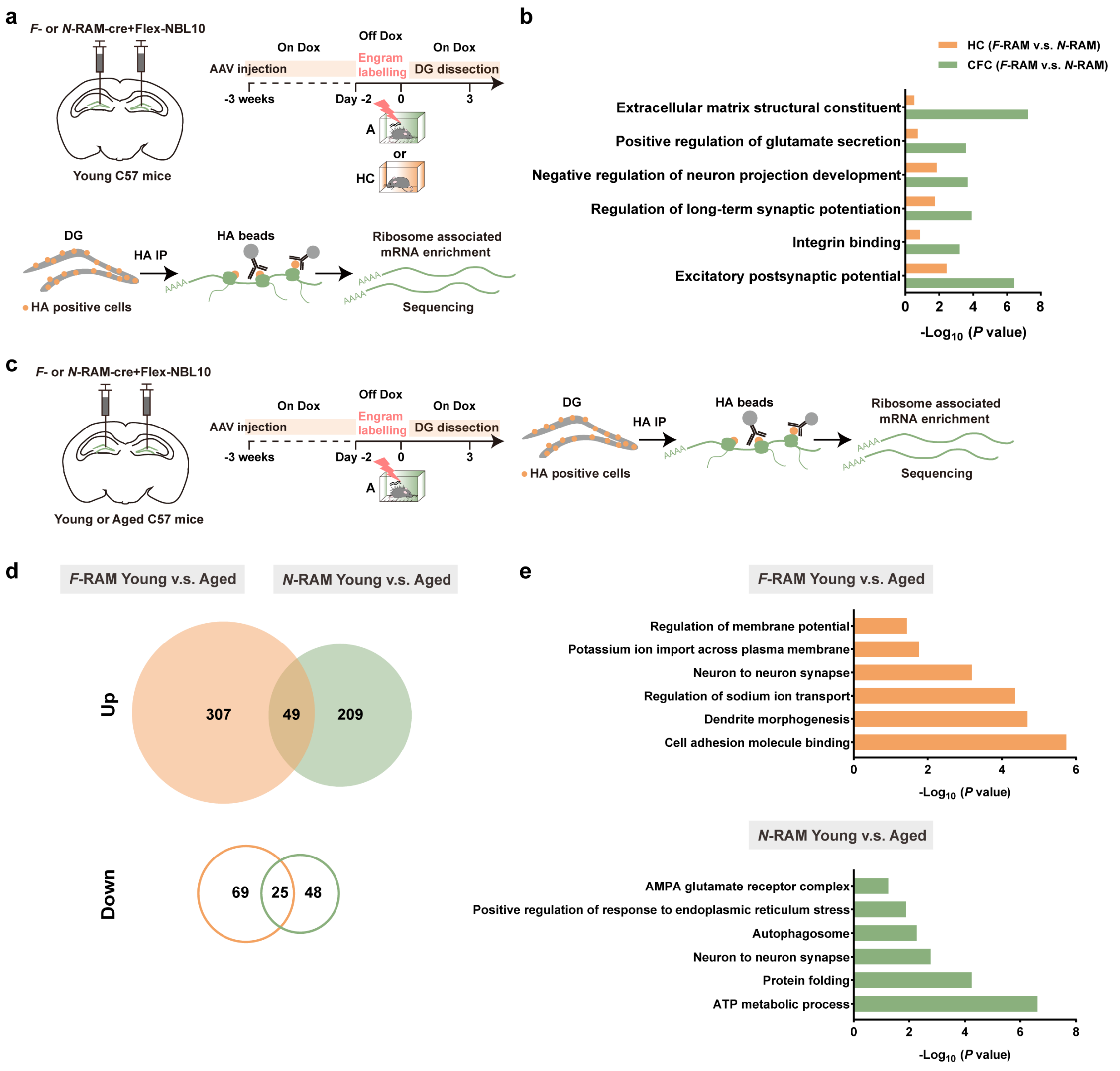
a
F- or N-RAM-cre+Flex-NBL10
Young C57 mice
On Dox
Off Dox
On Dox
AAV injection
Engram labelling
DG dissection
-3 weeks
Day -2
0
3
A
or
HC
DG
HA IP
HA beads
Ribosome associated mRNA enrichment
HA positive cells
Sequencing
b
HC (F-RAM v.s. N-RAM)
CFC (F-RAM v.s. N-RAM)
Extracellular matrix structural constituent
Positive regulation of glutamate secretion
Negative regulation of neuron projection development
Regulation of long-term synaptic potentiation
Integrin binding
Excitatory postsynaptic potential
-Log10 (P value)
c
F- or N-RAM-cre+Flex-NBL10
Young or Aged C57 mice
d
F-RAM Young v.s. Aged
N-RAM Young v.s. Aged
Up
307
49
209
Down
69
25
48
e
F-RAM Young v.s. Aged
Regulation of membrane potential
Potassium ion import across plasma membrane
Neuron to neuron synapse
Regulation of sodium ion transport
Dendrite morphogenesis
Cell adhesion molecule binding
N-RAM Young v.s. Aged
AMPA glutamate receptor complex
Positive regulation of response to endoplasmic reticulum stress
Autophagosome
Neuron to neuron synapse
Protein folding
ATP metabolic process

Fig. S16 Transcriptional networks enrichment in *F*-RAM and *N*-RAM ensembles in young and aged mice. a Diagram of AAV injection, experimental scheme to label *F*-RAM and *N*-RAM ensembles and scheme of RiboTag enrichment of *F*- and *N*-RAM transcriptomes activated in HC or by CFC of young mice. **b** GO enrichment analysis of transcriptional differences in DG *F*-RAM and *N*-RAM ensembles activated in HC or by CFC of young mice (*F*-RAM, HC, n = 4. *N*-RAM, HC, n = 4. *F*-RAM, CFC, n = 4. *N*-RAM, CFC, n = 4). **c** Diagram of AAV injection, experimental scheme to label *F*-RAM and *N*-RAM ensembles and scheme of RiboTag enrichment of *F*- and *N*-RAM transcriptomes in young and aged mice. **d** A Venn diagram showing the upregulation (up) and downregulation (bottom) of *F*- and *N*-RAM transcriptomes during aging and the overlap between these two groups. **e** GO enrichment analysis of transcriptional differences in DG *F*-RAM (up) and *N*-RAM (down) ensembles respectively along with aging (*F*-RAM, young, n = 5. *F*-RAM, aged, n = 5. *N*-RAM, young, n = 6. *N*-RAM, aged, n = 7). Data are presented as mean ± S.E.M.
