# Supplementary material for: Disturbed engram network caused by NPTX downregulation underlies aging-related contextual fear memory deficits
Source: Cell Res. 2025 Aug 1;35(9):656–74. doi: 10.1038/s41422-025-01157-w (PMC12408839; doi:10.1038/s41422-025-01157-w)
Supplement: Supplementary file 17 — Supplementary information, Fig. S17 [file 41422_2025_1157_MOESM17_ESM.pdf]

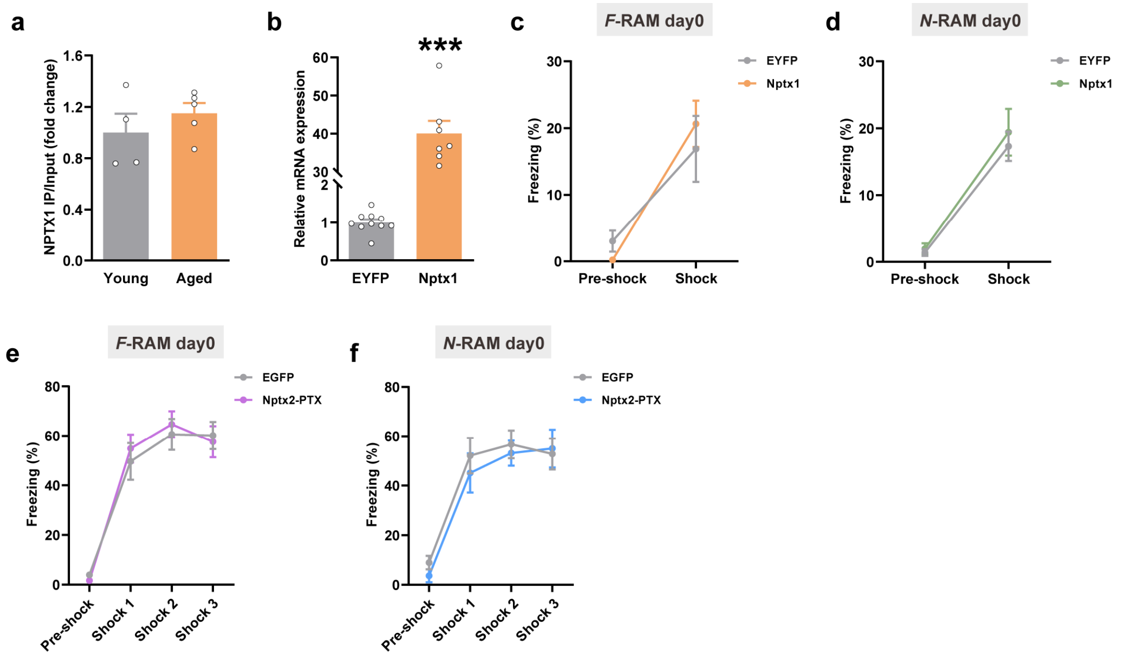

a
NPTX1 IP/Input (fold change)
0.0
0.4
0.8
1.2
1.6
Young
Aged
b
***
Relative mRNA expression
0
1
2
30
40
50
60
EYFP
Nptx1
c
F-RAM day0
EYFP
Nptx1
Freezing (%)
0
10
20
30
Pre-shock
Shock
d
N-RAM day0
EYFP
Nptx1
Freezing (%)
0
10
20
30
Pre-shock
Shock
e
F-RAM day0
EGFP
Nptx2-PTX
Freezing (%)
0
20
40
60
80
Pre-shock
Shock 1
Shock 2
Shock 3
f
N-RAM day0
EGFP
Nptx2-PTX
Freezing (%)
0
20
40
60
80
Pre-shock
Shock 1
Shock 2
Shock 3

Fig. S17 The effects of *Nptxs* overexpression in aged mice on the freezing levels during conditioning. a The quantification of IP-NPTX1 in young and aged mice. **b** RT-qPCR analysis of Nptx1 overexpression efficiency (EYFP, n = 10 mice; Nptx1, n = 7 mice). **c, d** The average freezing levels of EYFP and Nptx1 aged mice during CFC (*F*-RAM: EYFP, n = 11 mice; Nptx1, n = 12 mice; *N*-RAM: EYFP, n = 14 mice; Nptx1, n = 9 mice). **e, f** The average freezing levels of EYFP and Nptx2-PTX aged mice during CFC (*F*-RAM: EGFP, n = 11 mice; Nptx2-PTX, n = 11 mice; *N*-RAM: EGFP, n = 13 mice; Nptx2-PTX, n = 10 mice). Data are presented as mean ± S.E.M; ***$P < 0.001$.
