# Supplementary material for: Disturbed engram network caused by NPTX downregulation underlies aging-related contextual fear memory deficits
Source: Cell Res. 2025 Aug 1;35(9):656–74. doi: 10.1038/s41422-025-01157-w (PMC12408839; doi:10.1038/s41422-025-01157-w)
Supplement: Supplementary file 18 — Supplementary information, Fig. S18 [file 41422_2025_1157_MOESM18_ESM.pdf]

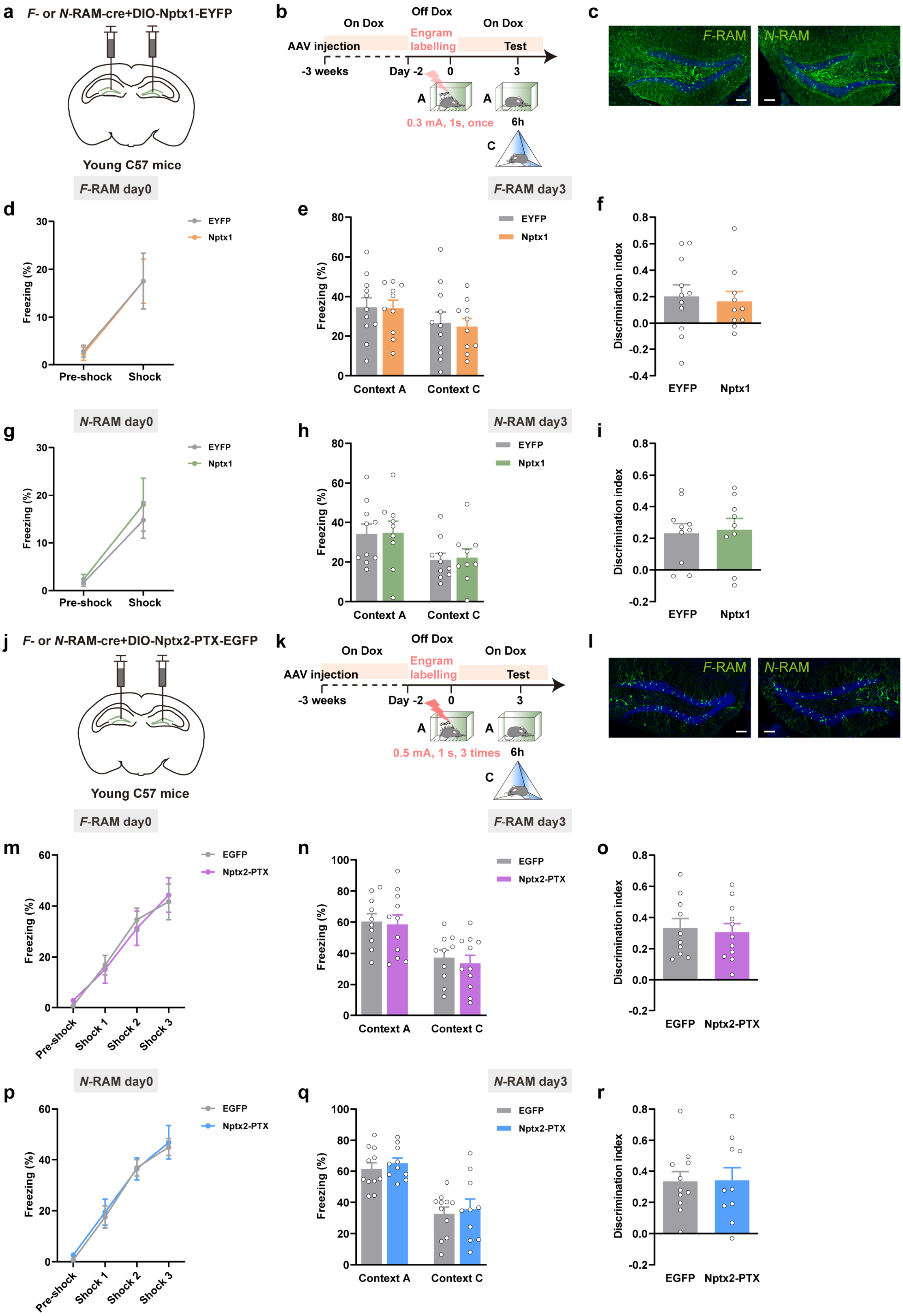

a
F- or N-RAM-cre+DIO-Nptx1-EYFP
Young C57 mice
b
On Dox
Off Dox
On Dox
AAV injection
Engram labelling
Test
-3 weeks
Day -2
0
3
A
A
0.3 mA, 1s, once
6h
C
c
F-RAM
N-RAM
F-RAM day0
F-RAM day3
d
EYFP
Nptx1
Freezing (%)
Pre-shock
Shock
e
EYFP
Nptx1
Freezing (%)
Context A
Context C
f
Discrimination index
EYFP
Nptx1
N-RAM day0
N-RAM day3
g
EYFP
Nptx1
Freezing (%)
Pre-shock
Shock
h
EYFP
Nptx1
Freezing (%)
Context A
Context C
i
Discrimination index
EYFP
Nptx1
j
F- or N-RAM-cre+DIO-Nptx2-PTX-EGFP
Young C57 mice
k
On Dox
Off Dox
On Dox
AAV injection
Engram labelling
Test
-3 weeks
Day -2
0
3
A
A
0.5 mA, 1 s, 3 times
6h
C
l
F-RAM
N-RAM
F-RAM day0
F-RAM day3
m
EGFP
Nptx2-PTX
Freezing (%)
Pre-shock
Shock 1
Shock 2
Shock 3
n
EGFP
Nptx2-PTX
Freezing (%)
Context A
Context C
o
Discrimination index
EGFP
Nptx2-PTX
N-RAM day0
N-RAM day3
p
EGFP
Nptx2-PTX
Freezing (%)
Pre-shock
Shock 1
Shock 2
Shock 3
q
EGFP
Nptx2-PTX
Freezing (%)
Context A
Context C
r
Discrimination index
EGFP
Nptx2-PTX

Fig. S18 The effects of overexpressing NPTXs in DG engram ensembles on memory expression in young mice. a, j Diagram of AAV injection. **b, k** Experimental scheme of memory retrieval test. **c, l** Representative expression of NPTX1 or NPTX2-PTX in *F*- or *N*-RAM engram cells in DG. Green: *F*-RAM or *N*-RAM ensemble, EYFP or EGFP, Blue: DAPI. Scale bar: 100 μm. **d, g** The quantitative analysis for freezing levels of EYFP and Nptx1 young mice during CFC (*F*-RAM: EYFP, n = 11 mice; Nptx1, n = 10 mice; *N*-RAM: EYFP, n = 10 mice; Nptx1, n = 9 mice). **e, f** The freezing percentage and discrimination index of EYFP and Nptx1 young groups (*F*-RAM) (EYFP, n = 11 mice; Nptx1, n = 10 mice). **h, i** The freezing percentage and discrimination index of EYFP and Nptx1 young groups (*N*-RAM) (EYFP, n = 10 mice; Nptx1, n = 9 mice). **m, p** The quantitative analysis for freezing levels of EGFP and NPTX2-PTX young mice during CFC (*F*-RAM: EGFP, n = 10 mice; Nptx2-PTX, n = 11 mice; *N*-RAM: EGFP, n = 11 mice; Nptx2-PTX, n = 10 mice). **n, o** The freezing percentage and discrimination index of EGFP and Nptx2-PTX young groups (*F*-RAM) (EGFP, n = 10 mice; Nptx2-PTX, n = 11 mice). **q, r** The freezing percentage and discrimination index of EGFP and Nptx2-PTX young groups (*N*-RAM) (EGFP, n = 11 mice; Nptx2-PTX, n = 10 mice). Data are presented as mean ± S.E.M.
