# Supplementary material for: Disturbed engram network caused by NPTX downregulation underlies aging-related contextual fear memory deficits
Source: Cell Res. 2025 Aug 1;35(9):656–74. doi: 10.1038/s41422-025-01157-w (PMC12408839; doi:10.1038/s41422-025-01157-w)
Supplement: Supplementary file 19 — Supplementary information, Table S1 [file 41422_2025_1157_MOESM19_ESM.pdf]

List of primers used in this study			
**Primer ID**	**Gene ID**	**Sequence**	**Application**
P1	*Nptx1* -5'	GCTGTAGGGATGCTTGTCTCTGGTG	Genotyping
		AGAAAAGCTGACCCAAGGTCTCTGC	
P2	*Nptx1* -3'	ATTAGCTGCCAGATCTTAGCCCCCT	
		GTGTGTGTCCCTGGTGGTGAAGTTT	
P3	*Nptx2* -5'	GAATGGCTCGAGGCAGGTCCAGTTT	Genotyping
		CGTTACTAAACCCCAGACAGCTCCG	
P4	*Nptx2* -3'	AGTTCTGCCTCTGTTCATCTTGCCA	
		TTCACCTGACCCTTCTGTTCACGAC	
P5	*PV-fl pe*	TGCCGGTCCTATTTACTCGT	Genotyping
		TACTTCTTTAGCGCAAGGGGTAG	
		CTAGGCCACAGAATTGAAAGATCT	
		GTAGGTGGAAATTCTAGCATCATCC	
P6	*Npas4-CreER^{T2}*	AGAGCCTGAGCGAAAAGACC	Genotyping
		CTGCTCACCTCCAGCAAAGA	
		CGCGCGCCTGAAGATATAGA	
P7	*Nptx1*	ACCTCCCTACACCAACGGAT	RT-qPCR
		GGCAGGCTCTTCTTCACCTT	
P8	*Nptx1* -exon3	GCCAAGGTGAAGAAGAGCCT	RT-qPCR
		AGCATAAGAGAAGGGTGTGCC	
P9	*Nptx2* -exon2	GACTTCCGAGAGGTGCTCCA	RT-qPCR
		GGTGAGCCGAGGTCTCATTA	
P10	*Gapdh*	TGGCCTTCCGTGTTCCTAC	RT-qPCR
		GAGTTGCTGTTGAAGTCGCA	
P11	*Nptx1* (TSS -2000~-1500bp)	CCTAGTTCTGCCGGTGTACG	CHIP-qPCR
		AGGTACAGAACGCAGGAACG	
P12	*Nptx1* (TSS -1500~-1000bp)	GTCCATCGCAGACCCGTAAA	CHIP-qPCR
		AAAGAAAACGAAGGCGGGGA	
P13	*Nptx1* (TSS -1000~-500bp)	AACCCCTCCTCCAAAGTCCA	CHIP-qPCR
		TGGGTTTACCGGTCCACTTG	

P14	*Nptx1* (TSS -500~0bp)	GAATCATTGTTCGGCCTCGC	CHIP-qPCR
		GGTTCGCTTGCTCCACACTA	
P15	*Nptx1* (TSS 0-500bp)	GCTTCATCTGCACTTCGGTG	CHIP-qPCR
		AGCTCCCTGATGGTCTCCTT	
P16	*Nptx2* (TSS -2000~-1500bp)	TCCATGTCATGAAAGGGCTGG	CHIP-qPCR
		AGGCCAGGGCAGATCTTAAT	
P17	*Nptx2* (TSS -1500~-1000bp)	TACCTGAGCCATCCTCGCTT	CHIP-qPCR
		TGTGGGGGTTTTCAAGGACAG	
P18	*Nptx2* (TSS -1000~-500bp)	CTCCCAATCTTTTGCGGGGT	CHIP-qPCR
		CGCATGACCTCGGTTTGATG	
P19	*Nptx2* (TSS -500~0bp)	TTCCGAAGCCCAGTTTCCTT	CHIP-qPCR
		CTGTCGTGGGTTGGGTTTCA	
P20	*Nptx2* (TSS 0-500bp)	GTCGTGCAGCAGAAGGAGAC	CHIP-qPCR
		GAGCTCTCGGATGGCTTCTC	
