# Supplementary material for: Disturbed engram network caused by NPTX downregulation underlies aging-related contextual fear memory deficits
Source: Cell Res. 2025 Aug 1;35(9):656–74. doi: 10.1038/s41422-025-01157-w (PMC12408839; doi:10.1038/s41422-025-01157-w)
Supplement: Supplementary file 20 — Supplementary information, Table S2 [file 41422_2025_1157_MOESM20_ESM.pdf]

Figure	Response variable	Groups	n define as	Normality test (Shapiro-Wilk)	Homogeneity of variance (Levene's test)	Statistical test	Test value	P value
1c-MS	Inputs	F-RAM n=4	mouse	df=4 p=0.510	df1=1 df2=7 p=0.554	Kruskal-Wallis H test	H=6.000	P=0.014
		N-RAM n=5		df=5 p=0.182				
1c-HDB		F-RAM n=4		df=4 p=0.271	df1=1 df2=7 p=0.209		H=0.240	P=0.624
		N-RAM n=5		df=5 p=0.894				
1c-PRh		F-RAM n=4		df=4 p=0.364	df1=1 df2=7 p=0.117		H=1.500	P=0.221
		N-RAM n=5		df=5 p=0.804				
1c-LEC		F-RAM n=4		df=4 p=0.983	df1=1 df2=7 p=0.734		H=0.540	P=0.462
		N-RAM n=5		df=5 p=0.147				
1c-MEC		F-RAM n=4		df=4 p=0.686	df1=1 df2=7 p=0.366		H=4.860	P=0.027
		N-RAM n=5		df=5 p=0.912				
1c-DG		F-RAM n=4		df=4 p=0.924	df1=1 df2=7 p=0.223		H=6.000	P=0.014
		N-RAM n=5		df=5 p=0.028				
1e	(dsRed+ChAT)/dsRed	F-RAM n=4	mouse	df=4 p=0.175	df1=1 df2=7 p=0.904	Two-tailed unpaired t-test	t=0.194	P=0.851
		N-RAM n=5		df=5 p=0.650				
1g	(dsRed+vGlut1)/dsRed	F-RAM n=4	mouse	df=4 p=0.920	df1=1 df2=7 p=0.759	Two-tailed unpaired t-test	t=2.613	P=0.004
		N-RAM n=5		df=5 p=0.232				
1i-(-2000~-1500 bp)	Relative enrichment	Fos n=4	mouse	df=4 p=0.079	df1=1 df2=9 p=0.311	Kruskal-Wallis H test	H=1.500	P=0.472
		Npas4 n=4		df=4 p=0.275				
		IgG n=4		df=4 p=0.111				
1i-(-1500~-		Fos n=4		df=4 p=0.498	df1=2 df2=9 p=0.064		H=2.423	P=0.298
		Npas4 n=4		df=4 p=0.668				

1000 bp)		IgG n=4		df=4 p=0.134				
1i-(1000~-500 bp)		Fos n=4		df=4 p=0.116	df1=2 df2=9 p=0.482		H=0.800	P=0.018
		Npas4 n=4		df=4 p=0.006				
		IgG n=4		df=4 p=0.827				
		Fos vs Npas4						P=0.006
		Fos vs IgG						P=0.05
		Npas4 vs IgG						P=0.433
1i-(500~0 bp)		Fos n=4		df=4 p=0.280	df1=2 df2=9 p=0.102		H=2.923	P=0.232
		Npas4 n=4		df=4 p=0.365				
		IgG n=4		df=4 p=0.324				
1i-(0~500 bp)		Fos n=4		df=4 p=0.686	df1=1 df2=7 p=0.366		H=3.962	P=0.138
		Npas4 n=4		df=4 p=0.271				
		IgG n=4		df=4 p=0.271				
1j-(-2000~-1500 bp)	Relative enrichment	Fos n=4	mouse	df=4 p=0.671	df1=2 df2=8 p=0.079	Kruskal-Wallis H test	H=5.693	P=0.058
		Npas4 n=3		df=3 p=0.842				
		IgG n=4		df=4 p=0.098				
1j-(-1500~-1000 bp)		Fos n=4		df=4 p=0.498	df1=2 df2=8 p=0.508		H=2.976	P=0.226
		Npas4 n=3		df=3 p=0.668				
		IgG n=4		df=4 p=0.134				
1j-(1000~-500 bp)		Fos n=4		df=4 p=0.777	df1=2 df2=8 p=0.019		H=6.758	P=0.034
		Npas4 n=3		df=3 p=0.582				
		IgG n=4		df=4 p=0.227				
		Fos vs Npas4						P=0.030
		Fos vs IgG						P=0.075
		Npas4 vs IgG						P=0.393

1j-(500~0 bp)		Fos n=4		df=4 p=0.207	df1=2 df2=8 p=0.701		H=0.601	P=0.740
		Npas4 n=3		df=4 p=0.562				
		IgG n=4		df=4 p=0.111				
1j-(0~500 bp)		Fos n=4		df=4 p=0.289	df1=1 df2=7 p=0.021		H=0.898	P=0.638
		Npas4 n=3		df=4 p=0.864				
		IgG n=4		df=4 p=0.002				
1m	Amplitude	WT-F-RAM- n=13 (from 4 mice)	neuron	df=13 p=0.003	df1=1 df2=23 p=0.651	Kruskal-Wallis H test	H=0.666	P=0.415
		Nptx1 cko-F-RAM- n=12 (from 4 mice)		df=12 p=0.031				
		WT-F-RAM+ n=13 (from 4 mice)		df=13 p=0.376	df1=1 df2=23 p=0.150		H=9.278	P=0.002
		Nptx1 cko-F-RAM+ n=12 (from 4 mice)		df=12 p=0.199				
		WT F-RAM- vs F-RAM+						P=0.003
		Nptx1 cko F-RAM- vs F-RAM+						P=0.000

		F-RAM- WT vs Nptx1 cko						P=0.415
		F-RAM+ WT vs Nptx1 cko						P=0.002
1o	Amplitude	WT-F-RAM- n=12 (from 4 mice)	neuron	df=12 p=0.102	df1=1 df2=23 p=0.749	Kruskal-Wallis H test	H=0.012	P=0.913
		Nptx2 cko-F-RAM- n=13 (from 4 mice)		df=13 p=0.123				
		WT-F-RAM+ n=12 (from 4 mice)		df=12 p=0.004	df1=1 df2=23 p=0.050		H=0.358	P=0.550
		Nptx2 cko-F-RAM+ n=13 (from 4 mice)		df=13 p=0.251				
		WT F-RAM- vs F-RAM+						P=0.013
		Nptx2 cko F-RAM- vs F-RAM+						P=0.006
		F-RAM- WT vs Nptx2 cko						P=0.913

		F-RAM+ WT vs Nptx2 cko						P=0.550
2e	Spike	WT n=22 (from 4 mice)	neuron	df=352 p=0.000	df1=1 df2=718 p=0.000	Kruskal-Wallis H test	H=42.841	P=0.000
		Nptx1 cko n=23 (from 4 mice)		df=368 p=0.000				
2f	RMP	WT n=22 (from 4 mice)	neuron	df=22 p=0.572	df1=1 df2=43 p=0.672	Two-tailed unpaired t-test	t=-3.348	P=0.002
		Nptx1 cko n=23 (from 4 mice)		df=23 p=0.667				
2g	Rheobase	WT n=22 (from 4 mice)	neuron	df=22 p=0.477	df1=1 df2=43 p=0.130	Two-tailed unpaired t-test	t=4.617	P=0.000
		Nptx1 cko n=23 (from 4 mice)		df=23 p=0.651				
2i	Spike	WT n=15	neuron	df=240 p=0.000	df1=1 df2=494 p=0.403	Kruskal-Wallis H test	H=0.274	P=0.601
		Nptx2 cko n=16		df=256 p=0.000				
2j	RMP	WT n=15 (from 4 mice)	neuron	df=15 p=0.275	df1=1 df2=29 p=0.685	Two-tailed unpaired t-test	t=-0.773	P=0.446

		Nptx2 cko n=16 (from 3 mice)		df=16 p=0.279				
2k	Rheobase	WT n=15 (from 4 mice)	neuron	df=15 p=0.992	df1=1 df2=29 p=0.305	Two-tailed unpaired t-test	t=-0.051	P=0.960
		Nptx2 cko n=16 (from 3 mice)		df=16 p=0.163				
2n	Intensity	WT n=53 (from 5 mice)	neuron	df=53 p=0.200 **(Kolmogorov-Smirnov)**	df1=1 df2=99 p=0.006	Two-tailed unpaired t-test	t=2.667	P=0.009
		Nptx1 cko n=48 (from 6 mice)		df=48 p=0.931				
2p	Intensity	WT n=38 (from 5 mice)	neuron	df=38 p=0.002	df1=1 df2=69 p=0.448	Mann-Whitney U test	Z=-0.427	P=0.670
		Nptx2 cko n=33 (from 6 mice)		df=33 p=0.240				
2r	Im	Im n=6	neuron (from 3 mice)	df=6 p=0.301		Two-tailed paired t-test	t=4.506	P=0.006
		Im+XE991 n=6						

2u	Im (pA)	WT-sgScramble n=14 (from 3 mice)	neuron	df=14 p=0.595	df1=1 df2=24 p=0.004	Two-way ANOVA	$F_{Treatment \times mice(1, 51)}=7.897$	P=0.007
		Nptx1 cko-sgScramble n=12 (from 3 mice)		df=12 p=0.555			$F_{mice(1, 51)}=6.665$	P=0.013
		WT-sgKcnq2 n=14 (from 3 mice)		df=14 p=0.200	df1=1 df2=27 p=0.154		$F_{Treatment(1, 51)}=19.927$	P=0.000
		Nptx1 cko-sgKcnq2 n=15 (from 3 mice)		df=15 p=0.964				
		sgScramble WT vs Nptx1 cko						P=0.001
		WT sgScramble vs sgKcnq2						P=0.000
3b	(dsRed+PV)/PV	F-RAM n=4	mouse	df=4 p=0.450	df1=1 df2=7 p=0.446	Two-tailed unpaired t-test	t=-5.910	P=0.001
		N-RAM n=5		df=5 p=0.785				

3f	A/N	WT n=13 (from 4 mice)	neuron	df=13 p=0.109	df1=1 df2=24 p=0.877	Two-tailed unpaired t-test	t=-0.192	P=0.849
		Nptx1 cko n=13 (from 4 mice)		df=13 p=0.230				
3h	A/N	WT n=16 (from 3 mice)	neuron	df=16 p=0.690	df1=1 df2=31 p=0.165	Two-tailed unpaired t-test	t=3.925	P=0.001
		Nptx2 cko n=17 (from 5 mice)		df=17 p=0.896				
3i	Amplitude	WT n=16 (from 3 mice)	neuron	df=16 p=0.270	df1=1 df2=31 p=0.007	Two-tailed unpaired t-test	t=2.571	P=0.015
		Nptx2 cko n=17 (from 5 mice)		df=17 p=0.331				
3j	Amplitude	WT n=16 (from 3 mice)	neuron	df=16 p=0.118	df1=1 df2=31 p=0.240	Two-tailed unpaired t-test	t=0.202	P=0.841
		Nptx2 cko n=17 (from 5 mice)		df=17 p=0.879				
3l	Intensity	WT n=32 (from 5	neuron	df=32 p=0.000	df1=1 df2=67 p=0.034	Mann-Whitney U	Z=-4.272	P=0.000

		mice)				test		
		Nptx2 cko n=37 (from 5 mice)		df=37 p=0.029				
3p	PPR	Scramble n=21 (from 4 mice)	neuron	df=21 p=0.647	df1=1 df2=41 p=0.004	Mann-Whitney U test	Z=-3.280	P=0.001
		Nptx2 sh n=22 (from 4 mice)		df=22 p=0.006				
3r	Amplitude	Scramble n=25 (from 4 mice)	neuron	df=25 p=0.054	df1=1 df2=47 p=0.221	Mann-Whitney U test	Z=-2.160	P=0.031
		Nptx2 sh n=24 (from 4 mice)		df=24 p=0.004				
4d	Freezing (%)	WT context A n=14	mouse	df=14 p=0.182	df1=1 df2=23 p=0.249	Two-way RM ANOVA	$F_{Treatment \times context(1, 23)}=1.826$	P=0.190
		Nptx1 cko context A n=11		df=11 p=0.432			$F_{context(1, 23)}=120.178$	P=0.000
		WT context C n=14		df=14 p=0.415	df1=1 df2=23 p=0.546		$F_{Treatment(1, 23)}=3.760$	P=0.065

		Nptx1 context C n=11		df=11 p=0.061				
		WT vs Nptx1 cko context A						P=0.009
		WT vs Nptx1 cko context C						P=0.389
4e	Discrimination index	WT n=14	mouse	df=14 p=0.241	df1=1 df2=23 p=0.891	Two-tailed unpaired t-test	t=0.090	P=0.929
		Nptx1 cko n=11		df=11 p=0.165				
4f	Freezing (%)	WT context A n=14	mouse	df=14 p=0.441	df1=1 df2=25 p=0.813	Two-way RM ANOVA	$F_{Treatment \times context(1, 25)}=0.071$	P=0.792
		Nptx2 cko context A n=13		df=13 p=0.405			$F_{context(1, 25)}=216.928$	P=0.000
		WT context C n=14		df=14 p=0.177	df1=1 df2=25 p=0.228		$F_{Treatment(1, 25)}=0.334$	P=0.568
		Nptx2 context C n=13		df=13 p=0.382				
		WT vs Nptx2 cko context						P=0.550

		A						
		WT vs Nptx2 cko context C						P=0.663
4g	Discrimination index	WT n=14	mouse	df=14 p=0.094	df1=1 df2=25 p=0.546	Mann-Whitney U test	Z=-0.437	P=0.684
		Nptx2 cko n=13		df=13 p=0.028				
4k	Freezing (%)	WT context A n=18	mouse	df=18 p=0.630	df1=1 df2=31 p=0.805	Two-way RM ANOVA	$F_{Treatment \times context(1, 31)}=0.370$	P=0.547
		Nptx1 cko context A n=15		df=15 p=0.434			$F_{context(1, 31)}=222.450$	P=0.000
		WT context C n=18		df=18 p=0.268	df1=1 df2=31 p=0.900		$F_{Treatment(1, 31)}=0.423$	P=0.520
		Nptx1 context C n=15		df=15 p=0.542				
		WT vs Nptx1 cko context A						P=0.683
		WT vs Nptx1 cko context C						P=0.416

4l	Discrimination index	WT n=18	mouse	df=18 p=0.588	df1=1 df2=31 p=0.825	Mann-Whitney U test	Z=-1.048	P=0.307
		Nptx1 cko n=15		df=15 p=0.027				
4m	Freezing (%)	WT context A n=13	mouse	df=13 p=0.199	df1=1 df2=23 p=0.642	Two-way RM ANOVA	$F_{Treatment \times context(1, 23)}=5.607$	P=0.027
		Nptx2 cko context A n=12		df=12 p=0.905			$F_{context(1, 23)}=24.994$	P=0.000
		WT context C n=13		df=13 p=0.598	df1=1 df2=23 p=0.547		$F_{Treatment(1, 23)}=2.321$	P=0.141
		Nptx2 context C n=12		df=12 p=1.000				
		WT vs Nptx2 cko context A						P=0.960
		WT vs Nptx2 cko context C						P=0.016
4n	Discrimination index	WT n=13	mouse	df=13 p=0.048	df1=1 df2=23 p=0.930	Mann-Whitney U test	Z=-2.502	P=0.011
		Nptx2 cko n=12		df=12 p=0.741				

4r	Freezing (%)	WT context A n=11	mouse	df=11 p=0.499	df1=1 df2=20 p=0.179	Two-way RM ANOVA	$F_{Treatment \times context(1, 20)}=0.545$	P=0.469
		Nptx1 cko context A n=11		df=11 p=0.816			$F_{context(1, 20)}=66.734$	P=0.000
		WT context C n=11		df=11 p=0.253	df1=1 df2=20 p=0.825		$F_{Treatment(1, 20)}=0.135$	P=0.717
		Nptx1 context C n=11		df=11 p=0.261				
		WT vs Nptx1 cko context A						P=0.977
		WT vs Nptx1 cko context C						P=0.537
4s	Discrimination index	WT n=11	mouse	df=11 p=0.631	df1=1 df2=20 p=0.898	Two-tailed unpaired t-test	t=0.652	P=0.522
		Nptx1 cko n=11		df=11 p=0.269				
4t	Freezing (%)	WT context A n=10	mouse	df=10 p=0.188	df1=1 df2=17 p=0.932	Two-way RM ANOVA	$F_{Treatment \times context(1, 17)}=0.964$	P=0.340
		Nptx2 cko context A		df=9 p=0.790			$F_{context(1, 17)}=51.810$	P=0.000

		n=9						
		WT context C n=10		df=10 p=0.461	df1=1 df2=17 p=0.425		$F_{Treatment(1, 17)}$=0.053	P=0.820
		Nptx2 context C n=9		df=9 p=0.436				
		WT vs Nptx2 cko context A						P=0.497
		WT vs Nptx2 cko context C						P=0.784
4u	Discrimination index	WT n=10	mouse	df=10 p=0.541	df1=1 df2=17 p=0.904	Two-tailed unpaired t-test	t=0.436	P=0.669
		Nptx2 cko n=9		df=9 p=0.874				
5d	Freezing (%)	WT-vehicle context A n=12	mouse	df=12 p=0.398	df1=3 df2=43 p=0.369	Two-way RM ANOVA	$F_{Treatment \times context(3, 43)}$=0.800	P=0.500
		WT-retigabine context A n=13		df=13 p=0.399			$F_{context(1, 43)}$=109.902	P=0.000

		Nptx1 cko-vehicle context A n=10		df=10 p=0.086			$F_{Treatment(3, 43)}=4.114$	P=0.012
		Nptx1 cko-retigabine context A n=12		df=12 p=0.126				
		WT-vehicle context C n=12		df=12 p=0.489	df1=3 df2=43 p=0.003			
		WT-retigabine context C n=13		df=13 p=0.173				
		Nptx1 cko-vehicle context C n=10		df=10 p=0.351				
		Nptx1 cko-retigabine context C n=12		df=12 p=0.446				

		context A WT-vehicle vs Nptx1 cko vehicle						P=0.021
		context A Nptx1 cko vehicle vs Nptx1 cko retigabine						P=0.009
		context C WT-vehicle vs Nptx1 cko vehicle						P=0.541
		context C Nptx1 cko vehicle vs Nptx1 cko retigabine						P=0.507
5e	Discrimination index	WT-vehicle n=12	mouse	df=12 p=0.748	df1=3 df2=43 p=0.523	One-way ANOVA	$F_{Treatment \times context(3, 43)}=0.033$	P=0.992
		WT-retigabine n=13		df=13 p=0.171				
		Nptx1 cko-vehicle n=10		df=10 p=0.788				

		Nptx1 cko-retigabine n=12		df=12 p=0.367				
5f	Freezing (%)	WT-vehicle context A n=10	mouse	df=10 p=0.997	df1=3 df2=38 p=0.336	Two-way RM ANOVA	$F_{Treatment \times context(3, 38)}=1.010$	P=0.399
		WT-retigabine context A n=11		df=11 p=0.727			$F_{context(1, 38)}=233.119$	P=0.000
		Nptx1 cko-vehicle context A n=11		df=11 p=0.092			$F_{Treatment(3, 38)}=0.044$	P=0.988
		Nptx1 cko-retigabine context A n=10		df=10 p=0.472				
		WT-vehicle context C n=10		df=10 p=0.126	df1=3 df2=43 p=0.108			
		WT-retigabine context C n=11		df=11 p=0.293				

		Nptx1 cko-vehicle context C n=11		df=11 p=0.626				
		Nptx1 cko-retigabine context C n=10		df=10 p=0.602				
		context A WT-vehicle vs Nptx1 cko vehicle						P=1.000
		context A Nptx1 cko vehicle vs Nptx1 cko retigabine						P=1.000
		context C WT-vehicle vs Nptx1 cko vehicle						P=1.000
		context C Nptx1 cko vehicle vs Nptx1 cko retigabine						P=1.000

5g	Discrimination index	WT-vehicle n=10	mouse	df=10 p=0.155	df1=3 df2=38 p=0.032	Kruskal-Wallis H test	H=0.751	P=0.861
		WT-retigabine n=11		df=11 p=0.877				
		Nptx1 cko-vehicle n=11		df=11 p=0.036				
		Nptx1 cko-retigabine n=10		df=10 p=0.072				
5k	Freezing (%)	Scramble-mcherry context A n=10	mouse	df=10 p=0.425	df1=3 df2=39 p=0.712	Two-way RM ANOVA	$F_{Treatment \times context(3, 39)}=0.493$	P=0.689
		Scramble-hM3Dq context A n=11		df=11 p=0.197			$F_{context(1, 39)}=96.614$	P=0.000
		Nptx2 sh-mcherry context A n=10		df=10 p=0.146			$F_{Treatment(3, 39)}=0.014$	P=0.996
		Nptx2 sh-hM3Dq context A n=12		df=12 p=0.620				

		Scramble-mcherry context C n=10		df=10 p=0.649	df1=3 df2=39 p=0.113			
		Scramble-hM3Dq context C n=11		df=11 p=0.979				
		Nptx2 sh-mcherry context C n=10		df=10 p=0.306				
		Nptx2 sh-hM3Dq context C n=12		df=12 p=0.838				
		context A scramble-mcherry vs Nptx2 sh-mcherry						P=1.000
		context A Nptx2 sh-mcherry vs Nptx2 sh-hM3Dq						P=1.000

		context C scramble-mcherry vs Nptx2 sh-mcherry						P=1.000
		context C Nptx2 sh-mcherry vs Nptx2 sh-hM3Dq						P=1.000
5l	Discrimination index	Scramble-mcherry n=12	mouse	df=10 p=0.458	df1=3 df2=39 p=0.797	One-way ANOVA	$F_{Treatment \times context(3, 39)}$=0.149	P=0.930
		Scramble-hM3Dq n=10		df=11 p=0.373				
		Nptx2 sh-mcherry n=13		df=10 p=0.103				
		Nptx2 sh-hM3Dq n=10		df=12 p=0.085				
5m	Freezing (%)	Scramble-mcherry context A n=16	mouse	df=16 p=0.739	df1=3 df2=56 p=0.382	Kruskal-Wallis H test	H=0.1.863	P=0.601

		Scramble-hM3Dq context A n=15		df=15 p=0.181				
		Nptx2 sh-mcherry context A n=15		df=15 p=0.212				
		Nptx2 sh-hM3Dq context A n=14		df=14 p=0.009				
		Scramble-mcherry context C n=16		df=16 p=0.228	df1=3 df2=56 p=0.030		H=11.685	P=0.009
		Scramble-hM3Dq context C n=15		df=15 p=0.500				
		Nptx2 sh-mcherry context C n=15		df=15 p=0.768				

		Nptx2 sh-hM3Dq context C n=14		df=14 p=0.125				
		context C scramble-mcherry vs Nptx2 sh-mcherry						P=0.020
		context C Nptx2 sh-mcherry vs Nptx2 sh-hM3Dq						P=0.151
5n	Discrimination index	Scramble-mcherry n=16	mouse	df=16 p=0.135	df1=3 df2=56 p=0.087	One-way ANOVA	$F_{Treatment \times context(3, 39)}=8.188$	P=0.000
		Scramble-hM3Dq n=15		df=15 p=0.380				
		Nptx2 sh-mcherry n=15		df=15 p=0.586				
		Nptx2 sh-hM3Dq n=14		df=14 p=0.159				

		Scramble-mcherry vs Nptx2 sh-mcherry						P=0.001
		Nptx2 sh-mcherry vs Nptx2 sh-hM3Dq						P=0.007
6f	Cell number	Young HC n=4	mouse	df=4 p=0.241	df1=1 df2=7 p=0.457	Two-way ANOVA	$F_{mice \times time(4, 35)}$=3.881	P=0.010
		Aged HC n=5		df=5 p=0.073			$F_{time(4, 35)}$=14.402	P=0.000
		Young 15min n=5		df=5 p=0.521	df1=1 df2=8 p=0.313		$F_{mice(1, 35)}$=20.273	P=0.000
		Aged 15min n=5		df=5 p=0.612				
		Young 30min n=4		df=4 p=0.960	df1=1 df2=6 p=0.181			
		Aged 30min n=4		df=4 p=0.272				
		Young 60min n=4		df=4 p=0.636	df1=1 df2=7 p=0.688			
		Aged 60min n=5		df=5 p=0.105				

		Young 120min n=4		df=4 p=0.809	df1=1 df2=7 p=0.510			
		Aged 120min n=5		df=5 p=0.108				
		Young HC vs 15min						P=0.002
		Young HC vs 30min						P=0.000
		Young HC vs 60min						P=0.032
		30min Young vs Aged						P=0.000
		60min Young vs Aged						P=0.003
6g	Cell number	Young HC n=4	mouse	df=4 p=0.111	df1=1 df2=7 p=0.629	Two-way ANOVA	$F_{mice \times time(4, 35)}$=5.535	P=0.001
		Aged HC n=5		df=5 p=0.489			$F_{time(4, 35)}$=15.299	P=0.000
		Young 15min n=5		df=5 p=0.932	df1=1 df2=8 p=0.720		$F_{mice(1, 35)}$=13.771	P=0.001
		Aged 15min n=5		df=5 p=0.555				

		Young 30min n=4		df=4 p=0.422	df1=1 df2=6 p=0.519			
		Aged 30min n=4		df=4 p=0.674				
		Young 60min n=4		df=4 p=0.225	df1=1 df2=7 p=0.724			
		Aged 60min n=5		df=5 p=0.078				
		Young 120min n=4		df=4 p=0.073	df1=1 df2=7 p=0.010			
		Aged 120min n=5		df=5 p=0.370				
		Young HC vs 15min						P=0.045
		Young HC vs 30min						P=0.000
		Young HC vs 60min						P=0.021
		30min Young vs Aged						P=0.000
		60min Young vs Aged						P=0.003

6h	Intensity	Young HC n=4	mouse	df=4 p=0.527	df1=1 df2=6 p=0.279	Two-way ANOVA	$F_{Treatment \times time(4, 36)}=0.544$	P=0.705
		Aged HC n=4		df=4 p=0.822			$F_{time(4, 36)}=8.148$	P=0.000
		Young 15min n=5		df=5 p=0.652	df1=1 df2=8 p=0.875		$F_{mice(1, 36)}=1.833$	P=0.184
		Aged 15min n=5		df=5 p=0.917				
		Young 30min n=5		df=5 p=0.376	df1=1 df2=7 p=0.004			
		Aged 30min n=4		df=4 p=0.263				
		Young 60min n=5		df=5 p=0.289	df1=1 df2=8 p=0.248			
		Aged 60min n=5		df=5 p=0.699				
		Young 120min n=4		df=4 p=0.570	df1=1 df2=7 p=0.935			
		Aged 120min n=5		df=5 p=0.481				
		Young HC vs 60min						P=0.006
		Aged HC vs 60min						P=0.013

6i	Intensity	Young HC n=4	mouse	df=4 p=0.326	df1=1 df2=6 p=0.874	Two-way ANOVA	$F_{mice\times time(4,36)}$=0.699	P=0.597
		Aged HC n=4		df=4 p=0.957			$F_{time(4,36)}$=7.052	P=0.000
		Young 15min n=5		df=5 p=0.066	df1=1 df2=8 p=0.360		$F_{mice(1,36)}$=12.441	P=0.001
		Aged 15min n=5		df=5 p=0.847				
		Young 30min n=5		df=5 p=0.278	df1=1 df2=7 p=0.225			
		Aged 30min n=4		df=4 p=0.563				
		Young 60min n=5		df=5 p=0.874	df1=1 df2=8 p=0.721			
		Aged 60min n=5		df=5 p=0.632				
		Young 120min n=4		df=4 p=0.990	df1=1 df2=7 p=0.281			
		Aged 120min n=5		df=5 p=0.715				
		Young HC vs 60min						P=0.010
		15min Young vs Aged						P=0.026

		60min Young vs Aged						P=0.019
6j	Intensity	Young HC n=5	mouse	df=5 p=0.505	df1=1 df2=7 p=0.092	Two-way ANOVA	$F_{mice \times time(4, 36)}$=1.455	P=0.237
		Aged HC n=4		df=4 p=0.102			$F_{time(4, 34)}$=10.314	P=0.000
		Young 15min n=5		df=5 p=0.313	df1=1 df2=7 p=0.771		$F_{mice(1, 34)}$=1.796	P=0.013
		Aged 15min n=4		df=4 p=0.847				
		Young 30min n=5		df=5 p=0.786	df1=1 df2=7 p=0.025			
		Aged 30min n=4		df=4 p=0.604				
		Young 60min n=5		df=5 p=0.307	df1=1 df2=7 p=0.314			
		Aged 60min n=4		df=4 p=0.924				
		Young 120min n=3		df=3 p=0.393	df1=1 df2=6 p=0.691			
		Aged 120min n=5		df=5 p=0.854				
		Young HC vs 60min						P=0.000

		60min Young vs Aged						P=0.003
6k	Cell number	Young HC n=5	mouse	df=5 p=0.363	df1=1 df2=7 p=0.664	Two-way ANOVA	$F_{mice \times time(4, 36)}$=2.739	P=0.044
		Aged HC n=4		df=4 p=0.214			$F_{time(4, 36)}$=9.458	P=0.000
		Young 15min n=4		df=4 p=0.353	df1=1 df2=7 p=0.476		$F_{mice(1, 36)}$=23.494	P=0.000
		Aged 15min n=5		df=5 p=0.398				
		Young 30min n=5		df=5 p=0.499	df1=1 df2=7 p=0.838			
		Aged 30min n=4		df=4 p=0.250				
		Young 60min n=5		df=5 p=0.168	df1=1 df2=8 p=0.699			
		Aged 60min n=5		df=5 p=0.683				
		Young 120min n=5		df=4 p=0.758	df1=1 df2=7 p=0.341			
		Aged 120min n=4		df=5 p=0.178				
		Young HC vs 15min						P=0.000

		Young HC vs 30min						P=0.000
		15min Young vs Aged						P=0.000
		30min Young vs Aged						P=0.012
		60min Young vs Aged						P=0.018
6I	Intensity	Young HC n=4	mouse	df=4 p=0.846	df1=1 df2=6 p=0.274	Two-way ANOVA	$F_{mice \times time(4, 35)}$=0.360	P=0.835
		Aged HC n=4		df=4 p=0.127			$F_{time(4, 35)}$=7.168	P=0.000
		Young 15min n=5		df=5 p=0.730	df1=1 df2=7 p=0.193		$F_{mice(1, 35)}$=26.426	P=0.000
		Aged 15min n=4		df=4 p=0.055				
		Young 30min n=6		df=6 p=0.452	df1=1 df2=8 p=0.009			
		Aged 30min n=4		df=4 p=0.887				
		Young 60min n=5		df=5 p=0.418	df1=1 df2=7 p=0.936			

		Aged 60min n=4		df=4 p=0.353				
		Young 120min n=5		df=5 p=0.130	df1=1 df2=7 p=0.028			
		Aged 120min n=4		df=4 p=0.535				
		Young HC vs 120min						P=0.006
		15min Young vs Aged						P=0.031
		30min Young vs Aged						P=0.049
		60min Young vs Aged						P=0.002
6m	Intensity	Young HC n=4	mouse	df=4 p=0.816	df1=1 df2=6 p=0.333	Two-way ANOVA	$F_{mice \times time(4, 33)}=0.406$	P=0.803
		Aged HC n=4		df=4 p=0.320			$F_{time(4, 33)}=7.555$	P=0.000
		Young 15min n=5		df=5 p=0.503	df1=1 df2=7 p=0.176		$F_{mice(1, 33)}=29.404$	P=0.000
		Aged 15min n=4		df=4 p=0.077				

		Young 30min n=5		df=5 p=0.785	df1=1 df2=7 p=0.029			
		Aged 30min n=4		df=4 p=0.499				
		Young 60min n=5		df=5 p=0.883	df1=1 df2=7 p=0.734			
		Aged 60min n=4		df=4 p=0.340				
		Young 120min n=4		df=4 p=0.943	df1=1 df2=6 p=0.457			
		Aged 120min n=4		df=4 p=0.458				
		Young HC vs 120min						P=0.009
		HC Young vs Aged						P=0.041
		15min Young vs Aged						P=0.036
		60min Young vs Aged						P=0.001
		120min Young vs Aged						P=0.025

6p	Cell number	Young F-RAM n=6	mouse	df=6 p=0.316	df1=1 df2=11 p=0.388	Two-way ANOVA	$F_{treatment\ x\ mice(1, 18)}$=0.007	P=0.933
		Young N-RAM n=7		df=7 p=0.767			$F_{mice(1, 18)}$=121.219	P=0.000
		Aged F-RAM n=5		df=5 p=0.837	df1=1 df2=7 p=0.897		$F_{Treatment(1, 18)}$=0.001	P=0.977
		Aged N-RAM n=4		df=4 p=0.358				
		F-RAM young vs aged						P=0.000
		N-RAM young vs aged						P=0.000
7c	Freezing (%)	Young context A n=11	mouse	df=11 p=0.114	df1=1 df2=19 p=0.029	Two-way RM ANOVA	$F_{mice\ \times\ context(1, 19)}$=1.285	P=0.271
		Aged context A n=10		df=10 p=0.541			$F_{context(1, 19)}$=14.700	P=0.001
		Young context C n=11		df=11 p=0.873	df1=1 df2=19 p=0.310		$F_{mice(1, 19)}$=3.378	P=0.082

		Aged context C n=10		df=10 p=0.448				
		context A Young vs Aged						P=0.029
		context C Young vs Aged						P=0.256
		Young context A vs context C						P=0.002
		Aged context A vs context C						P=0.078
7d	Discrimination index	Young n=11	mouse	df=11 p=0.025	df1=1 df2=19 p=0.388	Mann-Whitney U test	Z=-0.282	P=0.778
		Aged n=10		df=10 p=0.433				
7e	Freezing (%)	Young context A n=12	mouse	df=12 p=0.922	df1=1 df2=21 p=0.439	Two-way RM ANOVA	$F_{mice \times context(1, 21)}=4.055$	P=0.057
		Aged context A n=11		df=11 p=0.264			$F_{context(1, 21)}=81.119$	P=0.000
		Young context C		df=12 p=0.712	df1=1 df2=21 p=0.859		$F_{mice(1, 21)}=2.625$	P=0.120

		n=12						
		Aged context C n=11		df=11 p=0.393				
		context A Young vs Aged						P=0.491
		context C Young vs Aged						P=0.025
		Young context A vs context C						P=0.000
		Aged context A vs context C						P=0.000
7f	Discrimination index	Young n=12	mouse	df=12 p=0.813	df1=1 df2=21 p=0.040	Two-tailed unpaired t-test	t=2.941	P=0.008
		Aged n=11		df=11 p=0.105				
7j	Im (pA)	Young n=14 (from 3 mice)	neuron	df=14 p=0.346	df1=1 df2=27 p=0.103	Two-tailed unpaired t-test	t=3.013	P=0.006
		Aged n=15 (from 3 mice)		df=15 p=0.456				

7l	Intensity	Young n=88 (from 6 mice)	neuron	df=88 p=0.008 **(Kolmogorov-Smirnov)**	df1=1 df2=150 p=0.000	Mann-Whitney U test	Z=-4.090	P=0.000
		Aged cko n=64 (from 6 mice)		df=64 p=0.002 **(Kolmogorov-Smirnov)**				
7n	Relative protein quantification	Young n=4	mouse	df=4 p=0.943	df1=1 df2=7 p=0.502	Two-tailed unpaired t-test	t=2.609	P=0.035
		Aged n=5		df=5 p=0.214				
7r	Freezing (%)	EYFP context A n=11	mouse	df=11 p=0.892	df1=1 df2=21 p=0.269	Two-way RM ANOVA	$F_{Treatment \times context(1, 21)}=0.730$	P=0.403
		Nptx1 context A n=12		df=12 p=0.484			$F_{context(1, 21)}=4.839$	P=0.039
		EYFP context C n=11		df=11 p=0.606	df1=1 df2=21 p=0.603		$F_{Treatment(1, 21)}=3.605$	P=0.044
		Nptx1 context C n=12		df=12 p=0.454				
		EYFP vs Nptx1 context A						P=0.043

		EYFP vs Nptx1 context C						P=0.291
7s	Discrimination index	EYFP n=11	mouse	df=11 p=0.405	df1=1 df2=21 p=0.854	Two-tailed unpaired t-test	t=-0.095	P=0.925
		Nptx1 n=12		df=12 p=0.457				
7t	Freezing (%)	EYFP context A n=14	mouse	df=14 p=0.892	df1=1 df2=21 p=0.269	Two-way RM ANOVA	$F_{Treatment \times context(1, 21)}$=0.051	P=0.823
		Nptx1 context A n=9		df=9 p=0.484			$F_{context(1, 21)}$=7.110	P=0.014
		EYFP context C n=14		df=14 p=0.606	df1=1 df2=21 p=0.603		$F_{Treatment(1, 21)}$=0.312	P=0.582
		Nptx1 context C n=9		df=9 p=0.454				
		EYFP vs Nptx1 context A						P=0.547
		EYFP vs Nptx1 context C						P=0.672
7u	Discrimination index	EYFP n=14	mouse	df=14 p=0.898	df1=1 df2=21 p=0.708	Two-tailed unpaired t-	t=-0.118	P=0.907
		Nptx1 n=9		df=9 p=0.924				

						test		
8e	A/N	Young n=9 (from 3 mice)	neuron	df=9 p=0.041	df1=1 df2=17 p=0.226	Mann-Whitney U test	Z=-2.252	P=0.024
		Aged n=9 (from 3 mice)		df=9 p=0.503				
8f	Amplitude (pA)	Young n=9 (from 3 mice)	neuron	df=9 p=0.937	df1=1 df2=16 p=0.089	Two-tailed unpaired t-test	t=2.644	P=0.018
		Aged n=9 (from 3 mice)		df=9 p=0.747				
8g	Amplitude (pA)	Young n=9 (from 3 mice)	neuron	df=9 p=0.333	df1=1 df2=16 p=0.085	Two-tailed unpaired t-test	t=0.796	P=0.437
		Aged n=9 (from 3 mice)		df=9 p=0.950				
8i	Intensity	Young n=28 (from 4 mice)	neuron	df=28 p=0.069	df1=1 df2=49 p=0.353	Two-tailed unpaired t-test	t=4.637	P=0.000
		Aged n=23 (from 5 mice)		df=23 p=0.134				

8k	Intensity	Young n=160 (from 6 mice)	neuron	df=160 p=0.200 **(Kolmogorov-Smirnov)**	df1=1 df2=252 p=0.087	Mann-Whitney U test	Z=-5.388	P=0.000
		Aged n=94 (from 6 mice)		df=94 p=0.001 **(Kolmogorov-Smirnov)**				
8o	Freezing (%)	EGFP context A n=11	mouse	df=11 p=0.628	df1=1 df2=20 p=0.932	Two-way RM ANOVA	$F_{Treatment \times context(1, 20)}=0.555$	P=0.645
		Nptx2-PTX context A n=11		df=11 p=0.613			$F_{context(1, 20)}=36.863$	P=0.000
		EGFP context C n=11		df=11 p=0.767	df1=1 df2=20 p=0.164		$F_{Treatment(1, 20)}=0.138$	P=0.765
		Nptx2-PTX context C n=11		df=11 p=0.116				
		EGFP vs Nptx2-PTX context A						P=0.953
		EGFP vs Nptx2-PTX context C						P=0.516
8p	Discrimination index	EGFP n=11	mouse	df=11 p=0.450	df1=1 df2=20 p=0.450	Two-tailed	t=-0.251	P=0.804

		Nptx2-PTX n=11		df=11 p=0.794		unpaired t-test		
8q	Freezing (%)	EGFP context A n=13	mouse	df=13 p=0.266	df1=1 df2=21 p=0.021	Two-way RM ANOVA	$F_{Treatment \times context(1, 21)}=4.715$	P=0.041
		Nptx2-PTX context A n=10		df=10 p=0.766			$F_{context(1, 21)}=95.605$	P=0.000
		EGFP context C n=13		df=13 p=0.140	df1=1 df2=21 p=0.515		$F_{Treatment(1, 21)}=0.376$	P=0.546
		Nptx2-PTX context C n=10		df=10 p=0.651				
		EGFP vs Nptx2-PTX context A						P=0.671
		EGFP vs Nptx2-PTX context C						P=0.043
8r	Discrimination index	EGFP n=13	mouse	df=13 p=0.052	df1=1 df2=20 p=0.358	Two-tailed unpaired t-test	t=0.358	P=0.006
		Nptx2-PTX n=10		df=10 p=0.486				
S1d	Cell number	corn oil n=4	mouse	df=4 p=0.039	df1=1 df2=6 p=0.799	Mann-Whitney U	Z=-2.309	P=0.029
		4-OHT n=4		df=4 p=0.616				

						test		
S1g	EGFP+mKate2/mKate2	F-RAM n=5	mouse	df=5 p=0.715	df1=1 df2=7 p=0.002	Two-tailed unpaired t-test	t=-5.118	P=0.013
		N-RAM n=4		df=4 p=0.654				
S2c-Cell number	F-RAM On dox-HC n=4	mouse	df=4 p=0.432	df1=1 df2=6 p=0.760	Kruskal-Wallis H test	H=0.083	P=0.773	
	N-RAM On dox-HC n=4		df=4 p=0.412					
	F-RAM On dox-CFC n=4		df=4 p=0.012	df1=1 df2=6 p=0.629		H=0.083	P=0.773	
	N-RAM On dox-CFC n=4		df=4 p=0.044					
	F-RAM Off dox-HC n=4		df=4 p=0.338	df1=1 df2=6 p=0.116		H=0.000	P=1.000	
	N-RAM Off dox-HC n=4		df=4 p=0.165					
	F-RAM Off dox-CFC n=4		df=4 p=0.212	df1=1 df2=6 p=0.887		H=0.000	P=1.000	
	N-RAM Off dox-CFC n=4		df=4 p=0.992					
	F-RAM On dox-HC vs On Dox-CFC						P=1.000	
	F-RAM On dox-HC vs Off Dox-HC						P=0.321	
	F-RAM On dox-HC vs Off Dox-CFC						P=0.011	

	F-RAM On dox-CFC vs Off Dox-CFC						P=0.029	
	F-RAM On dox-CFC vs Off Dox-HC						P=0.614	
	F-RAM Off dox-HC vs Off Dox-CFC						P=1.000	
	N-RAM On dox-HC vs On Dox-CFC						P=1.000	
	N-RAM On dox-HC vs Off Dox-HC						P=0.321	
	N-RAM On dox-HC vs Off Dox-CFC						P=0.011	
	N-RAM On dox-CFC vs Off Dox-CFC						P=0.029	
	N-RAM On dox-CFC vs Off Dox-HC						P=0.614	
	N-RAM Off dox-HC vs Off Dox-CFC						P=1.000	
S3e	Relative mRNA expression	WT n=5	mouse	df=5 p=0.622	df1=1 df2=7 p=0.062	Two-tailed unpaired t-test	t=3.602	P=0.009
		Nptx1 cko n=4		df=4 p=0.798				
S3f	Relative mRNA expression	WT n=5	mouse	df=5 p=0.862	df1=1 df2=7 p=0.119	Two-tailed unpaired t-test	t=0.547	P=0.601
		Nptx1 cko n=4		df=4 p=0.228				

S3g	Relative mRNA expression	WT n=5	mouse	df=5 p=0.152	df1=1 df2=7 p=0.317	Two-tailed unpaired t-test	t=2.888	P=0.023
		Nptx1 cko n=4		df=4 p=0.842				
S3h	Relative mRNA expression	WT n=5	mouse	df=5 p=0.007	df1=1 df2=7 p=0.433	Mann-Whitney U test	Z=-0.245	P=0.806
		Nptx1 cko n=4		df=4 p=0.547				
S3i	Relative mRNA expression	WT n=4	mouse	df=4 p=0.447	df1=1 df2=7 p=0.021	Two-tailed unpaired t-test	t=4.803	P=0.002
		Nptx2 cko n=5		df=5 p=0.407				
S3j	Relative mRNA expression	WT n=4	mouse	df=4 p=0.061	df1=1 df2=7 p=0.519	Two-tailed unpaired t-test	t=0.024	P=0.982
		Nptx2 cko n=5		df=5 p=0.395				
S3k	Relative mRNA expression	WT n=5	mouse	df=5 p=0.825	df1=1 df2=8 p=0.188	Two-tailed unpaired t-test	t=7.722	P=0.000
		Nptx2 cko n=5		df=5 p=0.073				
S3l	Relative mRNA expression	WT n=5	mouse	df=5 p=0.739	df1=1 df2=8 p=0.172	Two-tailed unpaired t-test	t=-0.552	P=0.596
		Nptx2 cko n=5		df=5 p=0.819				
S4f	Intensity	WT 1.5h n=4	mouse	df=4 p=0.696	df1=1 df2=6 p=0.137	Two-way ANOVA	$F_{time\ x\ mice(3, 20)}$=2.883	P=0.061
		Nptx1 cKO 1.5h n=4		df=4 p=0.096			$F_{mice(1, 20)}$=11.718	P=0.003
		WT 6h n=3		df=3 p=0.672	df1=1 df2=5 p=0.904		$F_{time(1, 20)}$=8.984	P=0.001

		Nptx1 cKO 6h n=4		df=4 p=0.280				
		WT 24h n=3		df=3 p=0.904	df1=1 df2=4 p=0.139			
		Nptx1 cKO 24h n=3		df=3 p=0.886				
		WT 72h n=3		df=3 p=0.558	df1=1 df2=5 p=0.197			
		Nptx1 cKO 72h n=4		df=4 p=0.225				
		1.5h WT vs Nptx1 cKO						P=0.561
		6h WT vs Nptx1 cKO						P=0.981
		24h WT vs Nptx1 cKO						P=0.011
		72h WT vs Nptx1 cKO						P=0.019
		WT 1.5h vs 6h						P=0.614
		WT 1.5h vs 24h						P=0.999
		WT 1.5h vs 72h						P=0.474
		Nptx1 cKO 1.5h vs 6h						P=1.000
		Nptx1 cKO 1.5h vs 24h						P=0.010

		Nptx1 cKO 1.5h vs 72h						P=0.000
S4g	Intensity	WT 1.5h n=4	mouse	df=4 p=0.317	df1=1 df2=5 p=0.432	Kruskal-Wallis H test	H=0.0125	P=0.724
		Nptx1 cKO 1.5h n=3		df=3 p=0.817				
		WT 6h n=3		df=3 p=0.107	df1=1 df2=5 p=0.051		H=0.500	P=0.480
		Nptx1 cKO 6h n=4		df=4 p=0.120				
		WT 24h n=3		df=3 p=0.021	df1=1 df2=4 p=0.079		H=3.857	P=0.050
		Nptx1 cKO 24h n=3		df=3 p=0.505				
		WT 72h n=3		df=3 p=0.963	df1=1 df2=5 p=0.420		H=4.500	P=0.034
		Nptx1 cKO 72h n=4		df=4 p=0.370				
		1.5h WT vs Nptx1 cKO						P=0.724
		6h WT vs Nptx1 cKO						P=0.480
		24h WT vs Nptx1 cKO						P=0.050
		72h WT vs Nptx1 cKO						P=0.034
		WT 1.5h vs 6h						P=0.480
		WT 1.5h vs 24h						P=0.289

		WT 1.5h vs 72h						P=0.289
		Nptx1 cKO 1.5h vs 6h						P=0.034
		Nptx1 cKO 1.5h vs 24h						P=0.050
		Nptx1 cKO 1.5h vs 72h						P=0.034
S5f	Intensity	WT 1.5h n=4	mouse	df=4 p=0.322	df1=1 df2=6 p=0.053	Two-way ANOVA	$F_{time\ x\ mice(3,21)}$=6.660	P=0.002
		Nptx2 cKO 1.5h n=4		df=4 p=0.544			$F_{mice(1,21)}$=22.627	P=0.000
		WT 6h n=4		df=4 p=0.129	df1=1 df2=5 p=0.293		$F_{time(3,21)}$=38.638	P=0.000
		Nptx2 cKO 6h n=3		df=3 p=0.794				
		WT 24h n=4		df=4 p=0.967	df1=1 df2=5 p=0.913			
		Nptx2 cKO 24h n=3		df=3 p=0.308				
		WT 72h n=4		df=4 p=0.216	df1=1 df2=5 p=0.161			
		Nptx2 cKO 72h n=3		df=3 p=0.898				
		1.5h WT vs Nptx2 cKO						P=0.078

		6h WT vs Nptx2 cKO						P=0.084
		24h WT vs Nptx2 cKO						P=0.001
		72h WT vs Nptx2 cKO						P=0.000
		WT 1.5h vs 6h						P=0.201
		WT 1.5h vs 24h						P=0.690
		WT 1.5h vs 72h						P=0.210
		Nptx2 cKO 1.5h vs 6h						P=0.063
		Nptx2 cKO 1.5h vs 24h						P=0.001
		Nptx2 cKO 1.5h vs 72h						P=0.000
S5g	Intensity	WT 1.5h n=3	mouse	df=3 p=0.987	df1=1 df2=4 p=0.199	Two-way ANOVA	$F_{time\ x\ mice(3, 19)}$=8.384	P=0.001
		Nptx2 cKO 1.5h n=3		df=3 p=0.513			$F_{mice(1, 19)}$=23.660	P=0.000
		WT 6h n=4		df=4 p=0.629	df1=1 df2=5 p=0.096		$F_{time(3, 19)}$=35.294	P=0.000

		Nptx2 cKO 6h n=3		df=3 p=0.525				
		WT 24h n=4		df=4 p=0.255	df1=1 df2=5 p=1.000			
		Nptx2 cKO 24h n=3		df=3 p=0.213				
		WT 72h n=4		df=4 p=0.195	df1=1 df2=5 p=0.085			
		Nptx2 cKO 72h n=3		df=3 p=0.710				
		1.5h WT vs Nptx2 cKO						P=0.799
		6h WT vs Nptx2 cKO						P=0.532
		24h WT vs Nptx2 cKO						P=0.031
		72h WT vs Nptx2 cKO						P=0.000
		WT 1.5h vs 6h						P=0.074
		WT 1.5h vs 24h						P=0.074
		WT 1.5h vs 72h						P=0.074
		Nptx2 cKO 1.5h vs 6h						P=0.079
		Nptx2 cKO 1.5h vs 24h						P=0.079

		Nptx2 cKO 1.5h vs 72h						P=0.079
S6b	(dsRed+SST)/SST	F-RAM n=4<br>N-RAM n=4	mouse	df=4 p=0.990<br>df=4 p=0.776	df1=1 df2=6 p=0.995	Two-tailed unpaired t-test	t=-0.045	P=0.965
S6h	PPR	WT n=18 (from 3 mice)<br>Nptx1 cko n=14 (from 3 mice)	neuron	df=18 p=0.000<br>df=14 p=0.000	df1=1 df2=30 p=0.591	Mann-Whitney U test	Z=-3.799	P=0.000
S6j	A/N	WT n=19 (from 3 mice)<br>Nptx1 cko n=16 (from 3 mice)	neuron	df=19 p=0.116<br>df=16 p=0.222	df1=1 df2=33 p=0.692	Two-tailed unpaired t-test	t=-0.119	P=0.906
S6l	PPR	WT n=22 (from 4 mice)<br>Nptx2 cko n=20 (from 4 mice)	neuron	df=22 p=0.891<br>df=20 p=0.971	df1=1 df2=40 p=0.008	Two-tailed unpaired t-test	t=-6.099	P=0.000

S6n	A/N	WT n=19 (from 4 mice)	neuron	df=19 p=0.945	df1=1 df2=34 p=0.568	Two-tailed unpaired t-test	t=-0.174	P=0.863
		Nptx2 cko n=17 (from 4 mice)		df=17 p=0.153				
S7c	PPR	WT n=12 (from 4 mice)	neuron	df=12 p=0.008	df1=1 df2=24 p=0.000	Mann-Whitney U test	Z=-2.932	P=0.003
		Nptx1 cko n=14 (from 4 mice)		df=14 p=0.008				
S7e	PPR	WT n=16 (from 3 mice)	neuron	df=16 p=0.095	df1=1 df2=30 p=0.127	Mann-Whitney U test	Z=-2.940	P=0.003
		Nptx2 cko n=16 (from 5 mice)		df=16 p=0.004				
S8c	PPR	WT n=18 (from 3 mice)	neuron	df=18 p=0.035	df1=1 df2=32 p=0.062	Mann-Whitney U test	Z=-3.692	P=0.000
		Nptx1 cko n=16 (from 4 mice)		df=16 p=0.063				

S8e	A/N	WT n=18 (from 3 mice)	neuron	df=18 p=0.002	df1=1 df2=33 p=0.635	Mann-Whitney U test	Z=-0.165	P=0.883
		Nptx1 cko n=17 (from 4 mice)		df=17 p=0.001				
S8g	PPR	WT n=13 (from 4 mice)	neuron	df=13 p=0.038	df1=1 df2=26 p=0.035	Mann-Whitney U test	Z=-2.741	P=0.005
		Nptx2 cko n=15 (from 4 mice)		df=15 p=0.012				
S8i	A/N	WT n=17 (from 4 mice)	neuron	df=17 p=0.001	df1=1 df2=34 p=0.259	Mann-Whitney U test	Z=-0.650	P=0.531
		Nptx2 cko n=19 (from 4 mice)		df=19 p=0.016				
S8l	PPR	WT n=13 (from 3 mice)	neuron	df=13 p=0.354	df1=1 df2=29 p=0.381	Two-tailed unpaired t-test	t=-4.702	P=0.000
		Nptx1 cko n=18 (from 4 mice)		df=18 p=0.084				

S8n	A/N	WT n=12 (from 3 mice)	neuron	df=12 p=0.149	df1=1 df2=26 p=0.721	Two-tailed unpaired t-test	t=-0.232	P=0.818
		Nptx1 cko n=16 (from 4 mice)		df=16 p=0.210				
S8p	PPR	WT n=18 (from 4 mice)	neuron	df=18 p=0.002	df1=1 df2=34 p=0.897	Mann-Whitney U test	Z=-2.246	P=0.024
		Nptx2 cko n=18 (from 4 mice)		df=18 p=0.294				
S8r	A/N	WT n=16 (from 4 mice)	neuron	df=16 p=0.049	df1=1 df2=29 p=0.852	Mann-Whitney U test	Z=-0.870	P=0.401
		Nptx2 cko n=15 (from 4 mice)		df=15 p=0.180				
S9e	PPR	WT n=15 (from 4 mice)	neuron	df=15 p=0.011	df1=1 df2=30 p=0.229	Mann-Whitney U test	Z=-2.058	P=0.040
		Nptx2 cko n=17 (from 4 mice)		df=17 p=0.280				
S9g	A/N	WT n=15 (from 4	neuron	df=15 p=0.450	df1=1 df2=30 p=0.627	Mann-Whitney U	Z=-0.019	P=0.985

		mice)				test		
		Nptx2 cko n=17 (from 4 mice)		df=17 p=0.003				
S10a	Freezing (%)	WT pre-shock n=14	mouse	df=14 p=0.000	df1=1 df2=23 p=0.695	Kruskal-Wallis H test	H=0.045	P=0.833
		Nptx1 cko pre-shock n=11		df=11 p=0.000				
		WT shock1 n=14		df=14 p=0.014	df1=1 df2=23 p=0.330		H=0.549	P=0.459
		Nptx1 cko shock1 n=11		df=11 p=0.004				
		WT shock2 n=14		df=14 p=0.410	df1=1 df2=23 p=0.515		H=0.363	P=0.547
		Nptx1 cko shock2 n=11		df=11 p=0.673				
		WT shock3 n=14		df=14 p=0.027	df1=1 df2=23 p=0.269		H=0.147	P=0.702
		Nptx1 cko shock3 n=11		df=11 p=0.776				
S10b	Freezing (%)	WT pre-shock n=14	mouse	df=14 p=0.001	df1=1 df2=25 p=0.317	Kruskal-Wallis H test	H=1.678	P=0.195
		Nptx2 cko pre-shock		df=13 p=0.002				

		n=13						
		WT shock1 n=14		df=14 p=0.668	df1=1 df2=25 p=0.857		H=0.462	P=0.497
		Nptx2 cko shock1 n=13		df=13 p=0.006				
		WT shock2 n=14		df=14 p=0.645	df1=1 df2=25 p=0.881		H=0.850	P=0.357
		Nptx2 cko shock2 n=13		df=13 p=0.858				
		WT shock3 n=14		df=14 p=0.419	df1=1 df2=25 p=0.449		H=0.763	P=0.382
		Nptx2 cko shock3 n=13		df=13 p=0.869				
s10c	Freezing (%)	WT pre-shock n=18	mouse	df=18 p=0.000	df1=1 df2=31 p=0.526	Kruskal-Wallis H test	H=0.309	P=0.578
		Nptx1 cko pre-shock n=15		df=15 p=0.000				
		WT shock1 n=18		df=18 p=0.018	df1=1 df2=31 p=0.019		H=0.095	P=0.759
		Nptx1 cko shock1 n=15		df=15 p=0.426				
		WT shock2 n=18		df=18 p=0.907	df1=1 df2=31 p=0.876		H=0.221	P=0.638

		Nptx1 cko shock2 n=15		df=15 p=0.397				
		WT shock3 n=18		df=18 p=0.416	df1=1 df2=31 p=0.250		H=0.335	P=0.563
		Nptx1 cko shock3 n=15		df=15 p=0.060				
S10d	Freezing (%)	WT pre-shock n=13	mouse	df=13 p=0.012	df1=1 df2=23 p=0.044	Kruskal-Wallis H test	H=1.360	P=0.244
		Nptx2 cko pre-shock n=12		df=12 p=0.002				
		WT shock1 n=13		df=13 p=0.143	df1=1 df2=23 p=0.130		H=0.090	P=0.765
		Nptx2 cko shock1 n=12		df=12 p=0.848				
		WT shock2 n=13		df=13 p=0.842	df1=1 df2=23 p=0.137		H=0.189	P=0.663
		Nptx2 cko shock2 n=12		df=12 p=0.910				
		WT shock3 n=13		df=13 p=0.340	df1=1 df2=23 p=0.793		H=0.500	P=0.480
		Nptx2 cko shock3 n=12		df=12 p=0.625				
S10e	Freezing (%)	WT pre-shock n=11	mouse	df=11 p=0.000	df1=1 df2=20 p=0.311	Kruskal-Wallis H	H=0.907	P=0.341

		Nptx1 cko pre-shock n=11		df=11 p=0.000		test		
		WT shock1 n=11		df=11 p=0.001	df1=1 df2=20 p=0.812		H=0.156	P=0.693
		Nptx1 cko shock1 n=11		df=11 p=0.055				
		WT shock2 n=11		df=11 p=0.984	df1=1 df2=20 p=0.126		H=0.907	P=0.341
		Nptx1 cko shock2 n=11		df=11 p=0.331				
		WT shock3 n=11		df=11 p=0.739	df1=1 df2=20 p=0.596		H=0.087	P=0.768
		Nptx1 cko shock3 n=11		df=11 p=0.964				
S10f	Freezing (%)	WT pre-shock n=10	mouse	df=10 p=0.000	df1=1 df2=17 p=0.588	Kruskal-Wallis H test	H=0.480	P=0.488
		Nptx2 cko pre-shock n=9		df=9 p=0.000				
		WT shock1 n=10		df=10 p=0.421	df1=1 df2=17 p=0.797		H=0.002	P=0.967
		Nptx2 cko shock1 n=9		df=9 p=0.183				
		WT shock2 n=10		df=10 p=0.512	df1=1 df2=17 p=0.834		H=0.327	P=0.568

		Nptx2 cko shock2 n=9		df=9 p=0.197				
		WT shock3 n=10		df=10 p=0.897	df1=1 df2=17 p=0.051		H=0.107	P=0.744
		Nptx2 cko shock3 n=9		df=9 p=0.502				
S11d	Freezing (%)	WT pre-shock n=10	mouse	df=10 p=0.111	df1=1 df2=21 p=0.279	Kruskal-Wallis H test	H=0.495	P=0.482
		Nptx1 cko pre-shock n=13		df=13 p=0.004				
		WT shock1 n=10		df=10 p=0.496	df1=1 df2=21 p=0.379		H=0.062	P=0.804
		Nptx1 cko shock1 n=13		df=13 p=0.873				
		WT shock2 n=10		df=10 p=0.539	df1=1 df2=21 p=0.450		H=0.138	P=0.710
		Nptx1 cko shock2 n=13		df=13 p=0.363				
		WT shock3 n=10		df=10 p=0.263	df1=1 df2=21 p=0.630		H=0.138	P=0.710
		Nptx1 cko shock3 n=13		df=13 p=0.351				
S11e	Freezing (%)	WT context A n=10	mouse	df=10 p=0.902	df1=1 df2=21 p=0.567	Kruskal-Wallis H	H=5.265	P=0.022

		Nptx1 cko context A n=13		df=13 p=0.964		test		
		WT context C n=10		df=10 p=0.621	df1=1 df2=21 p=0.647		H=1.388	P=0.239
		Nptx1 context C n=13		df=13 p=0.029				
S11f	Discrimination index	WT n=10	mouse	df=10 p=0.126	df1=1 df2=21 p=0.497	Two-tailed unpaired t-test	t=0.193	P=0.849
		Nptx1 cko n=13		df=13 p=0.999				
S11h	Discrimination index	WT n=10	mouse	df=10 p=0.833	df1=1 df2=21 p=0.710	Two-tailed unpaired t-test	t=-0.081	P=0.936
		Nptx1 cko n=13		df=13 p=0.652				
S11j	Discrimination index	WT n=10	mouse	df=10 p=0.304	df1=1 df2=21 p=0.152	Two-tailed unpaired t-test	t=0.332	P=0.743
		Nptx1 cko n=13		df=13 p=0.105				
S11n	Freezing (%)	WT pre-shock n=12	mouse	df=12 p=0.017	df1=1 df2=23 p=0.403	Kruskal-Wallis H test	H=1.081	P=0.298
		Nptx2 cko pre-shock n=13		df=13 p=0.001				
		WT shock1 n=12		df=12 p=0.226	df1=1 df2=23 p=0.259		H=0.580	P=0.446
		Nptx2 cko shock1 n=13		df=13 p=0.325				

		WT shock2 n=12		df=12 p=0.325	df1=1 df2=23 p=0.571		H=0.240	P=0.624
		Nptx2 cko shock2 n=13		df=13 p=0.238				
		WT shock3 n=12		df=12 p=0.455	df1=1 df2=23 p=0.415		H=0.047	P=0.828
		Nptx2 cko shock3 n=13		df=13 p=0.938				
S11o	Freezing (%)	WT context A n=12	mouse	df=12 p=0.156	df1=1 df2=23 p=0.514	Two-way RM ANOVA	$F_{Treatment \times context(1, 23)}=3.028$	P=0.095
		Nptx2 cko context A n=13		df=13 p=0.333			$F_{context(1, 23)}=3.028$	P=0.000
		WT context C n=12		df=12 p=0.670	df1=1 df2=23 p=0.091		$F_{Treatment(1, 25)}=2.145$	P=0.157
		Nptx2 context C n=13		df=13 p=0.747				
		WT vs Nptx2 cko context A						P=0.829
		WT vs Nptx2 cko context C						P=0.020

S11p	Discrimination index	WT n=12	mouse	df=12 p=0.139	df1=1 df2=23 p=0.620	Two-tailed unpaired t-test	t=2.095	P=0.047
		Nptx2 cko n=13		df=13 p=0.107				
S11r	Discrimination index	WT n=12	mouse	df=12 p=0.065	df1=1 df2=23 p=0.374	Two-tailed unpaired t-test	t=0.228	P=0.822
		Nptx2 cko n=13		df=13 p=0.196				
S11t	Discrimination index	WT n=12	mouse	df=12 p=0.328	df1=1 df2=23 p=0.085	Two-tailed unpaired t-test	t=0.085	P=0.681
		Nptx2 cko n=13		df=13 p=0.671				
S12b left	Distance	WT n=10	mouse	df=10 p=0.068	df1=1 df2=18 p=0.780	Two-tailed unpaired t-test	t=-0.508	P=0.617
		Nptx1 cko n=10		df=10 p=0.442				
S12b right	Time	WT n=10	mouse	df=10 p=0.100	df1=1 df2=18 p=0.008	Two-tailed unpaired t-test	t=0.627	P=0.539
		Nptx1 cko n=10		df=10 p=0.500				
S12c left	Distance	WT n=10	mouse	df=10 p=0.705	df1=1 df2=19 p=0.807	Two-tailed unpaired t-test	t=-0.800	P=0.434
		Nptx1 cko n=11		df=11 p=0.593				
S12c right	Time	WT n=10	mouse	df=10 p=0.594	df1=1 df2=19 p=0.865	Two-tailed unpaired t-test	t=-0.449	P=0.658
		Nptx1 cko n=11		df=11 p=0.692				
S12d left	Time	WT n=10	mouse	df=10 p=0.018	df1=1 df2=19 p=0.898	Mann-Whitney U test	Z=-0.070	P=0.973
		Nptx1 cko n=11		df=11 p=0.154				
S12d right	Latency	WT n=10	mouse	df=10 p=0.994	df1=1 df2=19 p=0.070	Two-tailed	t=-0.050	P=0.960

		Nptx1 cko n=11		df=11 p=0.085		unpaired t-test		
S12e left	Distance	WT n=15<br>Nptx2 cko n=17	mouse	df=15 p=0.880<br>df=17 p=0.016	df1=1 df2=30 p=0.143	Mann-Whitney U test	Z=-0.359	P=0.737
S12e right	Time	WT n=15<br>Nptx2 cko n=17	mouse	df=15 p=0.901<br>df=17 p=0.261	df1=1 df2=30 p=0.860	Two-tailed unpaired t-test	t=0.116	P=0.908
S12f left	Distance	WT n=17<br>Nptx2 cko n=17	mouse	df=17 p=0.998<br>df=17 p=0.015	df1=1 df2=32 p=0.962	Mann-Whitney U test	Z=-1.361	P=0.182
S12f right	Time	WT n=17<br>Nptx2 cko n=17	mouse	df=17 p=0.404<br>df=17 p=0.415	df1=1 df2=32 p=0.337	Two-tailed unpaired t-test	t=-0.256	P=0.800
S12g left	Time	WT n=13<br>Nptx2 cko n=15	mouse	df=13 p=0.026<br>df=15 p=0.983	df1=1 df2=26 p=0.199	Mann-Whitney U test	Z=-0.530	P=0.618
S12g right	Latency	WT n=13<br>Nptx2 cko n=15	mouse	df=13 p=0.908<br>df=15 p=0.001	df1=1 df2=26 p=0.305	Mann-Whitney U test	Z=-0.645	P=0.525
S13b left	Distance	WT n=20<br>Nptx1 cko n=20	mouse	df=20 p=0.896<br>df=20 p=0.389	df1=1 df2=38 p=0.499	Two-tailed unpaired t-test	t=-0.566	P=0.575
S13b right	Time	WT n=20<br>Nptx1 cko n=20	mouse	df=20 p=0.501<br>df=20 p=0.501	df1=1 df2=38 p=0.316	Two-tailed unpaired t-test	t=-0.042	P=0.966

S13c left	Distance	WT n=20<br>Nptx1 cko n=20	mouse	df=20 p=0.333<br>df=20 p=0.783	df1=1 df2=38 p=0.488	Two-tailed unpaired t-test	t=-0.478	P=0.636
S13c right	Time	WT n=20<br>Nptx1 cko n=20	mouse	df=20 p=0.521<br>df=20 p=0.007	df1=1 df2=38 p=0.396	Mann-Whitney U test	Z=-0.380	P=0.174
S13d left	Time	WT n=19<br>Nptx1 cko n=17	mouse	df=19 p=0.158<br>df=17 p=0.469	df1=1 df2=34 p=0.256	Two-tailed unpaired t-test	t=0.294	P=0.770
S13d right	Latency	WT n=19<br>Nptx1 cko n=17	mouse	df=19 p=0.770<br>df=17 p=0.058	df1=1 df2=34 p=0.495	Two-tailed unpaired t-test	t=0.111	P=0.912
S13e left	Distance	WT n=10<br>Nptx2 cko n=10	mouse	df=10 p=0.705<br>df=10 p=0.021	df1=1 df2=18 p=0.833	Mann-Whitney U test	Z=-1.285	P=0.218
S13e right	Time	WT n=10<br>Nptx2 cko n=10	mouse	df=10 p=0.364<br>df=10 p=0.365	df1=1 df2=18 p=0.892	Two-tailed unpaired t-test	t=0.197	P=0.846
S13f left	Distance	WT n=13<br>Nptx2 cko n=12	mouse	df=13 p=0.755<br>df=12 p=0.958	df1=1 df2=23 p=0.671	Two-tailed unpaired t-test	t=0.350	P=0.730
S13f right	Time	WT n=13<br>Nptx2 cko n=12	mouse	df=13 p=0.204<br>df=12 p=0.437	df1=1 df2=23 p=0.008	Two-tailed unpaired t-test	t=-0.606	P=0.550
S13g left	Time	WT n=12	mouse	df=12 p=0.027	df1=1 df2=22 p=0.859	Mann-	Z=0.111	P=0.876

		Nptx2 cko n=12		df=12 p=0.032		Whitney U test		
S13g right	Latency	WT n=12<br>Nptx2 cko n=12	mouse	df=12 p=0.557<br>df=12 p=0.583	df1=1 df2=22 p=0.428	Two-tailed unpaired t-test	t=0.005	P=0.996
S14b left	Distance	WT n=12<br>Nptx1 cko n=11	mouse	df=12 p=0.947<br>df=11 p=0.389	df1=1 df2=21 p=0.487	Two-tailed unpaired t-test	t=0.073	P=0.942
S14b right	Time	WT n=12<br>Nptx1 cko n=11	mouse	df=12 p=0.645<br>df=11 p=0.844	df1=1 df2=21 p=0.024	Two-tailed unpaired t-test	t=-0.733	P=0.472
S14c left	Distance	WT n=13<br>Nptx1 cko n=14	mouse	df=13 p=0.969<br>df=14 p=0.080	df1=1 df2=25 p=0.331	Two-tailed unpaired t-test	t=1.673	P=0.107
S14c right	Time	WT n=13<br>Nptx1 cko n=14	mouse	df=13 p=0.741<br>df=14 p=0.284	df1=1 df2=25 p=0.762	Two-tailed unpaired t-test	t=-0.722	P=0.477
S14d left	Time	WT n=11<br>Nptx1 cko n=13	mouse	df=11 p=0.178<br>df=13 p=0.461	df1=1 df2=22 p=0.091	Two-tailed unpaired t-test	t=-0.443	P=0.662
S14d right	Latency	WT n=11<br>Nptx1 cko n=13	mouse	df=11 p=0.760<br>df=13 p=0.891	df1=1 df2=22 p=0.868	Two-tailed unpaired t-test	t=-0.208	P=0.837
S14e left	Distance	WT n=10<br>Nptx2 cko n=9	mouse	df=10 p=0.280<br>df=9 p=0.198	df1=1 df2=17 p=0.400	Two-tailed unpaired t-test	t=0.372	P=0.715

S14e right	Time	WT n=10	mouse	df=10 p=0.343	df1=1 df2=17 p=0.702	Two-tailed unpaired t-test	t=0.632	P=0.536
		Nptx2 cko n=9		df=9 p=0.231				
S14f left	Distance	WT n=10	mouse	df=10 p=0.669	df1=1 df2=17 p=0.162	Two-tailed unpaired t-test	t=1.321	P=0.204
		Nptx2 cko n=9		df=9 p=0.509				
S14f right	Time	WT n=10	mouse	df=10 p=0.911	df1=1 df2=17 p=0.301	Two-tailed unpaired t-test	t=0.743	P=0.467
		Nptx2 cko n=9		df=9 p=0.487				
S14g left	Time	WT n=9	mouse	df=9 p=0.971	df1=1 df2=17 p=0.470	Two-tailed unpaired t-test	t=-0.529	P=0.603
		Nptx2 cko n=10		df=10 p=0.450				
S14g right	Latency	WT n=9	mouse	df=9 p=0.622	df1=1 df2=17 p=0.052	Two-tailed unpaired t-test	t=0.205	P=0.840
		Nptx2 cko n=10		df=10 p=0.977				
S15a	Freezing (%)	WT-vehicle pre-shock n=12	mouse	df=12 p=0.000	df1=3 df2=43 p=0.207	Kruskal-Wallis H test	H=5.701	P=0.127
		WT-retigabine pre-shock n=13		df=13 p=0.001				
		Nptx1 cko-vehicle pre-shock n=10		df=10 p=0.019				

		Nptx1 cko-retigabine pre-shock n=12		df=12 p=0.000				
		WT-vehicle shock1 n=12		df=12 p=0.186	df1=3 df2=43 p=0.137		H=1.340	P=0.720
		WT-retigabine shock1 n=13		df=13 p=0.017				
		Nptx1 cko-vehicle shock1 n=10		df=10 p=0.073				
		Nptx1 cko-retigabine shock1 n=12		df=12 p=0.021				
		WT-vehicle shock2 n=12		df=12 p=0.527	df1=3 df2=43 p=0.874		H=1.104	P=0.776
		WT-retigabine shock2 n=13		df=13 p=0.019				
		Nptx1 cko-vehicle shock2 n=10		df=10 p=0.417				
		Nptx1 cko-retigabine shock2 n=12		df=12 p=0.623				

		WT-vehicle shock3 n=12		df=12 p=0.740	df1=3 df2=43 p=0.449		H=0.558	P=0.906
		WT-retigabine shock3 n=13		df=13 p=0.705				
		Nptx1 cko-vehicle shock3 n=10		df=10 p=0.619				
		Nptx1 cko-retigabine shock3 n=12		df=12 p=0.797				
S15b	Freezing (%)	WT-vehicle pre-shock n=10	mouse	df=10 p=0.817	df1=3 df2=38 p=0.098	Kruskal-Wallis H test	H=3.327	P=0.344
		WT-retigabine pre-shock n=11		df=11 p=0.447				
		Nptx1 cko-vehicle pre-shock n=11		df=11 p=0.103				
		Nptx1 cko-retigabine pre-shock n=10		df=10 p=0.045				

		WT-vehicle shock1 n=10		df=10 p=0.488	df1=3 df2=38 p=0.707		H=0.301	P=0.960
		WT-retigabine shock1 n=11		df=11 p=0.020				
		Nptx1 cko-vehicle shock1 n=11		df=11 p=0.829				
		Nptx1 cko-retigabine shock1 n=10		df=10 p=0.666				
		WT-vehicle shock2 n=10		df=10 p=0.879	df1=3 df2=38 p=0.240		H=0.335	P=0.953
		WT-retigabine shock2 n=11		df=11 p=0.315				
		Nptx1 cko-vehicle shock2 n=11		df=11 p=0.203				
		Nptx1 cko-retigabine shock2 n=10		df=10 p=0.978				
		WT-vehicle shock3 n=10		df=10 p=0.293	df1=3 df2=38 p=0.211		H=0.558	P=0.906
		WT-retigabine		df=11 p=0.048				

		shock3 n=11						
		Nptx1 cko-vehicle shock3 n=11		df=11 p=0.801				
		Nptx1 cko-retigabine shock3 n=10		df=10 p=0.494				
S15c	Freezing (%)	Scramble-mcherry pre-shock n=10	mouse	df=10 p=0.040	df1=3 df2=39 p=0.073	Kruskal-Wallis H test	H=1.406	P=0.704
		Scramble-hM3Dq pre-shock n=11		df=11 p=0.290				
		Nptx2 sh-mcherry pre-shock n=10		df=10 p=0.039				
		Nptx2 sh-hM3Dq pre-shock n=12		df=12 p=0.268				
		Scramble-mcherry shock1 n=10		df=10 p=0.808	df1=3 df2=39 p=0.932		H=1.714	P=0.634

		Scramble-hM3Dq shock1 n=11		df=11 p=0.105				
		Nptx2 sh-mcherry shock1 n=10		df=10 p=0.806				
		Nptx2 sh-hM3Dq shock1 n=12		df=12 p=0.236				
		Scramble-mcherry shock2 n=10		df=10 p=0.022	df1=3 df2=39 p=0.073		H=0.353	P=0.950
		Scramble-hM3Dq shock2 n=11		df=11 p=0.677				
		Nptx2 sh-mcherry shock2 n=10		df=10 p=0.403				
		Nptx2 sh-hM3Dq shock2 n=12		df=12 p=0.595				
		Scramble-mcherry shock3 n=10		df=10 p=0.147	df1=3 df2=39 p=0.332		H=0.669	P=0.880

		Scramble-hM3Dq shock3 n=11		df=11 p=0.639				
		Nptx2 sh-mcherry shock3 n=10		df=10 p=0.119				
		Nptx2 sh-hM3Dq shock3 n=12		df=12 p=0.300				
S15d	Freezing (%)	Scramble-mcherry pre-shock n=16	mouse	df=16 p=0.000	df1=3 df2=56 p=0.693	Kruskal-Wallis H test	H=0.088	P=0.993
		Scramble-hM3Dq pre-shock n=15		df=15 p=0.000				
		Nptx2 sh-mcherry pre-shock n=15		df=15 p=0.000				
		Nptx2 sh-hM3Dq pre-shock n=14		df=14 p=0.000				
		Scramble-mcherry shock1 n=16		df=16 p=0.064	df1=3 df2=56 p=0.823		H=0.602	P=0.896

		Scramble-hM3Dq shock1 n=15		df=15 p=0.129				
		Nptx2 sh-mcherry shock1 n=15		df=15 p=0.120				
		Nptx2 sh-hM3Dq shock1 n=14		df=14 p=0.009				
		Scramble-mcherry shock2 n=16		df=16 p=0.768	df1=3 df2=56 p=0.621		H=0.975	P=0.807
		Scramble-hM3Dq shock2 n=15		df=15 p=0.535				
		Nptx2 sh-mcherry shock2 n=15		df=15 p=0.142				
		Nptx2 sh-hM3Dq shock2 n=14		df=14 p=0.169				
		Scramble-mcherry shock3 n=16		df=16 p=0.450	df1=3 df2=56 p=0.188		H=0.273	P=0.965

		Scramble-hM3Dq shock3 n=15		df=15 p=0.136				
		Nptx2 sh-mcherry shock3 n=15		df=15 p=0.275				
		Nptx2 sh-hM3Dq shock3 n=14		df=14 p=0.149				
S17a	Relative protein quantification	Young n=4	mouse	df=4 p=0.303	df1=1 df2=7 p=0.185	Two-tailed unpaired t-test	t=-0.947	P=0.375
		Aged n=5		df=5 p=0.387				
S17b	Relative mRNA expression	EYFP n=10	mouse	df=10 p=0.331	df1=1 df2=15 p=0.004	Two-tailed unpaired t-test	t=-14.296	P=0.000
		Nptx1 n=7		df=7 p=0.123				
S17c	Freezing (%)	EYFP preshock n=11	mouse	df=11 p=0.000	df1=1 df2=21 p=0.002	Kruskal-Wallis H test	H=3.054	P=0.081
		Nptx1 preshock n=12		df=12 p=0.000				
		EYFP shock n=11		df=11 p=0.018	df1=1 df2=21 p=0.277		H=0.640	P=0.424
		Nptx1 shock n=12		df=12 p=0.664				

S17d	Freezing (%)	EYFP preshock n=14	mouse	df=14 p=0.001	df1=1 df2=21 p=0.227	Kruskal-Wallis H test	H=0.237	P=0.626
		Nptx1 preshock n=9		df=9 p=0.008				
		EYFP shock n=14		df=14 p=0.217	df1=1 df2=21 p=0.590		H=0.120	P=0.729
		Nptx1 shock n=9		df=9 p=0.183				
S17e	Freezing (%)	EGFP pre-shock n=11	mouse	df=11 p=0.040	df1=1 df2=20 p=0.369	Kruskal-Wallis H test	H=2.997	P=0.083
		Nptx2-PTX pre-shock n=11		df=11 p=0.000				
		EGFP shock1 n=11		df=11 p=0.949	df1=1 df2=20 p=0.493		H=0.087	P=0.768
		Nptx2-PTX shock1 n=11		df=11 p=0.779				
		EGFP shock2 n=11		df=11 p=0.257	df1=1 df2=20 p=0.665		H=0.570	P=0.450
		Nptx2-PTX shock2 n=11		df=11 p=0.513				
		EGFP shock3 n=11		df=11 p=0.689	df1=1 df2=20 p=0.761		H=0.027	P=0.870

		Nptx2-PTX shock3 n=11		df=11 p=0.212				
S17f	Freezing (%)	EGFP pre-shock n=13	mouse	df=13 p=0.006	df1=1 df2=21 p=0.209	Kruskal-Wallis H test	H=4.444	P=0.035
		Nptx2-PTX pre-shock n=10		df=10 p=0.000				
		EGFP shock1 n=13		df=13 p=0.124	df1=1 df2=21 p=0.903		H=0.465	P=0.495
		Nptx2-PTX shock1 n=10		df=10 p=0.938				
		EGFP shock2 n=13		df=13 p=0.400	df1=1 df2=21 p=0.179		H=0.465	P=0.495
		Nptx2-PTX shock2 n=10		df=10 p=0.477				
		EGFP shock3 n=13		df=13 p=0.300	df1=1 df2=21 p=0.637		H=0.062	P=0.804
		Nptx2-PTX shock3 n=10		df=10 p=0.146				
S18d	Freezing (%)	EYFP preshock n=11	mouse	df=11 p=0.000	df1=1 df2=19 p=0.731	Kruskal-Wallis H test	H=0.113	P=0.737
		Nptx1 preshock n=10		df=10 p=0.000				

		EYFP shock n=11		df=11 p=0.022	df1=1 df2=19 p=0.146		H=0.031	P=0.860
		Nptx1 shock n=10		df=10 p=0.462				
S18e	Freezing (%)	EYFP context A n=11	mouse	df=11 p=0.999	df1=1 df2=19 p=0.552	Two-way RM ANOVA	$F_{Treatment \times context(1, 19)}=0.039$	P=0.845
		Nptx1 context A n=10		df=10 p=0.193			$F_{Treatment(1, 19)}=0.036$	P=0.853
		EYFP context C n=11		df=11 p=0.791	df1=1 df2=19 p=0.402		$F_{context(1, 19)}=7.556$	P=0.013
		Nptx1 context C n=10		df=10 p=0.702				
		EYFP vs Nptx1 context A						P=0.938
		EYFP vs Nptx1 context C						P=0.807
S18f	Discrimination index	EYFP n=11	mouse	df=11 p=0.756	df1=1 df2=19 p=0.509	Two-tailed unpaired t-test	t=0.327	P=0.748
		Nptx1 n=10		df=10 p=0.075				

S18g	Freezing (%)	EYFP preshock n=10	mouse	df=10 p=0.003	df1=1 df2=17 p=0.699	Kruskal-Wallis H test	H=0.281	P=0.596
		Nptx1 preshock n=9		df=9 p=0.023				
		EYFP shock n=10		df=10 p=0.235	df1=1 df2=17 p=0.426		H=0.060	P=0.806
		Nptx1 shock n=9		df=9 p=0.248				
S18h	Freezing (%)	EYFP context A n=10	mouse	df=10 p=0.241	df1=1 df2=17 p=0.904	Two-way RM ANOVA	$F_{Treatment \times context(1, 17)}=0.009$	P=0.927
		Nptx1 context A n=9		df=9 p=0.812			$F_{Treatment(1, 17)}=0.019$	P=0.893
		EYFP context C n=10		df=10 p=0.282	df1=1 df2=17 p=0.626		$F_{context(1, 17)}=16.739$	P=0.001
		Nptx1 context C n=9		df=9 p=0.622				
		EYFP vs Nptx1 context A						P=0.948

		EYFP vs Nptx1 context C						P=0.844
S18i	Discrimination index	EYFP n=10	mouse	df=10 p=0.288	df1=1 df2=17 p=0.762	Two-tailed unpaired t-test	t=-0.236	P=0.816
		Nptx1 n=9		df=9 p=0.406				
S18m	Freezing (%)	EGFP pre-shock n=10	mouse	df=10 p=0.000	df1=1 df2=19 p=0.021	Kruskal-Wallis H test	H=3.616	P=0.057
		Nptx2-PTX pre-shock n=11		df=11 p=0.062				
		EGFP shock1 n=10		df=10 p=0.315	df1=1 df2=19 p=0.139		H=0.715	P=0.398
		Nptx2-PTX shock1 n=11		df=11 p=0.014				
		EGFP shock2 n=10		df=10 p=0.494	df1=1 df2=19 p=0.422		H=0.496	P=0.481
		Nptx2-PTX shock2 n=11		df=11 p=0.234				
		EGFP shock3 n=10		df=10 p=0.025	df1=1 df2=19 p=0.757		H=0.045	P=0.833
		Nptx2-PTX shock3 n=11		df=11 p=0.831				

S18n	Freezing (%)	EGFP context A n=10	mouse	df=10 p=0.892	df1=1 df2=19 p=0.269	Two-way RM ANOVA	$F_{Treatment \times context(1, 19)}=0.132$	P=0.721
		Nptx2-PTX context A n=11		df=11 p=0.484			$F_{Treatment(1, 19)}=0.140$	P=0.712
		EGFP context C n=10		df=10 p=0.606	df1=1 df2=19 p=0.603		$F_{context(1, 19)}=101.391$	P=0.000
		Nptx2-PTX context C n=11		df=11 p=0.454				
		EYFP vs Nptx2-PTX context A						P=0.823
		EYFP vs Nptx2-PTX context C						P=0.620
S18o	Discrimination index	EGFP n=10	mouse	df=10 p=0.249	df1=1 df2=19 p=0.842	Two-tailed unpaired t-test	t=0.339	P=0.739
		Nptx2-PTX n=11		df=11 p=0.806				
S18p	Freezing (%)	EGFP pre-shock n=11	mouse	df=11 p=0.000	df1=1 df2=19 p=0.005	Kruskal-Wallis H test	H=2.090	P=0.148
		Nptx2-PTX pre-shock		df=10 p=0.045				

		n=10						
		EGFP shock1 n=11		df=11 p=0.153	df1=1 df2=19 p=0.634		H=0.079	P=0.778
		Nptx2-PTX shock1 n=10		df=10 p=0.188				
		EGFP shock2 n=11		df=11 p=0.189	df1=1 df2=19 p=0.375		H=0.001	P=0.972
		Nptx2-PTX shock2 n=10		df=10 p=0.593				
		EGFP shock3 n=11		df=11 p=0.937	df1=1 df2=19 p=0.008		H=0.179	P=0.673
		Nptx2-PTX shock3 n=10		df=10 p=0.152				
S18q	Freezing (%)	EGFP context A n=11	mouse	df=11 p=0.366	df1=1 df2=19 p=0.220	Two-way RM ANOVA	$F_{Treatment \times context(1, 19)}$=0.010	P=0.923
		Nptx2-PTX context A n=10		df=10 p=0.438			$F_{Treatment(1, 19)}$=0.360	P=0.555
		EGFP context C n=11		df=11 p=0.192	df1=1 df2=19 p=0.331		$F_{context(1, 19)}$=63.721	P=0.000
		Nptx2-PTX context C		df=10 p=0.615				

		n=10						
		EYFP vs Nptx2-PTX context A						P=0.496
		EYFP vs Nptx2-PTX context C						P=0.706
S18r	Discrimination index	EGFP n=11	mouse	df=11 p=0.703	df1=1 df2=19 p=0.279	Two-tailed unpaired t-test	t=-0.068	P=0.946
		Nptx2-PTX n=10		df=10 p=0.691				
